# Chromosome Missegregation in Single Human Oocytes Is Related to the Age and Gene Expression Profile

**DOI:** 10.3390/ijms21061934

**Published:** 2020-03-12

**Authors:** Stefano Barone, Patrizia Sarogni, Roberto Valli, Maria Michela Pallotta, Gazzi Silvia, Annalisa Frattini, Abdul Waheed Khan, Erika Rapalini, Cristiana Parri, Antonio Musio

**Affiliations:** 1 Centro Procreazione Assistita Ospedale Versilia, Unità Sanitaria Locale USL Toscana Nordovest, 55041 Lido di Camaiore, Italy; stefano.barone@uslnordovest.toscana.it (S.B.); silvia.gazzi@uslnordovest.toscana.it (G.S.); erika.rapalini@uslnordovest.toscana.it (E.R.); cristiana.parri@uslnordovest.toscana.it (C.P.); 2 Institute for Genetic and Biomedical Research (IRGB), National Research Council (CNR), 56124 Pisa, Italy; patrizia.sarogni@irgb.cnr.it (P.S.); mariamichela.pallotta@irgb.cnr.it (M.M.P.); 3 Medical Genetics and Applied Biology Unit, Department of Medicine and Surgery, University of Insubria, 21100 Varese, Italy; roberto.valli@uninsubria.it (R.V.); annalisa.frattini@irgb.cnr.it (A.F.); khan_ibge@yahoo.com (A.W.K.); 4 Institute for Genetic and Biomedical Research (IRGB), National Research Council (CNR), 20090 Milan, Italy; 5 Life Sciences and Biotechnology program of the XXXIII cycle, University of Insubria, 21100 Varese, Italy

**Keywords:** ovarian aging, cumulus cells, gene expression profile, RNA-seq, chromosome aneuploidy, array CGH, GPCRs, REEP4

## Abstract

The growing trend for women to postpone childbearing has resulted in a dramatic increase in the incidence of aneuploid pregnancies. Despite the importance to human reproductive health, the events precipitating female age-related meiotic errors are poorly understood. To gain new insight into the molecular basis of age-related chromosome missegregation in human oocytes, we combined the transcriptome profiles of twenty single oocytes (derived from females divided into two groups according to age <35 and ≥35 years) with their chromosome status obtained by array comparative genomic hybridization (aCGH). Furthermore, we compared the transcription profile of the single oocyte with the surrounding cumulus cells (CCs). RNA-seq data showed differences in gene expression between young and old oocytes. Dysregulated genes play a role in important biological processes such as gene transcription regulation, cytoskeleton organization, pathways related to RNA maturation and translation. The comparison of the transcription profile of the oocyte and the corresponding CCs highlighted the differential expression of genes belonging to the G protein-coupled receptor superfamily. Finally, we detected the loss of a X chromosome in two oocytes derived from women belonging to the ≥35 years age group. These aneuploidies may be caused by the detriment of REEP4, an endoplasmic reticulum protein, in women aged ≥35 years. Here we gained new insight into the complex regulatory circuit between the oocyte and the surrounding CCs and uncovered a new putative molecular basis of age-related chromosome missegregation in human oocytes.

## 1. Introduction

An oocyte’s competence is related to its ability to complete meiotic maturation, undergo fertilization and sustain embryonic development [1,2,3]. The complex relationship between oocyte, endocrine system and cumulus oophorus cell complex (COC) is postulated to be important for the acquisition of competence [4]. In fact, the cumulus cells (CCs) modulate the transcriptional activity of the oocyte genome and support the cytoplasmatic maturation and the meiotic arrest of the oocyte by calcium and an elevated level of cyclic adenosine monophosphate (cAMP) [5,6,7,8]. In addition, the oocyte releases, through gap junctions, growth factors that promote CC differentiation and proliferation, preventing their transition to mural granulosa cells [7,9]. In the last few decades, the average age of first-time mothers has increased worldwide, essentially due to social and economic factors. In addition to progressive decay in the number of ovarian follicles, the decline in female fertility has been associated with more advanced female age. In the context of assisted reproductive treatment (ART), this phenomenon poses a serious medical problem because chromosome missegregation and aneuploidy increase with age, leading to infertility and miscarriage [10,11,12,13]. A few trisomies (13, 18 and 21) and a monosomy (X chromosome) are capable of surviving to term, resulting in newborns with congenital defects and cognitive impairment [14,15]. Experimental data indicates that the aneuploidy rate in the oocytes of women under 25 years old is about 5%, 10–25% in the early 30 s and increases to 50% in women over 40 years [16,17,18,19].

Two mechanisms are mainly responsible for aneuploidy in human oocytes. The first involves the non-disjunction of entire chromosomes. The second implicates the premature division of sister chromatids, upon completion of the first meiotic division, followed by random segregation [19,20]. Cohesin plays a pivotal role in ensuring correct chromosome segregation during meiotic division. The meiotic core cohesin complex is composed of four subunits: two structural maintenance of chromosomes (SMC) subunits, called SMC1B and SMC3, and two non-SMC subunits, called STAG3 and REC8. Oocytes are considered inactive transcriptional cells but they store large quantities of mRNA and proteins for months and years in an inactivated form. Thus, the observation that cohesion is almost exclusively dependent on prenatally synthesized cohesin raises the possibility that the long meiotic arrest of oocytes facilitates a deterioration of cohesion, leading to age-related increases in aneuploidy [21,22].

A number of strategies for evaluating oocyte quality have been proposed, including assessing oocyte cytoplasm, the surrounding CCs or the first polar body, but unfortunately these methods provide little information on oocyte competence. The analysis of transcriptome profiling of human oocytes is an alternative and interesting approach. In animal models, it has been observed that oocytes from young vs. old mice contain 5% differentially expressed genes [23]. Furthermore, a substantial difference in the transcriptional level has been detected between younger and older human oocytes, possibly contributing to a decrease in oocyte quality [24].

It has been suggested that the transcriptome profiles of CCs and oocytes could reflect the developmental potential for successful fertilization and embryo development [25,26,27,28,29,30]. However, little is known about the complex interplay between genes expressed in oocytes and the surrounding CCs and their relationship with the genome integrity of the oocytes. We sought to identify novel molecular markers of age-related aneuploidy that could be used for non-invasive screening. To this aim, we compared the transcriptome profiles (obtained by RNA-seq) of single oocytes derived from young (<35 years) and older women (≥35 years) in search of specific pathways critical for oocyte function. In addition, these profiles were compared with those obtained by the corresponding CCs. Dysregulated genes might expose the molecular basis underlying aneuploidy; thus, this genome-wide data was then related to chromosome status. 

## 2. Results

### 2.1. Differentially Expressed Genes in Relation to Age and Genome Integrity of Single Oocytes

**Figure 1:**
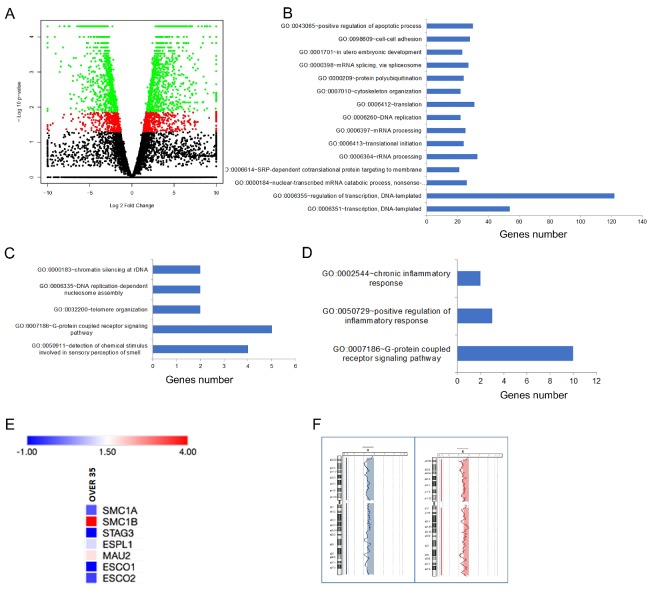
Gene expression profiles of single human oocytes. (**A**) A total of 1852 genes were differentially expressed in age group <35 vs. ≥35 years. Volcano plot of gene expression changes in oocytes <35 vs. ≥35 years. The *x*-axis displays the log2 fold change value and the *y*-axis corresponds to the negative logarithm to the base 10 of the *t*-test *p*-values. The black dots correspond to genes with no significant difference, the red dots represent dysregulated genes with a range of values with *p* < 0.05 and the green dots are significant with *p* < 0.05 (adjusted *p*-values). (**B**) GO term enrichment analysis of biological processes that were significantly over-represented when considering DEGS in oocytes <35 vs. ≥35 years. In particular, pathways related to gene transcription, apoptosis, cytoskeleton organization and to mRNA maturation and translation were over-represented. (**C**) GO term enrichment analysis of biological processes that were significantly over-represented in genes expressed exclusively in oocytes <35 years. (**D**) GO term enrichment analysis showed that the “G-protein coupled receptor signalling pathway” (GO:0007186) was significantly over-represented in genes expressed exclusively in oocytes ≥35 years. (**E**) Heat map of cohesin genes differentially expressed when oocytes <35 vs. ≥35 years were compared. Their fold change ranges from −1 to +4. The expression of *SMC1B* and *MAU2* increased whereas *ESCO1*, *ESCO2, ESPL1*, *SMC1A* and *STAG3* genes decreased in the ≥35 years age group. (**F**) Array comparative genomic hybridization analysis in 20 oocytes from women ranging from 19 to 42 years old. We identified the loss of the X chromosome in two subjects belonging to the ≥35 years age group.

In order to determine the effect of oocyte age on the human transcriptome, we analyzed the gene expression profiles of 20 single oocytes derived from 20 women subdivided into two age groups: <35 (9 oocytes) and ≥35 years (11 oocytes). Because different hormonal treatments did not affect gene expression (data not shown), RNA-seq data were pooled. Based on results of RNA sequencing, oocytes displayed gene expression dysregulation. In fact, 1852 genes were identified as differentially expressed genes (DEGs) in age group <35 vs. ≥35 (Table S1). Transcriptome changes can be observed in volcano plots reported in Figure 1A.

To establish the biological significance of DEGs, an overrepresentation test was performed by gene ontology (GO) enrichment analysis. The biological processes regarding gene transcription such as “regulation of transcription,” (GO:0006355 and GO:0006351) and “positive regulation of apoptotic process” (GO:0043065) were over-represented (Figure 1B). Other pathways significantly over-represented belonged to biological processes related to mRNA maturation and translation, in utero embryonic development and cytoskeleton organization (Figure 1B). Furthermore, RNA-seq data revealed that 34 and 117 genes were exclusively expressed in the <35 and ≥35 years age groups, respectively (Tables S2 and S3). “G-protein coupled receptor signaling pathway” (GO: 0007186) was the most frequently represented biological process in both groups (Figure 1C,D). The GO:0007186 pathway includes five genes (*OR4C13*, *OR52K2*, *OR10J3, OR10V1* and *TRHR*) and ten genes (*ADRA2C*, *CCL11*, *GPR55*, *OR4K15*, *OR6P1*, *OR8D4*, *OR51S1*, *OR56A5*, *TAAR5* and *TAS2R16*) in the <35 and ≥35 age groups, respectively. It is worth noting that there is no overlap between the two age groups. 

Recent data have highlighted the contribution of the cohesin complex in the correct chromosome segregation of meiotic chromosomes [21,22]. As no biological process related to sister chromatid cohesion was identified through GO analysis in the oocyte transcriptome, we performed a manual examination of DEGs searching for cohesin genes. Seven genes, *ESCO1*, *ESCO2*, *ESPL1*, *MAU2*, *SMC1A*, *SMC1B* and *STAG3*, were identified as belonged to the cohesin pathway. Their fold change ranged from −1 to +4. We found that the level of *ESCO1*, *ESCO2, ESPL1*, *SMC1A* and *STAG3* genes decreased in the ≥35 years age group, whereas both *SMC1B* and *MAU2* increased, though their differences were not statistically significant (Figure 1E).

Finally, the genome integrity of the 20 oocytes was analyzed by array comparative genomic hybridization (aCGH). We detected the loss of the X chromosome in two oocytes derived from two subjects belonging to the ≥35 years age group (Figure 1F). 

### 2.2. Interplay between Oocytes and Surrounding CCs

**Figure 2:**
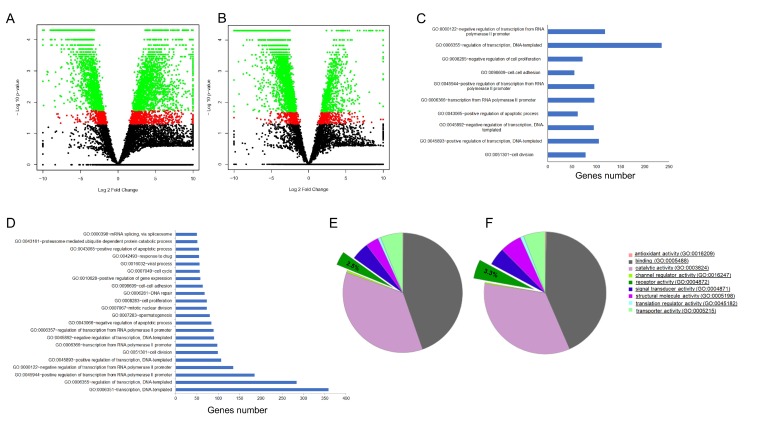
Interplay between oocyte and the surrounding CCs. (**A**) Volcano plot of gene expression changes in oocytes vs. CCs <35 years. (**B**) Volcano plot of gene expression changes in oocytes vs. CCs ≥35 years. (**C**) GO term enrichment analysis of biological processes that were significantly over-represented in oocytes vs. CCs <35 years. The pathways with the greatest number of annotated genes were related to gene transcription regulation. (**D**) GO term enrichment analysis of biological processes that were significantly over-represented in oocytes vs. CCs ≥35 years. The pathways with the greatest number of annotated genes were related to gene transcription regulation, mitotic nuclear division, cell cycle, and DNA repair. (**E**) Classification by molecular function of DEGs in oocytes <35 years according to PANTHER GO-slim. A total of 2.5% of DEGs (twenty genes) coded for receptors and were significantly more frequently expressed in oocytes <35 years. (**F**) Classification by molecular function of DEGs in oocytes ≥35 years according to PANTHER GO-slim. A total of 3.3% of DEGs (twenty-seven genes) encoded for receptors and were significantly more frequently expressed in oocytes from the ≥35 years age group.

Next, we performed the RNA-seq of CCs. We found no significant difference in gene expression between CCs derived from <35 and ≥35-year-old females (data not shown). Instead, quantitative comparison of gene levels between single oocytes and the corresponding CCs revealed thousands of DEGs (Figure 2A,B and Tables S4 and S5).

GO enrichment analysis revealed many pathways in oocytes vs. CCs in the <35 and ≥35 years groups. The biological function with the greatest number of annotated genes was related to gene transcription regulation (GO:0000122, GO:0006355, GO:0045944, GO:0006366; GO:0045892, GO:0045893). In addition, other pathways included DNA repair (GO:0006281), mitotic nuclear division (GO:0007067) and cell cycle (GO:0007049) (Figure 2C,D).

**Figure 3:**
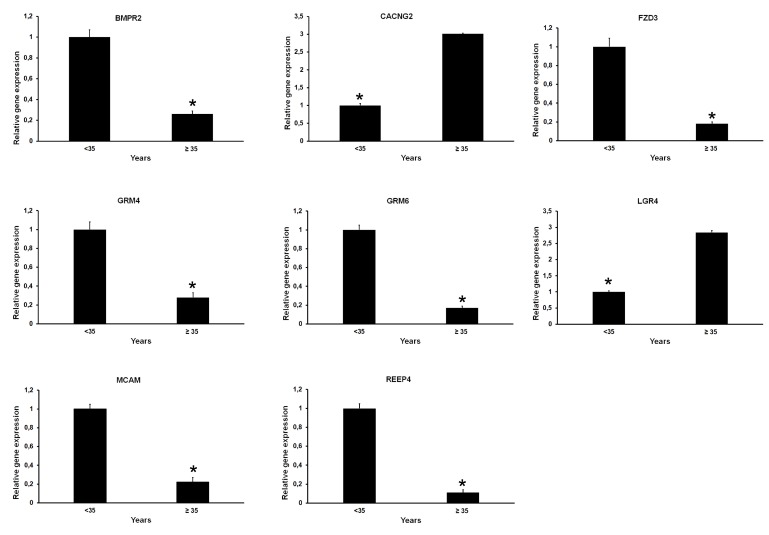
RNA-seq data validation. A subset of seven genes, *BMPR2*, *CACNG2, FZD3*, *GRM4*, *GRM6*, *LGR4* and *REEP4*, was validated in three technical replicates by RT-qPCR. These genes were chosen because they showed differential expression between oocytes <35 vs. ≥35 years. In addition, they are involved in important pathways for oocyte development and function (see Result section). Error bars represent standard deviation. * *p* < 0.05.

Oocytes cannot grow isolated from their surrounding CCs, although limited development is possible when in co-culture with soluble factors from CCs [31]. The signalling between the oocyte and the corresponding CCs is mediated by gap junction channels and receptors on oocyte membranes [32]. We therefore used “PANTHER GO-slim” for the classification by molecular function and biological process of both exclusively expressed genes and DEGs, with particular attention to receptor activity (GO:0004872). The analysis of genes exclusively expressed in oocytes from the <35 and ≥35 years age groups allowed us to identify 3 and 11 genes coding for receptors respectively. All (3/3 in oocytes <35 years) or most of them (7/11 in oocytes ≥35 years) are members of the G protein-coupled receptor superfamily (GPCR, Tables S6 and S7). Next, we focused on DEGs. Twenty genes (2.5% of DEGs) coding for receptors were significantly more expressed in oocytes <35 years (Figure 2E, Table S8). Though many genes belong to the GPCR superfamily, other genes, such as *FZD3*, *BMPR2*, *MCAM*, *GRM4*, *GRM6* and *REEP4*, play a highly specialized role. *FZD3* encodes a receptor for Wnt proteins. *BMPR2* codes a receptor for the TGF-β superfamily. *MCAM* encodes a surface receptor that triggers a transient increase in the intracellular calcium concentration. *GRM4* and *GRM6* are linked to the inhibition of the cyclic AMP cascade, whereas *REEP4* encodes a microtubule-binding protein required to ensure proper cell division. Twenty-seven genes (3.3% of DEGs) coding for receptors were significantly more expressed in oocytes from the ≥35 years group (Figure 2F and Table S9). Again, many are members of the GPCR superfamily. Of note, LGR4 is a receptor that activates the Wnt pathway, whereas CACNG2 regulates the trafficking and gating properties of AMPA-selective glutamate receptors (AMPARs). RNA-seq data was validated by quantitative RT-qPCR (Figure 3) and Pearson correlation analysis showed that RNA-seq and RT-qPCR data were significantly associated (*r* = 0.94).

**Figure 4:**
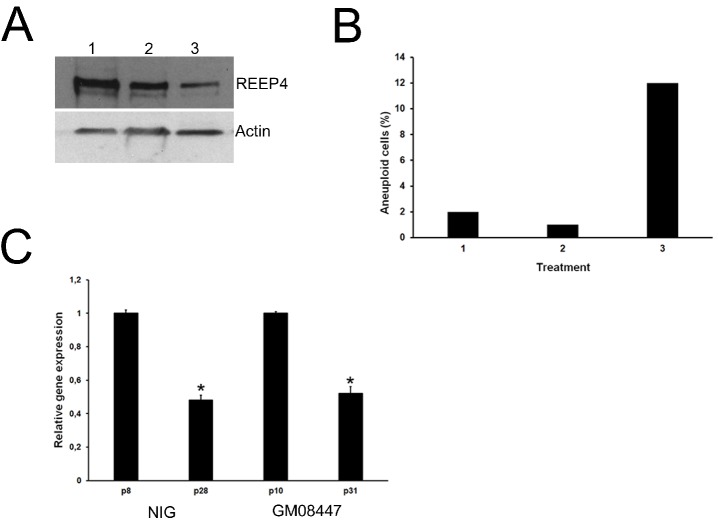
Effect of *REEP4* depletion in human primary fibroblasts. (**A**) Western blotting showing the downregulation of *REEP4* in human fibroblasts treated with 20 nM of smart pool siRNA (3) when compared with untreated (1) and mock siRNA-treated cells (2). (**B**) *REEP4* silencing (3) led to high frequency of aneuploidy cells when compared with untreated (1) and mock siRNA-treated cells. (**C**) The expression of *REEP4* was analyzed in three independent biological replicates of NIG (young and old) and GM08447 (young and old) cell lines. It was higher in young (p8 and p10 for NIG and GM08447, respectively) than in old fibroblasts (p28 and p31 for NIG and GM08447, respectively). Error bars represent standard deviation. * *p* < 0.05.

To investigate the effect of *REEP4* down-regulation (as occurs in oocytes ≥35 years), primary normal human fibroblasts were treated with RNAi against *REEP4* and its effect was evaluated 48 h after the treatment. Western blots showed that a specific inhibition of REEP4 synthesis was obtained. No decrease in REEP4 expression was observed with negative control RNAi (Figure 4A).

In addition, *REEP4* silencing induced chromosome aneuploidy with a frequency of 12% (Figure 4B). In order to investigate the expression of *REEP4* during in vitro aging, we used two fibroblast cell lines, NIG (male) and GM08447 (female) at different passages of cell culture progression. At an early passage, RT-qPCR results showed that both cell lines displayed high levels of *REEP4*, whereas its expression decreased during in vitro progression (Figure 4C).

## 3. Discussion

To date, there are very few data on the human oocyte transcriptome, with most obtained from older women or patients undergoing assisted reproduction [33,34]; only rarely has it been possible to analyze fresh oocytes (MII). In order to obtain new insight into the role of age in oocyte quality and the presence of meiotic error, we performed our analysis in fresh oocytes (MII derived from 20 women subdivided into two age groups). This selection permits us to detect a considerable difference in gene expression between the young (<35 years) and the older (≥35 years) age groups. In fact, we found that 1852 genes were dysregulated. However, since it has been shown that oocyte transcripts display changes in polyadenylation [35,36,37], we cannot exclude that some transcripts have been missed using our approach. Interestingly, several of the genes showing altered expression belong to cellular processes such as regulation of gene transcription, control of the apoptotic process, cytoskeleton organization and pathways related to mRNA maturation and translation. Previous studies performed in mature MII oocytes that did not fertilize after IVF-ICSI identified biochemical pathways very similar to those reported in this study [18,24,38]. Our data further support the notion that the gene expression profile changes with aged oocytes. The finding that pathways important for oocyte survival are affected allows us to hypothesize that these transcripts could be potential markers of oocyte quality. Further investigation will clarify this aspect.

In addition to DEGs, we found that young and older oocytes differ regarding the expression of genes uniquely expressed in each sample group. GO enrichment analysis shows that genes related to the GPCR pathway were significantly over-represented. It is worth noting that we identified the same pathway when transcriptome data was analyzed by the “PANTHER GO-slim” for the classification by molecular function with specific interest in the receptor activity. GPCRs are the largest family of cell-surface receptors, playing a pivotal role in membrane-initiated signaling processes, including immune responses, cardiac function, neurotransmission and metabolism. Beyond somatic cells, GPCRs are also expressed by human, rodent, *Xenopus* and Huoyan goose oocytes [39,40,41,42]. GPCRs are required to maintain high levels of cAMP in the oocytes and to prevent oocyte maturation. The role of GPCRs is further supported by the observation that oocytes defective for GPCRs display leaky meiotic arrest and premature re-entry into the cell cycle [43,44]. Our transcriptome profiles confirm that GPCRs are expressed in human oocytes but pinpoint that they are also dysregulated in young and old oocytes, providing a molecular marker of oocyte quality associated with age.

The interaction of an oocyte with its CCs is important for oocyte development and CCs provide environmental signals and metabolic support [45]. We found no significant difference in transcriptome profiles of CCs derived from young and old females. This observation indicates that likely gross alterations in gene expression of CCs are not tolerated, highlighting the importance of CCs for oocyte development. This notion is supported by the finding that CCs provide nutritional and metabolic support for the oocyte and intimate association with CCs is essential for oocyte survival and development [4,45]. This does not mean that poor quality of oocytes is tolerated more than poor quality of CCs. In fact, we cannot exclude that our results are biased because the oocyte transcriptome came from a single cell, instead of the averaged expression of many CCs, or because transcription in CCs is much more dynamic than the oocyte. The peculiar structural organization of CCs around the oocyte favours their continuous interaction. In fact, oocyte-secreted factors (OSFs) contribute to promoting the proliferation and differentiation of CCs and oocyte maturation and their transcription is influenced by age [46]. The interplay between oocyte and the surrounding CCs is mediated by oocyte membrane receptors. In addition to GPCR members, we identified several dysregulated genes coding for receptors. For example, *FZD3* and *LGR4* are involved in the activation of the Wnt/β-catenin pathway implicated in ovarian development, oogenesis, and early embryonic development [47,48]. Recently, it has been shown that *FZD3* and *LGR4* play a role in regulating follicular development and oocyte maturation [49,50].

Meiotic cohesin has a pivotal role in correct segregation of chromosomes in meiosis [51,52,53]. We found that *ESCO1*, *ESCO2, ESPL1*, *SMC1A* and *STAG3* genes decreased in oocytes ≥35 years though with no significant differences. In addition, RNA-seq data showed that the *REEP4* gene is down-regulated in oocytes ≥35 years. *REEP4* encodes an endoplasmic reticulum (ER) protein essential for sequestering nuclear envelope components away from chromatin, thereby contributing to proper cell division [54]. We showed that the silencing of *REEP4* caused chromosome aneuploidy in human primary fibroblasts and its expression decreased during in vitro aging. These findings are very interesting because among the 20 oocytes we identified chromosome imbalance, the loss of an X chromosome, in two oocytes ≥35 years, with an aneuploidy rate of 18%. The aneuploidy rate in oocytes shows great variation, from 2.8%, 18% in our study, to 65% [55]. These differences may be due to several factors including the number of oocytes analyzed, the genetic background of the donors and the type of cell analyzed. We speculate that *REEP4* function is maintained in both meiotic and mitotic cells and that the detrimental REEP4 in older oocytes interferes with the clearing of organelle membrane from chromosomes, leading to chromosome aneuploidy. The loss of an X chromosome is associated with Turner syndrome (45, X), the only viable monosomy in humans. In addition, the loss of X chromosome is the most frequent missegregation event in humans. In fact, its loss occurs in about 8% of human spontaneous abortions and this frequency remains constant over time [56,57].

This observation suggests that specific chromosome abnormalities could be associated with the alteration in mRNA transcripts. We found that detrimental *REEP4* leads to chromosome imbalance in human primary fibroblasts. This finding allows us to hypothesize that *REEP4* could also play a role in correct meiotic chromosome segregation and further studies will address this issue.

However, we cannot exclude that small changes in meiotic cohesin gene expression may contribute to aneuploidy. Taken together, our data show significant differences between the young and old oocytes in transcriptional profiles of genes involved in the central biological functions of oocytes. Furthermore, we identify a new putative mechanism responsible for chromosome imbalance in old oocytes suggesting a molecular basis for the increase in age-associated aneuploidy.

## 4. Materials and Methods

### 4.1. Study Population and Ethical Approval

Twenty healthy women undergoing in vitro fertilization (IVF) treatment for tubal or male factors were enrolled in this study. They were 19–42 years old with an antral follicular count (AFC) 12.2 ± 4.7 and 12 ± 4.5 and a body mass index (BMI) 21.6 ± 2.4 and 23.3 ± 4 for <35 and ≥35 years old, respectively (Table S10). All oocyte donors were Caucasian, non-smokers and had no evidence of systemic or reproductive medical conditions. In addition, they tested negative for HIV, both hepatitis C and B and sexually transmitted diseases. All subjects undergoing a hormone-stimulated cycle were administered a gonadotrophin releasing hormone (GnRH) agonist (Enantone; Takeda, Tokyo, Japan). Ovarian stimulation was performed by administering recombinant FSH (Gonal-F; Merck) alone or in combination with human menopausal gonadotrophin (HMG) (Menopur; Ferring) or recombinant LH (Luveris, Merck) at a dose of 112.5–300 IU daily, and ovulation was induced with 250 mg/0.5 mL recombinant human chorionic gonadotrophin (rHCG) (Ovitrelle; Merck).Thirty-six hours after rHCG administration, oocyte pick-up was performed and obtained COC were stored in IVF medium (Vitrolife) prior to denudation. CCs were removed from donated COC by fine needle cutting and corresponding oocytes were enzymatically denuded by brief exposure to 80 IU/mL hyaluronidase (Vitrolife), followed by mechanical denudation. The zona pellucida was also removed by brief exposure to a solution of 60 IU/mL Pronase (Sigma) in PBS. CCs and oocytes were collected in 2–3 μL of PBS and individually stored at −20 °C.

Evaluation of both oocyte and COC quality was based on their morphological appearance. In particular, as previously suggested [58], for the oocytes the following parameters were considered by light microscopy; rounded, regular shape; clear, moderately granular cytoplasm; a narrow perivitelline space; and an intact, colorless zona pellucida. Using these criteria, 20 MII oocytes and the surrounding CCs were included in this study in the period between May 2016 and June 2017. Oocytes and CCs were divided into two experimental groups based on the women’s age: <35 years (9 oocytes) and ≥35 years (11 oocytes).

Ethical approval for the study was obtained by the local Ethical Committee CEAVNO. All women included in this study gave informed consent.

### 4.2. Cell Culture

Normal human primary fibroblasts, named NIG, derived from a Caucasian boy as described previously [59], were used for investigating the effect of *REEP4* silencing on chromosome number. In addition, the GM08447 fibroblast cell line (purchased from Coriell Institute, Camden, NJ, USA), established from a young Caucasian woman, was used to study the expression of the *REEP4* gene during in vitro aging.

Cells were grown in Dulbecco’s minimal essential medium supplemented with 100 U/mL penicillin, 0.1 mg/mL streptomycin and 1% l-glutamine and 10% fetal calf serum in a humidified 5% CO_2_ atmosphere.

### 4.3. siRNA Treatment and Cytogenetic Analysis

Smart pool siRNA against *REEP4* and mock siRNA were purchased from Dharmacon (GE Healthcare, Lafayette, CO, USA). siRNA treatment and cytogenetic analysis were performed as previously described [60,61,62]. Human primary fibroblasts (at 40–60% confluence) were transfected with 20 nM *REEP4* siRNA by using Oligofectamine Reagent (Invitrogen Corporation, Carlsbad, CA, USA). Cells were analysed for aneuploidy 48 h post-transfection. Colcemid was added to the cultures for 90 min, followed by a 20-min incubation in 0.075 M KCl at 37 °C. One hundred metaphases were analysed. Chromosome aneuploidies were visualized by staining slides in Giemsa stain and detected by direct microscope visualization.

### 4.4. Western Blotting

Whole protein extracts from human fibroblast cells were resuspended with lysis buffer and protein concentration was estimated by the Bradford Protein Assay (Thermo Fisher Scientific, Waltham, MA, USA). Proteins, 60 mg per lane, were separated by SDS-PAGE, transferred to nitrocellulose membranes (Amersham) and incubated with the REEP4 (Abcam, Cambridge, UK) primary antibody. After removal of the unbound primary antibody, the membrane was incubated with secondary antibody-peroxidase conjugate (Sigma-Aldrich, Saint Louis, MO, USA), processed for detection by chemiluminescence (Amersham, GE Healthcare, Lafayette, CO, USA) and imaged on Biomax film (Sigma-Aldrich). Actin antibody was used as internal control.

### 4.5. Isolation of both DNA and RNA from Oocytes

Extraction of both DNA and RNA from single oocytes were achieved with the use of Nucleospin kit (Macherey-Nagel, Dure, Germany), according to the manufacturer’s instruction. Nucleic acid integrity and concentration were assessed using a Genova Nano spectrophotometer.

### 4.6. RNA Sequencing

RNA sequencing (RNA-seq) was performed on twenty oocytes and the corresponding twenty CCs as previously described [60,63,64] with minor modifications. A single library was produced for each sample. In this way, we generated twenty libraries for both oocytes and CCs. Libraries were obtained using the Ovation SoLo RNA-seq system (NuGEN, Redwood, CA, USA). The poly-A mRNA was fragmented and every purification step was performed using 1X Agencourt AMPure XP beads. Libraries were processed with Illumina cBot for cluster generation on the flowcell and sequenced on single-end mode on HiSeq 2500 (Illumina, Cambridge, UK). The CASAVA 1.8.2 version of the Illumina pipeline was used to process raw data for both format conversion and de-multiplexing. In order to perform the analysis of differentially expressed genes, the quality-checked reads were processed by the TopHat version 2.0.0 package (Bowtie 2 version 2.2.0) as FASTQ files [65,66,67]. All samples have a sequencing quality average Q30 ≥ 93%. The number of reads per sample are shown in Table S11. 

Reads were mapped to the human reference genome GRCh37/hg19. Only protein-coding genes were considered and gene level expression values were determined by read per kilobase of exon per million fragments mapped (RPKM). All genes with RPKM > 1 were designated as expressed. To reduce computation time without affecting the ability of detecting differentially expressed genes, we removed genes with very low expression that we arbitrarily fixed at ≤1 RPKM from the analysis. The power for detecting differential expression in genes with less than 1 RPKM is very low. The fold discovery rate (FDR) was calculated according to the Benjamini–Hochberg procedure as follows: (a) for a given α find the largest k such that P(k) ≤ (k/m)α; (b) All H(i) for *i* = 1,...,*k* were considered significant [68]. RNA-seq data are available on NCBI (SRP156824) DataSets.

### 4.7. Pathway Analysis and Function

Genes differentially expressed in oocytes were analyzed for biochemical pathways using Database for Annotation, Visualization and Integrated Discovery (DAVID) v6.8 (https://david.ncifcrf.gov).

The Panther database was used for gene classification by molecular function, biological process and cellular component (http://www.pantherdb.org).

### 4.8. cDNA Synthesis and Quantitative Real-Time PCR (RT-qPCR)

RNA-seq data was validated by RT-qPCR. cDNA was synthesized with SuperScript^TM^ II reverse transcriptase using oligo dT (Thermo Fisher Scientific, Waltham, MA, USA). PCR analyses were performed using Rotor Gene 3000 (Corbett). We pooled cDNA from subjects aged <35 and ≥35 years and RT-qPCR solutions were prepared using the same amount of cDNA for each sample. Reactions were run in triplicate and normalized with respect to HPRT. Primers used for mRNA expression analysis are listed in Table S12. 

### 4.9. Array Comparative Genomics Hybridization

Array comparative genomics hybridization (aCGH) was performed as recommended by the Agilent Agilent Oligonucleotide Array-Based CGH for Single Cell Analysis Enzymatic Labeling (Revision A1, August 2015) protocol for single cell analysis with slight modifications.

Briefly, the DNA extracted from the oocytes for each 8x array slide was dried in a lyophilizer and re-suspended in 2.5 µL of PBS. The DNA was amplified by the Rubicon PicoPlex WGA kit. Next, the protocol for DNA amplification was followed as recommended by Agilent.

In parallel, eight control reference female DNAs from Agilent (included in the kit SureTag Complete DNA Labeling Kit) for each array slide were amplified with the same Rubicon WGA kit as recommended by Agilent’s protocol. The eight amplified reference DNAs were pooled. The total amplified reference DNA was sufficient for up to 42 hybridization chambers.

Amplified oocyte and control reference DNAs were then labeled by the SureTag Complete DNA Labeling Kit as recommended by Agilent’s protocol. Labeled DNAs were purified by the columns included in the kit and then dried with a lyophilizer. Labeled DNAs were re-suspended in TE according to the protocol and yield and specific activity were assessed by the nanodrop ND1000 instrument (Thermo Scientific). Each oocyte labeled DNA was combined with the same volume of a labeled control reference DNA replicate. Hybridization mixture with Human Cot-1 DNA (Agilent), blocking solution (provided by the labeling kit) and 2× hybridization solution (Agilent) were prepared and added to the DNA mixture for each sample.

Hybridization mixtures were applied to a SurePrint G3 Human CGH Bundle, 8 × 60 K slide (Agilent) according to included manufacturer’s instructions, and hybridization was performed in a suitable rotating hoven for 16 h, 67 °C at 20 rpm. Slides were washed according to manufacturer’s instructions and slide images were acquired with Agilent’s microarray scanner G2565CA. Features were extracted using Agilent Feature extraction software version 12.0.2.2. As expected, amplified DNAs produce high levels of background/noise, so molecular karyotype and percentage of mosaicism were reconstructed by the use of Agilent’s Genomic Workbench software version 7.0.4.0, with parameters and algorithms set as recommended in our previous studies [69,70,71] with the only difference of two parameters: centralization = off and Fuzzy zero = off. This was chosen due to the nature itself of the experiment in which a low number of chromosomal changes and variable percentage of mosaics are expected; the centralization algorithm is used only in the case of an expected high number of chromosome imbalances and the fuzzy zero algorithm only in the case of an expected high percentage of mosaicism. Assembly (GRCh37/hg19) was used as the reference human map.

## Supplementary Materials

Supplementary Materials can be found at https://www.mdpi.com/1422-0067/21/6/1934/s1.

## Abbreviations

aCGH	Array Comparative Genomics Hybridization
BMI	Body mass index
cAMP	cyclic Adenosine monophosphate
CCs	cumulus cells
COC	Cumulus oophorus cell complex
DAVID	Annotation, Visualization and Integrated Discovery
DEGs	Differentially expressed genes
ER	Endoplasmic reticulum
FDR	Fold discovery rate
GnRH	Gonadotrophin releasing hormone
HMG	Human menopausal gonadotrophin
IVF	In vitro fertilization
OSFs	Oocyte-secreted factors
rHCG	Recombinant human chorionic gonadotrophin
RPKM	Read per kilobase of exon per million fragments mapped
RT-qPCR	Quantitative real-time PCR
SMC	Structural Maintenance of Chromosomes
