# Supplementary material for: Chromosome Missegregation in Single Human Oocytes Is Related to the Age and Gene Expression Profile
Source: Int J Mol Sci. 2020 Mar 12;21(6):1934. doi: 10.3390/ijms21061934 (PMC7139522; doi:10.3390/ijms21061934)
Supplement: Supplementary file 1 [file ijms-21-01934-s001.pdf]

Supplementary Table 1. Dysregulated genes in oocytes > 35 vs ≥ 35 years

Gene	Log2(Fold_change)
AGO4	-7,97374
AAMP	-0,44886
AARS	-2,71138
ABCB10	7,73909
ABCC4	2,84545
ABLIM1	-3,87124
ACAD8	-4,66338
ACD	-4,21424
ACOX1	5,00936
ACTB	3,13633
ACTR1A	3,27874
ADAM15	-3,80009
ADAMTS17	3,8264
ADAMTS2	5,42756
ADAMTSL5	-4,41511
ADAT1	-2,37232
ADCY3	4,31861
ADPRM	-3,83527
ADRA2B	-4,51954
ADRBK2	2,96731
AFAP1L2	-2,61539
AFG3L2	6,77583
AGFG2	-2,47869
AGMO	2,44045
AGPAT6	2,45042
AHNAK	5,19347
AIFM1	-5,50938
AIM1	2,34332
AK5	-4,89849
AKAP1	-2,35969
AKAP11	-2,53262
AKAP6	-2,59241
AKIRIN1	3,30715

AKR1B10	4,22712
ALAD	3,38698
ALDH18A1	5,05627
ALDH1A2	3,72932
ALDH1B1	-3,87535
ALDH6A1	3,13812
ALDOA	4,06089
ALKBH2	-2,91435
ALKBH5	-2,04949
ALOX15	-4,35058
AMER1	6,81849
AMIGO1	4,08725
ANAPC16	2,21896
ANKH	-2,41959
ANKRD11	-2,41624
ANKRD13C	-6,12993
ANKRD20A9P	2,17935
ANKRD26P1	-3,57465
ANKRD27	2,71401
ANKRD42	-4,60589
ANKRD45	3,45211
ANKS1A	1,8768
ANO5	5,08031
ANXA2	6,72055
AP3S2	3,16727
APBA1	-3,34168
APPL2	2,17536
AQP6	3,83113
AQP7P1	5,67681
ARF5	-8,17323
ARG2	-2,53982
ARGFX	3,53624
ARHGAP1	2,34698
ARHGAP18	3,31486
ARHGAP19,ARHGAP19-SLIT1	-3,75279
ARHGAP20	4,98723

ARHGAP31	5,60134
ARHGAP40	6,00191
ARHGAP42	-2,27685
ARHGDIA	3,08655
ARHGEF10	5,06466
ARHGEF6	2,72985
ARID3B	-2,56855
ARIH1	-2,04216
ARL2BP	2,34775
ARL8B	-2,60527
ARPP19	2,54696
ARRDC4	4,85559
ARV1	-2,34711
ASAP2	-3,2874
ASB6	-4,52708
ASF1B	3,39149
ASPH	4,51542
ASPHD1	-4,47277
ASTL	1,81437
ASTN2	2,42832
ASUN	0,02324
ATAD3C	-2,42914
ATF1	-9,14184
ATF6	2,08756
ATG2B	2,18167
ATIC	2,74707
ATMIN	-2,67013
ATP10B	-5,24786
ATP10D	3,64832
ATP11B	-2,12731
ATP13A1	2,58856
ATP1B3	-3,35139
ATP5F1	-4,44556
ATP6AP2	3,54298
ATP6V0A4	6,57705
ATP6V1C1	-2,36094
ATP6V1G1	-2,67434

ATP7A	5,55686
ATPAF2	-2,08119
ATRN	5,03211
ATXN3	4,27278
ATXN7L2	-5,27082
AXIN2	-2,46269
AZIN1	-2,64802
B2M	3,7269
B3GALNT2	3,08654
B3GNT4	-2,09317
B3GNT6	4,19641
B3GNT7	-4,91346
BAG3	1,32592
BAG4	3,09828
BAIAP2L2	-2,67016
BAK1	2,08327
BANP	-0,33057
BARX2	-2,5201
BATF2	-5,57949
BBS5	2,88724
BCAS2	2,08338
BCL2L1	-5,33755
BCL2L11	7,16414
BCL2L15	2,98425
BCRP2	-4,00947
BEND3P3	-3,66419
BLOC1S2	3,11742
BMPR2	-2,07557
BMS1P20	4,29288
BPNT1	5,07499
BRK1	6,54081
BRS3	-0,10433
BTBD2	-4,05602
BTBD7	-4,17756
BYSL	-4,75944
C10orf12	3,01966
C10orf2	-7,27702

C10orf32,C10orf32-ASMT	3,04025
C10orf76	3,68912
C11orf84	-0,37844
C12orf49	5,46069
C12orf75	3,01021
C14orf1	2,60578
C14orf2	-3,07824
C15orf39	1,91093
C16orf87	4,11966
C17orf103	3,25043
C17orf51	3,41053
C18orf25	-3,21362
C19orf24	-4,37375
C19orf57	-6,17988
C1orf146	4,13564
C1orf21	-3,31541
C1orf220	9,70186
C1orf226	-0,27814
C1orf233	-4,81991
C2CD5	-2,4769
C3orf70	1,98666
C4orf19	3,1057
C5orf27	3,581
C5orf28	4,16642
C5orf49	2,08269
C5orf55	2,40936
C5orf64	3,65564
C6orf99	9,26506
C7orf26	2,38443
C8orf33	4,80106
C9orf40	-5,20448
C9orf41	-4,29381
CA5BP1	2,72347
CABYR	-2,95828
CACNA1C-IT2	8,4145
CACNA1H	-4,68349
CACNG2	6,19521

CACNG7	-3,699
CALB1	-2,52932
CALML4	-2,04464
CALU	3,57689
CAMK1D	-3,8294
CAMK2N1	4,68777
CAMSAP1	-3,29447
CAND1	-3,06362
CAPN3	5,84781
CARD6	-3,34501
CAV2	4,95059
CBLB	5,51896
CBR4	2,74156
CBX8	6,01608
CC2D1A	3,15651
CCAR1	-4,251
CCDC101	-2,05675
CCDC106	3,01504
CCDC130	2,28052
CCDC144B	3,68932
CCDC159	-3,3168
CCDC171	-3,31961
CCDC174	2,65649
CCDC177	-3,30405
CCDC43	-5,10119
CCDC58	2,96209
CCDC87	-3,42408
CCDC88A	-2,52383
CCDC9	-6,36409
CCDC92	4,07536
CCL28	9,2867
CCNG1	-3,06017
CCNT1	-3,43253
CCNT2	-2,73688
CCP110	2,24812
CCRN4L	8,95825
CD248	2,61087

CD82	3,73069
CDC20	2,87453
CDC25B	-2,56632
CDC42	-2,08746
CDC42EP4	2,71274
CDCA4	-4,02687
CDCA8	5,26469
CDH3	-2,53361
CDH9	-5,89485
CDK5R2	-2,52403
CDKL1	3,35985
CDKL2	-3,16053
CDT1	3,19246
CELF1	-4,51585
CELF2	-4,93641
CENPH	-2,49978
CEP128	2,13404
CEP152	2,19682
CEP170B	-3,25218
CFL1	-2,50043
CHAF1A	-1,9964
CHAMP1	-2,86286
CHCHD2	-2,37147
CHD7	-3,44844
CHD9	8,30692
CHEK2P2	-3,39501
CHERP	-2,64404
CHMP2B	3,41958
CHMP4B	-3,8842
CHMP7	-2,83173
CHST10	-2,68602
CHST6	2,49371
CIART	-2,20284
CIPC	2,16129
CIRBP	-3,318
CKAP4	-2,13557
CLCN5	-2,40571

CLDND1	2,09854
CLEC2D	4,1219
CLEC4D	5,5473
CLN6	-3,15666
CLPX	-3,35389
CLSTN3	-2,29578
CMBL	3,1061
CMKLR1	-3,74722
CMTR1	-3,15679
CNBP	-2,21191
CNDP2	-3,28282
CNN1	2,89058
CNOT11	-2,0963
CNTD2	4,54794
CNTLN	2,55742
COA1	3,16041
COA7	-2,25917
COG3	4,10634
COIL	-4,65936
COL6A1	6,84192
COLEC10	9,51205
COLEC12	-5,69436
COMMD4	-5,86221
COMMD9	4,96794
CORO2A	2,97286
COX18	6,22676
COX4I1	-3,61414
COX5A	-2,01894
COX7A2L	-2,03641
CPLX4	-3,67351
CPSF4L	-7,16011
CPT2	4,3021
CRAMP1L	-2,67102
CREB1	-4,30737
CRIPT	2,65967
CRMP1	-3,1596
CRNKL1	3,44022

CRNN	6,43709
CROCC	1,26716
CRTAP	-4,26291
CRX	2,14586
CRY1	-1,99294
CRYZL1	-4,87294
CSE1L	-2,23528
CSF1R	-2,28585
CSTB	-2,41945
CSTF3	-5,22755
CTAGE5	-4,71197
CTBP1-AS2	5,6554
CTC1	3,4646
CTDSPL2	2,88459
CTNNB1	2,44194
CTNND1	-2,01568
CTRB1	-5,84467
CTRB2	-4,69151
CTSC	3,06598
CTSV	5,58988
CTTNBP2	-4,65152
CWC22	-2,67325
CXXC4	-2,99149
CYFIP2	-3,06435
CYLD	-2,62409
CYP1B1	-5,47775
CYP4A11	2,99508
CYTH3	-3,09167
DAD1	-4,01032
DCAF4L2	5,31958
DCBLD2	3,33065
DCC	1,89918
DCHS2	-3,39925
DCLRE1C	3,856
DCP1B	2,46874
DCTN5	-2,23852
DCUN1D5	-2,70624

DDIT4	4,34813
DDN	-1,24915
DDX18	-2,36272
DDX23	-2,77365
DDX50	-2,279
DDX55	-2,02301
DECR1	-2,11956
DEGS1	4,12382
DEK	-2,71009
DENND4B	-5,91513
DERL2	1,37434
DFFA	0,23543
DFFB	4,99707
DFNA5	5,89053
DGCR2	-6,63545
DGKB	-2,64911
DGKQ	1,371
DHTKD1	4,27599
DLD	-2,69729
DLG5	3,63349
DLGAP4	-4,16153
DLGAP5	3,4506
DMBT1P1	2,67672
DMD	-5,97064
DMRTA1	-3,59086
DMWD	-3,81586
DNAH10	-2,96081
DNAJB4	-2,7677
DNAJC1	-2,86266
DNAJC11	3,49341
DNAJC16	3,42459
DNAJC2	-3,38215
DNM1P46	-3,98101
DNM2	-4,5808
DOCK1	-3,63744
DOCK10	-3,72266
DPAGT1	4,86369

DPF2	4,54667
DPP9	-2,84255
DPPA5	1,87971
DPYSL3	5,31669
DSCC1	-3,57273
DSP	3,77408
DTWD2	-2,80277
DTX3L	3,55015
DUSP27	-5,70775
DUSP8	3,81053
DYNAP	4,57416
DYNC1H1	3,20664
DYNLL1	3,09181
DYSF	-4,96549
EBF1	-2,35764
EBP	2,419
EDAR	-5,80547
EEF1G	3,57288
EEPD1	-3,45262
EFNB2	2,91337
EFR3A	2,31578
EGLN3	4,31817
EHBP1	4,78142
EHD2	6,35532
EIF2S2	2,4269
EIF3E	-2,55218
EIF3I	2,95761
EIF3K	2,65487
EIF4G3	-3,63003
EIF5A	-2,78334
EIF5A2	4,54957
ELOF1	-2,98482
ELOVL2	-4,88546
ELP4	3,09352
ENKD1	-1,13777
ENPP1	-2,73038
ENPP4	-4,73993

ENTHD2	-3,64989
EP400NL	-4,25041
EPAS1	-2,52134
EPB41L4B	3,18646
EPB41L5	-2,69606
EPHA4	2,92257
EPHB1	2,27253
EPS15L1	-3,70748
EPT1	2,48572
ERAL1	-3,98647
ERAP2	6,84171
ERCC3	-3,93164
ERG	-7,08184
ERI1	3,39667
ERMP1	-3,07195
ERP44	-2,88133
ESRG	2,17512
ESYT3	3,7717
ETNK1	3,59202
EVI5	4,17595
EWSR1	-2,93113
EXD1	3,50064
EXOC3L2	3,8442
EXOC4	3,72521
EXOC6B	-2,67946
EXOC7	-5,23746
EXOSC6	3,37393
F11R	2,15378
F2R	3,70484
F3	4,88833
F5	4,33158
FABP5	6,25606
FADD	-5,54247
FAH	8,47878
FAHD2A	6,12657
FAM102A	4,0977
FAM106A	4,22544

FAM110C	-6,90365
FAM117A	3,14323
FAM118A	-2,60345
FAM126B	2,57407
FAM134A	3,05523
FAM154A	-4,58018
FAM161A	4,68528
FAM163B	-4,49411
FAM192A	3,56749
FAM193A	-2,30721
FAM199X	2,80767
FAM200A	-2,4754
FAM204A	3,83206
FAM227A	-2,03573
FAM228A	3,1739
FAM230B	2,794
FAM230C	7,77622
FAM41C	-2,08258
FAM46B	-2,66987
FAM53C	-3,50396
FAM71F2	2,5465
FAM76B	2,5073
FAM83B	-2,22392
FAM83E	-5,99875
FAM83H-AS1	2,93517
FAM96A	3,78852
FAM98B	4,01845
FANCF	3,6919
FANCL	-5,94098
FAR2P2	2,62414
FBL	2,96895
FBLIM1	3,29841
FBLN5	3,395
FBLN7	2,76806
FBXL18	3,33197
FBXO36	-3,95854
FBXO42	-3,04572

FBXO48	3,95899
FBXW12	-2,44606
FCF1	-3,92051
FCHSD2	3,34817
FDX1	3,93445
FEM1B	2,27554
FERMT2	-4,36789
FEZF2	-2,0231
FFAR4	5,03064
FGD3	-4,70759
FGD5	-2,32247
FGD5P1	6,63716
FGF5	2,89903
FH	2,08411
FIG4	-2,60719
FIGLA	3,76195
FKBP14	2,74524
FLCN	2,16906
FLJ30403	3,08369
FLJ31356	3,86645
FLJ31662	2,21704
FLJ36777	5,75701
FLJ42102	2,57917
FLJ43315	3,35158
FLJ44635	-2,01606
FLOT2	-4,01479
FLRT2	6,47347
FLT1	5,44636
FNBP4	-2,14149
FNDC4	-5,59132
FNTA	4,69976
FOLR4	-3,69473
FOSL1	-4,61197
FOXD4L5	-3,06178
FOXK2	-3,12829
FOXL2NB	4,15875
FOXQ1	-2,61356

FOXR1	2,50762
FOXRED2	-3,30256
FRMD4B	-2,38234
FRRS1L	3,61796
FRS3	-2,67135
FRYL	-3,31057
FTH1	2,7078
FTL	-2,91821
FUCA1	-2,09573
FUT4	-4,28531
FXR1	5,96264
FYCO1	-3,89655
FZD10-AS1	5,33614
FZD3	-2,33564
G3BP1	-3,69
G6PD	-6,13275
GABARAPL1	2,01713
GALNT15	2,80673
GALNT3	-2,35463
GAPDH	8,67235
GAREM	3,14161
GARS	-3,0564
GAS2L3	2,4204
GAS6-AS2	2,02687
GAS7	-3,59912
GATA6	4,39249
GATAD2A	-2,48106
GCA	3,81046
GCC2	-2,59801
GCLM	2,50687
GDI1	-3,05345
GFOD2	3,26161
GFRA1	2,71972
GGT8P	4,65856
GID8	-4,63411
GINS2	-3,61634
GJA1	2,63847

GLDN	2,16798
GLI3	-3,30525
GLO1	-4,58664
GLRX	2,39328
GLUD1	6,69148
GMPS	5,14389
GNA11	-5,08604
GNA12	5,71759
GNAO1	7,31018
GNAS	3,58988
GNB1	-2,16094
GNL2	-4,10083
GNL3	-4,12067
GNPDA1	-1,95221
GNPNAT1	3,86182
GOLGA4	-2,1432
GOLGA6C	5,29569
GOLGA6L6	2,50039
GOLIM4	-2,07808
GOSR1	2,43476
GOT2	-3,70395
GPATCH1	-2,00288
GPATCH8	5,2067
GPBP1	5,8696
GPC6	3,88966
GPKOW	3,03234
GPR133	2,1623
GPR143	2,59738
GPR160	-2,77865
GRAMD1B	4,48467
GRB7	-2,40085
GRM4	-2,7887
GRM6	-4,19039
GRN	5,30169
GRWD1	8,51421
GSPT1	-2,8049
GSTA1	3,98962

GSTA4	4,33564
GSTP1	4,11791
GSTTP2	3,00266
GTF2H2	3,13182
GTF3C3	-3,11919
GUCA1B	4,70369
GUSBP10	-8,5769
GZF1	2,64891
H2AFX	-3,16254
H6PD	4,38608
HABP2	-3,13969
HAUS5	-3,57923
HAUS8	-3,99295
HCAR3	-4,3172
HCN4	-2,72093
HDAC3	1,91274
HDGFL1	-3,33651
HDGFRP2	-3,20299
HDGFRP3	-5,84997
HDHD2	5,46185
HDHD3	-5,81794
HEATR5A	3,686
HECTD4	-2,64676
HELLS	-3,58631
HELQ	2,45223
HERC2P7	-2,52475
HEXIM1	-2,89525
HEYL	-1,87308
HGSNAT	2,80755
HHLA1	-2,04485
HIATL1	2,86013
HINT2	-4,16368
HIP1R	-2,31392
HIPK3	-2,6201
HIST1H1E	-4,19076
HIST1H2AG	-2,98706
HKR1	2,92806

HMG20A	-3,20614
HMG20B	-6,48044
HMGA1	-4,07623
HMGB3P1	3,94867
HMGN4	3,35636
HNRNPA0	-3,22876
HNRNPA1P33	3,76442
HNRNPA3	2,8141
HOMER2	-4,5477
HOOK3	2,40717
HOXA4	6,90624
HOXC9	8,9089
HPGDS	-4,20016
HSD17B12	3,60808
HSD17B7	6,38182
HSD3BP4	2,47279
HSDL1	3,83508
HSF5	1,84692
HSPA2	2,77066
HTR3C	2,91659
HTRA4	6,47263
HUS1	5,62389
HYOU1	3,18876
ICT1	-2,88743
IER2	-2,02574
IER3IP1	-2,76089
IER5	-4,01572
IER5L	-3,26372
IFIT5	-3,9274
IFNL4	-8,42398
IFT22	-3,22263
IGF1	7,0076
IGF2BP2	-4,64697
IGFBP1	-2,66896
IGFBP2	1,96842
IGFBP3	-3,75943
IGFBPL1	-2,33797

IKBKAP	6,97837
IL17C	-2,07337
IL6ST	4,23732
ILF3-AS1	4,68537
IMP4	-3,48882
IMPAD1	-3,17706
INADL	2,95378
ING5	-4,1663
INHA	4,06773
INIP	4,77306
INMT,INMT-FAM188B	3,81815
INPP5A	-3,55633
INSC	-4,3143
INSL4	-4,45029
INTS3	-2,75332
IQCD	-4,0443
IQCJ,IQCJ-SCHIP1	-3,39713
IQGAP3	2,9666
IQUB	4,24774
IRAK4	3,05009
IREB2	5,08268
IRF6	-3,75078
IRGM	-2,5001
ISPD	-5,12586
ITCH	-2,60634
ITFG2	6,90847
ITGA11	1,81665
ITGB5	-3,66452
ITIH2	2,25996
ITM2B	3,31586
JAGN1	-8,01094
JAK2	-3,08823
JAM2	2,8907
JHDM1D-AS1	2,03041
JMJD6	-5,33365
JOSD1	2,5523
JUND	2,27759

KAL1	2,13044
KATNB1	-2,24386
KBTBD6	-2,43084
KCNA7	4,31914
KCNC4	5,35136
KCNE3	-3,4055
KCNK18	-3,98579
KCNK5	-3,8262
KCNQ1OT1	1,92833
KCTD13	-3,56709
KDELR1	4,87613
KDM4A	3,04157
KDM4B	-3,39796
KDM5B	5,75448
KDSR	2,90165
KHDC3L	-2,4868
KHDRBS1	-2,68965
KHDRBS3	-2,90557
KIAA0020	2,75153
KIAA0087	6,10595
KIAA0430	5,53098
KIAA0556	-2,35467
KIAA0895L	5,51874
KIAA1024	-4,52775
KIAA1109	-2,23391
KIAA1210	3,84978
KIAA1462	8,84784
KIAA1524	-4,9136
KIAA1614	2,69711
KIAA1875	4,4811
KIAA1958	3,08041
KIF13B	-3,02421
KIF3B	-2,86078
KIF5B	-2,78571
KLC2	-6,5319
KLF12	2,63486
KLF16	-3,63986

KLF2	-4,8867
KLHDC10	-2,50806
KLHL20	2,03032
KLHL26	-2,46876
KLHL3	-4,69188
KLHL42	-6,06597
KLK4	-3,20969
KLKP1	5,45386
KMT2A	2,05513
KMT2B	-2,35167
KPNA5	-4,89566
KRT16	-2,80619
KRT5	-1,0732
KSR1	-2,07634
KSR2	-3,07114
KXD1	-5,19069
LAMA1	8,82266
LAMB2	8,9738
LAMP2	-2,31163
LARS	2,47758
LCOR	1,96609
LCP2	3,52839
LDHA	2,06109
LEAP2	3,75568
LEP	5,26448
LGR4	2,95436
LHFP	5,30551
LHFPL5	6,97235
LHX2	2,82798
LIN28A	2,86153
LINC-ROR	-2,74614
LINC00032	3,62591
LINC00162	-7,32329
LINC00263	2,22315
LINC00276	3,53276
LINC00284	-6,63487
LINC00342	-1,8918

LINC00346	3,60541
LINC00475	1,05103
LINC00488	-5,09285
LINC00506	2,00585
LINC00507	2,94843
LINC00514	2,47293
LINC00518	5,85585
LINC00547	3,47066
LINC00578	4,17681
LINC00637	3,75681
LINC00638	8,92287
LINC00657	5,83429
LINC00663	2,50966
LINC00670	3,97203
LINC00857	5,72763
LINC00887	3,66589
LINC00898	0,83966
LINC00910	2,43896
LINC00924	3,13397
LINC00926	2,64288
LINC00927	5,27312
LINC00958	2,14133
LINC00963	2,97638
LINC00964	2,57956
LINC00970	3,39107
LINC00996	3,2261
LINC00997	-2,83384
LINC01012	3,91556
LINC01030	-2,43111
LINC01087	2,82087
LINC01134	5,00869
LINC01158	7,24528
LINC01160	2,71413
LINC01204	4,00455
LINC01299	3,25842
LINC01344	-5,50781
LINC01356	2,12766

LINGO1	-2,11424
LIX1	-6,30206
LLPH	-3,24583
LMBRD1	-1,96207
LMO4	-5,5343
LMTK2	-2,01102
LNX2	2,62653
LOC100128554	1,14079
LOC100129935	8,48023
LOC100130451	3,13005
LOC100130899	4,65809
LOC100131257	2,52958
LOC100188947	3,19055
LOC100287015	2,67044
LOC100287792	2,43424
LOC100287846	5,06277
LOC100289019	3,87966
LOC100289495	2,69843
LOC100420587	2,66007
LOC100505817	-1,96152
LOC100506082	-2,63629
LOC100506422	-4,93164
LOC100506548	2,92035
LOC100506688	4,32173
LOC100506730	4,84183
LOC100506746	4,05496
LOC100506844	-2,39492
LOC100652824	-4,08559
LOC100996447	3,65336
LOC101926892	9,93512
LOC101926897	5,66952
LOC101926928	5,24622
LOC101926996	3,25367
LOC101927136	2,93226
LOC101927207	-1,0101
LOC101927280	4,19847
LOC101927421	2,77085

LOC101927623	7,17889
LOC101927797	3,35047
LOC101927865	-2,97911
LOC101928053	4,87394
LOC101928063	-2,68878
LOC101928104	5,86439
LOC101928303	3,33616
LOC101928401	3,18921
LOC101928495	-1,96931
LOC101928936	2,43639
LOC101928940	5,73316
LOC101929767	-4,32301
LOC102723510	-2,8105
LOC102723769	-2,17115
LOC102724316	3,99056
LOC102724927	3,64018
LOC143666	6,40819
LOC145837	-6,85675
LOC158435	4,83683
LOC283070	-3,64704
LOC283683	-3,18741
LOC284395	6,27998
LOC284412	3,59277
LOC284581	2,13705
LOC284632	-4,31012
LOC286437	3,47313
LOC339803	-1,96389
LOC389834	4,28237
LOC399715	5,63739
LOC440300	2,99805
LOC440600	-3,8371
LOC442132	2,70872
LOC613037	6,37901
LOC641515	4,79362
LOC642236	3,30913
LOC642852	-2,0566
LOC643201	7,17875

LOC643406	2,49655
LOC644669	4,63317
LOC644961	-4,86083
LOC645434	-2,83151
LOC645752	6,97512
LOC650226	4,67349
LOC728730	-4,10898
LOC728752	2,07632
LOC729987	4,96812
LOC730102	4,62265
LOC90784	-2,73477
LONP2	3,67983
LPCAT4	3,06006
LRAT	3,28905
LRIG1	-3,35391
LRRC1	-4,25196
LRRC37BP1	3,86063
LRRC57	2,12425
LRRC75A-AS1	4,02169
LRRN1	-2,55028
LRRN4	-4,29057
LSM12	-3,80021
M6PR	5,92931
MAB21L3	2,06159
MACC1	3,17813
MAD2L1	-3,54414
MAEA	-3,85347
MAF1	5,58182
MAFG	-3,61514
MAGEE2	7,02633
MAGI1	-2,40205
MAL2	2,63821
MALSU1	-5,61614
MAN2A2	-1,7705
MANF	-2,94656
MANSC1	-3,74442
MAP1LC3B2	4,03942

MAP1LC3C	-3,54651
MAP1S	4,26486
MAP2K6	2,62538
MAP2K7	-3,70622
MAP3K2	-2,29737
MAP6D1	4,83188
MAPRE3	-1,99355
MARCH5	-2,02039
MARS	-3,14424
MAST3	-3,00169
MASTL	5,36547
MBD1	4,58441
MBTD1	2,81742
MCAM	-5,70248
MCL1	4,63849
MCM4	-4,11579
MCOLN1	-4,81836
MCPH1	-4,42411
MCTP1	-4,16743
ME2	3,64299
MEAF6	-2,00582
MED16	3,88515
MED19	-1,92317
MED25	2,71575
METTL14	-2,09875
METTL21EP	-5,38219
METTL2A	3,89331
MEX3D	-3,26203
MFN1	-3,6798
MFSD6	6,87351
MGAT4A	3,4577
MGAT5B	-3,6492
MGC27345	2,65356
MGC72080	2,31225
MGEA5	-2,77498
MICAL3	-4,33274
MILR1	3,47939

MIR4461	2,15111
MIS12	-4,60865
MLLT4	-3,04344
MLYCD	-8,39987
MMD2	3,8622
MOB1A	1,97233
MON1B	4,78427
MON2	-2,26722
MOSPD1	-2,71224
MPP2	-4,68199
MRAS	3,44568
MRE11A	3,71886
MREG	2,09494
MRFAP1	-3,42145
MRGBP	-2,03224
MRGPRX3	2,35724
MRO	3,00237
MRPL13	-5,81028
MRPL18	-3,2766
MRPL37	2,18415
MRPL4	-4,60645
MRPL40	-2,93444
MRPS23	2,54218
MRPS30	-4,12888
MRPS31	-3,22572
MRPS5	-5,04122
MRTO4	-2,55724
MST1L	3,38973
MT1E	2,96661
MT2A	3,11635
MTCH2	-3,04472
MTERFD1	2,92171
MTG2	2,6057
MTL5	4,40048
MTMR1	-1,9094
MTMR12	-3,42516
MTMR4	3,22463

MTMR6	-2,65463
MTMR9	2,35808
MTRNR2L4	2,32133
MTRNR2L9	2,24251
MUC17	5,85598
MUC19	4,97019
MUC6	3,52334
MUL1	-3,03122
MYBBP1A	-3,95108
MYBL1	3,08609
MYBL2	-2,86596
MYCBP	-2,48219
MYH10	4,1781
MYL12A	3,38008
MYL6	3,31052
MYO18A	-2,96212
MYO5C	4,92454
MYO9B	-3,10968
MYOG	6,68963
MYSM1	5,53052
MYT1	4,51539
NAAA	2,33106
NAB1	-2,93018
NACC2	-3,16031
NADK2	4,63893
NANOG	3,09399
NAP1L1	-2,07105
NAP1L4	-3,76399
NAPG	1,94471
NARG2	5,96848
NARS	2,96325
NBAS	-3,44169
NBEAL1	-1,87482
NBN	3,75306
NCAPG2	3,86552
NCAPH	-1,7063
NCBP2-AS2	-3,98137

NCCRP1	5,11419
NCKAP5L	3,19178
NCOA7	5,45844
NCOR1	-2,53944
NDFIP1	3,05169
NDRG4	-5,29962
NDST2	-1,86159
NDUFA10	4,19352
NDUFA6	4,2685
NDUFA7	-2,66523
NDUFA9	4,28012
NDUFAB1	-3,1089
NDUFS6	1,38052
NEDD1	-2,94735
NEK1	-4,68215
NETO2	-1,98749
NEUROD1	2,15789
NFIL3	-5,81409
NFS1	6,11763
NFX1	5,50832
NKAP	-2,65092
NLK	-2,21784
NLRP5	-2,09708
NME5	-2,05483
NOC4L	5,25735
NOD2	3,90033
NOL7	-3,21686
NOL9	2,5942
NOP56	4,76558
NPC2	-3,10982
NPEPPS	-2,48648
NPM3	-2,80596
NPTX2	2,30431
NPTXR	5,29114
NR2C1	3,01141
NRAS	2,07711
NRP1	6,08759

NRSN1	-4,53861
NSMCE2	-5,25438
NSMCE4A	-2,89407
NSUN2	-3,11527
NUAK2	2,06442
NUCB2	2,10194
NUDC	-3,38314
NUFIP1	4,53665
NUP160	-2,14691
NWD2	-5,11817
OAF	-2,4999
OAS3	-5,55024
OCLN	2,7157
ODF2L	2,81
OGFOD1	-2,3449
OIP5-AS1	-2,5607
OLFML1	5,71308
OOEP	2,22535
OR1E2	9,25914
OR1N1	-4,29619
OR2AG2	-4,89381
OR2M3	-3,40911
OR6C68	-4,37667
OR7E37P	3,34169
OR7E91P	-4,06999
OR8G2	-4,80059
OSTC	3,81379
OTOGL	-4,96012
OTUD7A	-5,69413
OVCH2	-2,83744
PABPC1L	-2,54844
PACS1	2,16424
PACSIN2	5,8528
PAFAH2	-3,17064
PAG1	-2,86682
PALB2	-3,4194
PAPD4	2,82973

PAPOLA	-1,91422
PARM1	2,27219
PARN	2,26716
PARP12	-2,55401
PARP6	3,40913
PART1	5,72642
PARVA	4,42136
PATE1	6,81415
PBX1	-3,44539
PCDH11Y	4,01196
PCDHB9	3,19408
PCF11	-3,44172
PCIF1	-1,99802
PCYOX1	3,61469
PCYOX1L	-3,70996
PDCD10	-3,41328
PDCD4-AS1	7,5582
PDCD7	-3,38673
PDCL3	-2,29228
PDDC1	2,3938
PDE7B	2,02007
PDP1	-3,70413
PDP2	2,61621
PDPN	2,39474
PDRG1	3,6496
PDSS2	4,70013
PDZD8	2,04384
PEAR1	4,28471
PEBP1	-2,50815
PEG10	-3,43642
PELI2	6,0553
PER2	-2,54956
PEX1	3,17694
PEX14	-3,90757
PEX26	-4,25646
PFKFB2	-2,31438
PGAP1	4,19454

PGGT1B	2,09615
PGPEP1	-2,66847
PHEX	2,25485
PHF10	4,82016
PHF2	-3,9271
PHRF1	-2,62423
PHTF1	-3,22207
PI4K2B	-2,13378
PIBF1	-6,96616
PIGX	2,23211
PIK3R4	-7,38739
PIM3	-5,81201
PIN4P1	4,78247
PIP4K2C	2,4163
PITPNB	-3,91802
PJA2	2,74491
PKD1P1	7,80808
PKNOX2	2,45286
PKP4	-2,75534
PLA2G10	-2,48582
PLCB1	-3,82631
PLEK2	-2,02713
PLEKHA1	6,04856
PLEKHA2	2,30988
PLEKHA5	2,92121
PLEKHM2	-2,34651
PLS3	3,70997
PLXDC2	4,01698
PLXNA3	-2,38559
PMS2P9	4,21567
PNMA2	3,3265
PNO1	5,5075
PNPLA2	-2,65139
POFUT2	-4,51702
POLA2	-3,89084
POLB	-2,77412
POLE	3,60494

POLE3	-2,46442
POLG2	1,04465
POLR1A	1,95385
POLR1E	-2,76015
POLR2K	4,82099
POLR2L	4,59254
POLR3D	2,41338
POLR3E	-4,77309
POLR3K	-3,77322
POMGNT2	-4,39958
POMP	-2,9686
POU2F2	7,49215
PPARGC1A	3,92614
PPAT	-4,65426
PPEF2	5,92996
PPIP5K2	-3,66859
PPM1E	3,29095
PPM1H	3,18365
PPP1R14C	-2,64627
PPP1R35	-5,62803
PPP1R3A	-2,39774
PPP1R9A	3,46108
PPP3R1	-2,66361
PPP6C	2,89346
PPTC7	-3,37167
PRC1	-2,47914
PRDX2	5,48556
PRDX6	7,07209
PREB	-6,49882
PRELP	3,2619
PRKAA1	3,65108
PRKACA	3,91014
PRKAR2A-AS1	3,64872
PRKCA	2,81048
PRKCDBP	-5,55682
PRKCG	-2,68473
PRKRIR	-2,49414

PROX1	-6,37058
PRPF18	-1,98528
PRPF19	-3,90123
PRPF38A	3,69076
PRPF4	3,87093
PRR12	-2,53008
PRR14	-3,03269
PRR14L	-2,12145
PRRC1	-2,62296
PRRC2C	-3,06751
PSAP	-2,52568
PSMA5	4,6224
PSMB1	-5,17547
PSMB4	2,90261
PSMC1	3,25122
PSMD1	3,38187
PSMD14	2,53771
PSMD2	2,56358
PSMD5	6,51728
PSMG1	2,21593
PSTK	-4,35543
PTBP2	-2,25512
PTGER4P2-CDK2AP2P2	3,01847
PTGS2	4,67656
PTMS	-2,18772
PTN	-2,12212
PTPLB	-3,86132
PTPN23	-1,95362
PTPN9	3,78746
PUF60	1,09184
PWP1	-2,27601
PXDN	-2,23132
PXMP4	3,09198
PXYLP1	2,08986
PYCRL	2,92207
QDPR	-4,11903
QPRT	4,29107

QSOX1	-4,14642
R3HCC1	-3,35415
RAB11FIP2	2,25052
RAB11FIP5	6,9281
RAB18	-2,3528
RAB1A	-3,19808
RAB1B	2,39427
RAB20	-3,06699
RAB29	-2,46887
RAB31	5,29947
RAB33B	4,41682
RAB3GAP1	2,67863
RAB5B	3,39908
RAB8A	3,44506
RABL3	2,56654
RAD50	-2,73011
RALA	-2,44302
RALGAPA2	-4,7019
RASA3	-2,87158
RASEF	3,12109
RAVER1	-2,43425
RBBP6	4,74237
RBCK1	3,16677
RBFOX2	1,81967
RBFOX3	-3,2414
RBM11	2,80144
RBM20	-5,98552
RBM22	5,01598
RBM3	2,27254
RBM47	2,86441
RBMX	4,38639
RBP1	3,98854
RBPMS	4,13699
RBX1	2,58703
RCAN2	-3,70458
RCC1	-3,20241
RCN1	5,38323

RECQL5	-3,29235
REEP4	-4,35957
REPS1	-2,99307
RERE	-2,85971
RFTN1	5,24657
RGS7	-5,17662
RHOB	4,08096
RIOK3	-2,21999
RLN1	1,97786
RMND5A	3,13624
RMND5B	-0,48575
RND2	4,4161
RNF139	-7,25855
RNF144B	-3,97598
RNF181	2,98096
RNF185	5,21189
RNF2	4,55308
RNF213	-3,68292
RNF4	2,52714
RNF8	-3,04693
ROBO3	2,02996
ROR1-AS1	3,29334
ROS1	-2,3095
RP2	-2,82608
RP9	-3,46037
RPAP1	-1,9601
RPL11	2,74394
RPL13	-7,01114
RPL18A	-5,83983
RPL19	3,41566
RPL23	5,6419
RPL23AP53	5,54333
RPL27A	2,56183
RPL31	4,54186
RPL36A,RPL36A-HNRNPH2	4,08996
RPL7	3,24315
RPLP1	4,74281

RPN2	-3,07295
RPP14	2,60607
RPS11	-3,2422
RPS15	-2,11028
RPS16	-4,83923
RPS19	2,92208
RPS23	1,99764
RPS25	2,94884
RPS26	3,00221
RPS27	2,32165
RPS27A	6,0849
RPS28	-2,46155
RPS7	4,75505
RPS9	-1,90224
RQCD1	3,54697
RREB1	3,03187
RRP1B	-5,35258
RRS1	3,04419
RSL1D1	-3,19998
RSPRY1	-0,44158
RTCA	3,9789
RTN4RL1	-4,53198
RTN4RL2	-2,25672
RTTN	-2,38962
RUFY1	-3,72948
RUNDC1	-2,1682
RUNX1	4,44099
RUVBL1	-4,76024
RYBP	-2,97259
S100A10	5,0366
S100A11	7,11681
S100A3	-3,108
S100PBP	-5,53867
SACM1L	-3,57553
SAP18	2,23064
SAP30BP	-3,09141
SARS	-4,00854

SART3	-2,13452
SBK1	-3,26188
SBNO1	-2,30854
SCAF4	-2,12887
SCARB2	2,32831
SCD	2,56755
SCD5	2,25133
SCFD2	5,52647
SCGB3A2	-3,51386
SCRN3	-3,31522
SDCBP	2,17762
SDF2L1	-3,26338
SDHA	3,1019
SDHB	-2,51945
SEC14L3	4,36307
SEC23IP	3,79604
SEC61A1	-2,41654
SEC61G	-3,85801
SECISBP2	-2,32816
SEMA3A	2,72661
SEPHS2	-2,95878
SEPT14	3,57609
SERINC5	3,21037
SERP1	2,27199
SERPINB1	3,40225
SERPINF1	-4,08071
SESN2	-4,24771
SET	-4,3064
SETD3	-4,17114
SETD8	-2,34939
SF3B2	-1,89647
SF3B3	3,75056
SFI1	-5,28524
SFMBT2	3,49305
SFR1	4,17569
SGK1	2,75932
SGOL1	-3,71743

SGTA	-3,9637
SH3BGRL3	2,09506
SHANK3	-2,01771
SHC1	-2,43948
SHC3	7,63091
SHCBP1	-4,00205
SHFM1	-4,16451
SHKBP1	-2,88559
SHROOM1	-3,66778
SHROOM2	-2,52809
SHROOM4	7,46094
SIPA1L1	-4,23086
SIPA1L2	2,10463
SIRT5	4,25692
SLBP	-2,02664
SLC13A4	-4,35003
SLC17A5	-3,69284
SLC18A2	-4,95164
SLC1A2	-4,20655
SLC25A44	-3,24355
SLC25A46	-3,12447
SLC30A3	-4,79439
SLC30A6	3,69819
SLC33A1	-0,36266
SLC35A4	3,40019
SLC35B4	2,9303
SLC35F6	4,22039
SLC38A2	4,26772
SLC38A8	4,87511
SLC41A1	-2,13031
SLC43A2	4,87985
SLC45A3	-2,01819
SLC6A14	6,60759
SLC6A19	1,79917
SLC7A5	4,80064
SLC9A7	2,79631
SLCO2A1	2,08707

SLCO3A1	-2,08949
SLFNL1-AS1	3,15056
SLIT3	-2,91209
SLX4IP	-4,37081
SMARCB1	-4,63077
SMARCD1	3,67315
SMARCE1	-2,1626
SMCR8	-2,40753
SMEK1	2,36287
SMG1P7	3,04768
SMG5	4,71639
SMG7	-2,41821
SMIM7	-3,0581
SMPD3	-2,72519
SMYD4	-2,69562
SNAP23	-3,58614
SNAPC1	5,71542
SNRNP70	-5,44694
SNRPA1	-2,50587
SNRPC	-7,23658
SNRPE	2,7942
SNRPG	-3,14298
SNTA1	2,99704
SNX12	-5,77755
SNX19	2,39769
SORT1	2,69368
SOS1	2,4711
SP100	4,56108
SP3	-2,20605
SPACA3	3,08245
SPACA6P-AS	4,40502
SPAG5	4,30619
SPAG9	6,16212
SPARC	4,40729
SPATA7	3,04392
SPATS2	2,85113
SPDYC	-2,17881

SPECC1	-2,7177
SPINT2	-3,73538
SPPL3	-3,05185
SPRTN	-3,29178
SPTY2D1	-2,98222
SQLE	-2,72483
SRGAP1	2,63112
SRPR	-3,053
SRRT	-3,42991
SSFA2	-4,17227
SSR3	3,23161
SSU72	-2,49565
SSX8	-9,03444
ST3GAL4	4,92103
STAM-AS1	4,99186
STAM2	-2,1105
STAR	5,51748
STARD3NL	-4,61538
STAT2	4,55942
STAT5B	2,15081
STIM2	-3,49761
STK31	-4,05521
STK38L	-5,99293
STK40	3,31389
STMN1	-5,63711
STON2	5,98524
STRADA	-4,37367
STRIP1	-3,94315
STX3	-3,03363
STXBP6	3,21108
SUB1	2,10537
SUCLG1	-5,44985
SUGT1	-4,54006
SULT2A1	4,14935
SUN1	-2,32158
SUPT16H	-1,9236
SUPT20H	-4,76866

SUPT20HL2	-2,21255
SVIP	2,97143
SYAP1	3,22272
SYCP3	6,35712
SYNPO2	6,46978
SYPL1	3,11234
SYT11	2,138
SYT16	5,81538
SYT17	-4,88301
TAB3	2,61569
TACO1	2,39323
TADA1	2,32521
TAF10	4,82031
TAF2	4,25357
TAGLN2	-7,09693
TANGO2	2,77646
TAOK3	-2,37968
TAS1R2	3,42676
TAT	-4,26891
TBC1D10B	-4,13115
TBC1D15	2,51509
TBC1D9	4,02335
TBCA	4,46631
TBK1	-2,04557
TBKBP1	2,20832
TBL1XR1	-3,84887
TBP	-4,97246
TBPL2	-4,71581
TBXA2R	3,27138
TCEAL4	4,87936
TCEANC	3,3936
TCF25	-3,53444
TCF7	-2,54469
TCF7L1	-3,48366
TCOF1	-2,31462
TDGF1	3,43349
TDGF1P3	3,56651

TDRD12	2,22335
TDRD5	6,14734
TEAD1	4,10825
TEAD3	2,29865
TES	3,0972
TFAP2D	-7,51608
TFAP2E	3,01732
TFAP4	2,37456
TFCP2L1	-5,18909
TFE3	-3,16467
TGFBRAP1	-4,32485
TGM4	-2,33507
TGM7	6,86987
THAP6	-2,31437
THBS1	3,48872
THEGL	-6,02602
THEM4	3,27646
THG1L	-2,31653
THRAP3	2,11723
THRB	-2,86017
THYN1	-2,68026
TIGIT	8,62839
TIMM22	-2,98795
TIMM44	-3,34304
TINCR	3,99715
TLCD2	2,04319
TLR6	3,08168
TM2D3	2,91684
TM7SF3	-5,00157
TMED3	-4,48804
TMED5	3,36777
TMEM104	7,49825
TMEM106A	-2,37129
TMEM119	0,56455
TMEM132B	-3,42359
TMEM167A	5,72929
TMEM168	3,11096

TMEM17	4,69579
TMEM170A	5,36108
TMEM173	7,09738
TMEM184B	4,89267
TMEM185B	2,90304
TMEM19	2,65381
TMEM200A	2,65524
TMEM201	-2,98413
TMEM209	2,36969
TMEM212	2,04589
TMEM261	7,08833
TMEM39B	2,96543
TMEM50B	-2,29387
TMEM63B	-2,41327
TMEM63C	-2,74746
TMOD2	2,29513
TMOD3	-2,63729
TMSB10	3,05839
TMX4	-2,35816
TNC	3,69691
TNFRSF10D	3,66144
TNIK	-1,7889
TNKS	-6,14302
TNS4	8,18905
TOLLIP	-4,23673
TOM1L2	7,88348
TOMM22	4,03971
TOPAZ1	-1,86406
TOR1AIP2	3,68397
TOR3A	-2,83391
TOR4A	-3,68015
TP53INP2	2,55653
TPI1P2	-2,5779
TPM2	7,2782
TPM3P9,ZNF761	-2,9164
TPM4	2,85174
TPMT	3,06163

TPP2	-3,06493
TPTE2P6	4,75961
TPTEP1	0,02372
TRABD	-3,40122
TRAF6	-2,57806
TRG-AS1	6,1697
TRHDE	3,11405
TRIM21	-3,21939
TRIM29	-1,13223
TRIM36	3,08587
TRIM4	5,09929
TRIM44	-2,46296
TRIM59	3,32415
TRIM8	2,93414
TRIP4	-2,70553
TRIT1	-2,66404
TRMT11	2,5401
TRPV6	-4,57442
TRRAP	4,08248
TRUB2	2,31586
TSC22D1	-3,53843
TSG1	3,76748
TSHZ1	-3,95074
TSIX	2,59858
TSN	-3,23848
TSPAN6	5,84179
TSPYL2	-0,48028
TSPYL5	-3,64786
TSR1	-3,94508
TSR2	3,3658
TSSC4	-4,59642
TTC1	-2,4164
TTC26	2,99133
TTC5	2,42824
TTC9	5,72904
TTI1	4,19962
TTK	-2,13869

TTLL12	-3,81042
TTLL5	-2,29395
TUBA1A	3,18693
TUBB2A	4,53025
TUSC2	3,77334
TUSC8	-4,24978
TVP23C,TVP23C-CDRT4	3,45641
TWIST1	4,45731
TXLNG	-2,22382
TXNDC5	5,03142
U2SURP	2,52345
UACA	-2,56884
UBA2	2,39438
UBA52	4,22068
UBALD1	-3,89732
UBASH3B	-4,27438
UBE2C	4,82587
UBE2D2	-2,51397
UBE2N	3,03069
UBE2S	4,68552
UBE2T	-5,07593
UBL3	-2,65541
UBL7-AS1	3,22279
UBQLN2	-3,6923
UBR1	3,54384
UBR2	-2,72254
UBXN1	7,39423
UCA1	1,05055
UGT8	3,22821
ULK2	-2,52563
ULK4	-2,17285
UPB1	4,12936
UPF1	-2,10391
UPF2	-3,63711
UQCC2	4,55312
UQCRC2	2,61365
USP1	-3,91428

USP11	3,26945
USP2	-2,65129
USP22	-2,45591
USP30	3,64294
USP35	-2,08054
USP39	-4,44421
UTP6	-1,94188
VASP	1,02637
VAV3	-3,86223
VCAN	2,16844
VCL	2,40623
VCP	-2,66104
VIL1	-1,89886
VIM	4,16011
VIPR2	2,39609
VMA21	6,07725
VMAC	-2,82841
VPS25	2,04102
VPS26B	-2,46009
VPS33A	3,22253
VPS37C	-2,68431
VPS72	-3,03744
VSNL1	-2,66696
WBP11P1	1,16363
WBP4	-2,02946
WDFY2	2,7339
WDR1	3,59679
WDR37	-2,05578
WDR45B	3,35286
WDR7	-2,69497
WDR74	-6,09741
WDR75	-2,61831
WNK3	3,15717
WNT8B	2,8804
WTAP	-2,37586
WWC1	-1,95783
WWC3	-3,0108

XAF1	2,52135
XPNPEP3	2,90538
XPO5	1,95618
XPOT	-2,40592
XRCC2	2,96547
XYLT1	2,9386
YBX2	-3,58235
YEATS4	2,03098
YME1L1	-2,70572
YPEL2	-3,2295
YPEL5	3,48089
YWHAB	3,91212
YWHAQ	4,34553
ZAK	4,08589
ZAR1L	-2,16533
ZBED4	-3,18505
ZBTB24	-2,96579
ZBTB3	2,68926
ZBTB37	-2,98398
ZBTB6	1,96106
ZBTB8B	-3,8182
ZC3H10	-2,39837
ZC3H12D	3,03748
ZC3H18	-1,84733
ZC3H4	4,21106
ZC3HAV1	4,3679
ZC3HAV1L	4,20574
ZCCHC3	-2,88085
ZEB1-AS1	-3,23158
ZEB2	5,02674
ZFAND2A	-3,4906
ZFP1	-3,5677
ZFP3	2,91396
ZFP69B	-2,81298
ZFPM2	2,52601
ZFX	6,85747
ZFYVE26	-4,11417

ZHX2	-3,39231
ZIK1	-6,01532
ZKSCAN3	2,20653
ZKSCAN4	-4,26577
ZMIZ2	-3,6466
ZMPSTE24	-3,00469
ZNF10	-2,98395
ZNF132	-3,57091
ZNF135	4,37173
ZNF138	-3,29654
ZNF14	1,95097
ZNF140	3,31518
ZNF146	-2,192
ZNF148	-3,05411
ZNF18	3,51762
ZNF182	-4,63056
ZNF19	2,26358
ZNF2	3,9464
ZNF214	-2,61803
ZNF236	2,38726
ZNF251	-2,67603
ZNF252P	-2,83478
ZNF256	2,0654
ZNF268	3,26829
ZNF318	-2,3439
ZNF329	-4,26172
ZNF335	-3,84638
ZNF34	3,84932
ZNF37A	4,17796
ZNF394	4,12337
ZNF395	-2,98535
ZNF410	-2,64912
ZNF416	3,4587
ZNF426	-2,13635
ZNF430	4,57186
ZNF440	6,30714
ZNF461	3,5541

ZNF468	3,27262
ZNF471	-2,50979
ZNF486	2,5777
ZNF500	2,35677
ZNF502	3,14434
ZNF518B	3,01104
ZNF530	-2,88432
ZNF541	4,04205
ZNF544	-2,75128
ZNF548	-3,11602
ZNF555	1,98142
ZNF557	2,43367
ZNF559	7,96663
ZNF561	2,49755
ZNF564	4,42059
ZNF569	2,22528
ZNF584	2,17601
ZNF585A	5,65166
ZNF587B	-2,44217
ZNF589	1,85527
ZNF592	5,30774
ZNF606	7,26299
ZNF621	3,49331
ZNF629	-2,16386
ZNF670,ZNF670-ZNF695	5,78707
ZNF675	-8,56345
ZNF677	2,86408
ZNF681	4,15002
ZNF713	2,60687
ZNF747	3,36718
ZNF777	-2,36013
ZNF785	3,27894
ZNF815P	2,26834
ZNF823	4,88166
ZNF835	3,81684
ZNF844	-2,98051
ZNF879	-2,00776

ZNF880	-3,42241
ZNF90	-2,04226
ZNRF3	5,05576
ZP1	-3,10365
ZRANB1	-3,07476
ZSCAN25	-3,35405
ZSCAN5A	-7,47938

Supplementary Table 2. Genes expressed exclusively in oocytes < 35 years.

Gene
B3GNT1
CAMP
CD300LB
CNDP1
COX4I2
DPP7
GABARAPL3
HIST1H3J
HIST1H4D
IL17D
KCNJ9
LINC00895
LOC101928435
LOC101929412
MIR1179
MIR181D
MYF6
NEU2
NPVF
NT5M
OR10J3
OR10V1
OR4C13
OR52K2
OTOL1
PER4
PRG3
PRSS27
R3HDML
SCN1B
SLC38A3
SNCG
TRHR
TRIML1

Supplementary Table 3. Genes expressed exclusively in oocytes ≥ 35 years.

Gene
AAMDC
ACOT6
ACPT
ADAM2
ADRA2C
ANKRD39
ANTXRL
APCDD1L-AS1
APOH
AQP10
ASNA1
ATP11AUN
BCL6B
BOLA1
C14orf64
C6orf226
C8G
C8orf59
C9orf135-AS1
C9orf16
CATSPERD
CCDC53
CCL11
CDCA3
CLEC2A
COL24A1
COPS7A
CTD-2151A2.1
CYBA
CYP4Z2P
DNER
DSPP
ECHDC2
ESPNP

GALNS
GPR55
GSTM2
GSTM5
HEBP2
HIST1H4E
HIST2H2BE
HS3ST1
HSPA6
IFT52
IL13
IL22RA1
ITGAD
KLF17
KRT78
LINC00115
LINC00467
LINC00633
LINC00854
LINC01116
LMTK3
LOC101060019
LOC101927134
LOC101927843
LOC101928340
LOC101929234
LOC101929701
LOC102467655
LOC441204
LRFN3
MIR3648
MIR4454
MIR5186
MIR548I2
MIR612
MIR8061
NIPSNAP3A

OCEL1
OPTC
OR4K15
OR51S1
OR56A5
OR5E1P
OR6P1
OR8D4
PADI1
PAGE2
PDE6D
PISRT1
PMVK
PRAF2
PRDM6
PRICKLE3
PRR19
PYGL
REM1
RMRP
S100A8
SCNN1B
SH2D6
SLC17A1
SLC34A2
SPTLC3
SRY
TAAR5
TAF13
TAS2R16
TCERG1L
TCN1
TEX40
TIMMDC1
TMEM165
TMEM211
TMEM88

TNFSF18
TRNP1
TUT1
VWA5B1
WDR66
XYLB
YIF1A
ZNF768
ZNF792

Supplementary Table 4. Dysregulated genes in oocytes vs surrounding cumulus cells in females < 35 years.

Gene	Log2(Fold_change)
AGO2	-3,14513
AGO3	5,37782
AGO4	-3,26953
A2ML1	-3,69071
A4GALT	-2,33651
AARD	2,56672
AARS2	5,16849
ABCA9	3,08517
ABCB4	-3,52912
ABCB5	2,60019
ABCC5	3,76241
ABCC6P1	-4,53768
ABCE1	-2,62067
ABHD15	3,38538
ABL1	-5,67496
ABO	1,26941
ACAA1	6,21796
ACAD8	-3,2306
ACADVL	4,98009
ACBD7	2,75408
ACCSL	-3,97504
ACD	-5,99183
ACE2	2,36402
ACIN1	-1,96512
ACO2	-3,66784
ACOX1	5,26308
ACOX2	9,09216
ACOXL	-3,90213
ACP6	3,69943
ACTB	5,71982
ACTG1	2,81673
ACTG2	-5,34171

ACTL8	-6,46726
ACTR1A	4,18967
ACTR3B	-3,72641
ACTR3BP5	3,45512
ACVR1B	-3,40999
ADAD1	-4,42601
ADAM12	5,10422
ADAM19	-4,10141
ADAM30	3,91575
ADAM9	0,56455
ADAMTSL4	6,43577
ADAR	-2,89907
ADD1	2,73063
ADD3	-4,16604
ADH5	2,96758
ADHFE1	5,64185
ADM	7,9201
ADO	-2,31736
ADPGK	-2,39899
ADPRM	-4,08514
ADRB1	4,61847
AEN	4,03498
AFAP1L2	-3,55329
AFF1	-3,62347
AFF2	5,21816
AFTPH	4,7822
AGFG2	-3,9741
AGK	-4,55815
AGL	5,17861
AHNAK	6,62395
AHSA1	-2,99842
AICDA	3,13868
AIFM1	-2,08136
AIPL1	3,28249
AK3	4,41477
AK4	3,45203
AK5	-4,89066

AKAP1	-6,06052
AKAP11	-4,85266
AKAP5	3,42574
AKIRIN2	-4,72776
AKR1B1	2,78943
AKR7A2	3,56118
AKT3	-2,95875
AKTIP	-3,67548
ALAD	6,4869
ALAS1	-5,22499
ALDH1B1	-3,96266
ALDH3A2	4,78352
ALDOA	5,42121
ALG2	3,27163
ALG9	3,20455
ALKBH2	-5,95766
ALKBH5	-4,82877
ALKBH7	7,6174
ALOX15	-5,49042
ALPK1	4,68707
ALPK2	6,38509
ALPK3	3,85978
AMBRA1	-2,78533
AMD1	-2,36687
AMER1	4,94848
AMMECR1L	-2,60742
AMOT	-5,86144
AMZ2	-1,62425
AMZ2P1	6,09106
ANAPC16	3,07265
ANGEL1	3,82653
ANGPT2	0,47664
ANK2	3,32655
ANKDD1A	5,46368
ANKFY1	2,98612
ANKHD1	6,80291
ANKRD13B	-3,02288

ANKRD13C	-3,63568
ANKRD18A	6,64957
ANKRD20A12P	0,34035
ANKRD20A19P	5,23347
ANKRD20A9P	2,51302
ANKRD30BP2	2,19832
ANKRD32	-2,63972
ANKRD36B	2,96236
ANKRD36BP1	2,98806
ANKRD36BP2	5,13317
ANKRD42	-4,16509
ANKRD54	7,93524
ANKRD55	3,05401
ANKRD6	-4,69793
ANKRD65	3,06355
ANKS1A	-2,60305
ANKS4B	2,26341
ANLN	-5,91572
ANP32AP1	5,52124
ANP32B	-2,30258
ANXA2	8,48232
AP1M2	-3,19451
AP2M1	3,39466
AP4S1	3,5769
APBA1	-3,94878
APEH	1,12921
APITD1	-4,26791
APMAP	1,05005
APOA1BP	8,1113
APOBEC3A	4,3883
APOBEC3C	8,74919
APOBEC3D	3,35053
APOBEC3F	4,22343
APOBEC3G	5,67157
APOC1	2,37353
APOE	1,03923
APOL1	2,25508

APOOL	4,56547
APP	2,63659
APPL1	3,68828
APPL2	3,34427
APTX	2,88647
AQP11	5,79089
AQP3	9,3723
AQP6	4,4976
AQP7P1	4,59923
AR	3,69791
ARF4	3,32227
ARFGAP3	3,97276
ARFIP1	-3,07058
ARG2	-3,86261
ARGFX	3,9242
ARGLU1	6,08266
ARHGAP11A	-3,7337
ARHGAP15	-3,38122
ARHGAP19-SLIT1	-5,809
ARHGAP20	3,59696
ARHGAP23	1,05543
ARHGAP26	-4,01941
ARHGAP39	-2,56413
ARHGAP5	2,19493
ARHGEF10	5,8606
ARHGEF17	4,60125
ARHGEF28	4,30284
ARHGEF6	4,51357
ARHGEF7	-2,76625
ARID3B	-4,66401
ARID4A	-5,47555
ARID5B	3,15526
ARIH1	-4,16592
ARL11	5,45712
ARL13B	-2,49201
ARL14EP	-2,43498
ARL2BP	2,54205

ARL3	4,49642
ARL4A	-2,79095
ARL4C	-3,3107
ARMC1	-3,4649
ARMCX4	6,32925
ARPC5L	3,67701
ARRDC3	5,01559
ARSD	5,9491
ARSK	3,61813
ARV1	-3,68567
AS3MT	2,94822
ASAP2	-4,57297
ASB14	7,47015
ASB6	-4,15722
ASCC1	3,98102
ASF1A	-5,96881
ASNS	2,41184
ASPH	2,19996
ASPM	-4,94611
ASXL3	-3,22077
ATAD1	-3,55988
ATCAY	2,96231
ATF1	-4,97346
ATF4	5,26127
ATIC	2,80599
ATL2	-2,23132
ATMIN	-4,25421
ATN1	4,00665
ATP10B	-4,13094
ATP1B1	-2,83953
ATP2C2	-4,81484
ATP5O	3,58677
ATP6AP1	3,4307
ATP6AP2	4,02488
ATP6V0D1	-2,70027
ATP6V0D2	3,45145
ATP6V1A	5,09693

ATP6V1B2	-3,38044
ATP6V1C2	-4,53602
ATP8B2	4,30681
ATP9A	-4,18483
ATPAF2	-2,33203
ATRN	4,26892
ATXN2L	-3,79759
ATXN3	2,19279
ATXN7L2	-4,28788
AURKA	-6,80347
AURKC	-5,80602
AXIN2	-2,24755
AXL	3,25111
AZGP1P1	5,82437
AZI2	4,53605
B2M	7,42942
B3GNT2	-4,42296
B3GNT4	-2,69588
B3GNT6	2,82877
BACH1	-3,26679
BAG3	1,11022
BAIAP2	-3,95335
BAIAP2L1	-3,55839
BAIAP2L2	-2,99254
BANP	-3,512
BAP1	3,26117
BARD1	-3,69358
BARX2	-5,75065
BASP1	4,55882
BATF2	-4,88077
BAZ1A	-4,03488
BBS1	4,80414
BBS5	2,6336
BBX	6,21092
BCAR1	3,3048
BCAR4	-4,84965
BCAT1	5,81831

BCDIN3D	4,8874
BCKDHB	2,7768
BCL2L1	-5,98001
BCL2L10	-6,78836
BCL2L15	3,11688
BCORL1	-2,57569
BCRP2	-2,4218
BEST1	7,8034
BET1L	4,27392
BEX1	8,59692
BHLHE22	-3,30539
BHLHE40	-4,71741
BICC1	-3,44908
BIRC5	-4,29821
BLOC1S2	2,21312
BMP15	-8,98269
BMP6	-0,07253
BMP7	3,10685
BMPR1B	2,47802
BMS1P4	2,21389
BNC1	-5,21067
BNIP2	3,69752
BNIP3L	3,18322
BOD1	-4,6984
BOLA3-AS1	3,88831
BPGM	-4,675
BRAP	-3,89682
BRCA1	-2,02334
BRCA2	-3,87521
BRD1	-3,78338
BRDT	-2,74423
BRINP1	-2,27342
BRIX1	-3,23953
BRK1	3,56299
BROX	3,98923
BRS3	-3,78793
BRWD3	2,90644

BSDC1	3,13535
BSN-AS2	4,68587
BTAF1	7,28509
BTBD10	-2,96083
BTBD2	-4,06572
BTBD3	4,74214
BTBD7	-2,60013
BTG4	-9,42643
BTRC	-4,78053
BUB1B	-6,02462
BYSL	-3,30163
C10orf118	-3,3354
C10orf2	-3,28876
C10orf32	3,62939
C10orf35	3,88393
C10orf76	3,57783
C11orf54	3,07218
C11orf80	3,15629
C11orf82	-2,67409
C11orf84	-5,6613
C12orf65	2,91611
C12orf75	3,52246
C14orf105	3,32887
C14orf2	-3,28285
C16orf72	-4,64849
C16orf87	2,79749
C17orf103	3,79861
C17orf53	-3,43373
C17orf74	-3,88594
C17orf75	2,7223
C17orf89	6,30639
C17orf99	2,73916
C18orf61	4,7006
C19orf10	3,11275
C19orf12	5,13477
C19orf24	-3,14855
C19orf35	2,92279

C19orf48	4,56726
C19orf57	-4,42365
C19orf67	-3,17074
C19orf68	5,67358
C1orf220	5,4941
C1orf226	-5,68678
C1orf86	2,64637
C1QL1	-4,88152
C1R	2,85341
C20orf203	4,7123
C20orf96	-2,41839
C21orf59	-2,92499
C21orf62	2,34074
C22orf46	7,8531
C2CD2	5,92716
C2CD2L	-7,54946
C2CD3	-2,71373
C2CD5	-3,44573
C2orf49	3,72078
C2orf74	8,5516
C3orf36	1,022
C3orf56	-6,51781
C4orf27	-3,88341
C4orf47	-5,0007
C5orf15	4,59401
C5orf27	2,80113
C5orf28	4,32249
C5orf30	6,68423
C5orf34	-2,57214
C5orf51	2,9014
C5orf55	2,24297
C5orf64	4,24907
C6orf132	4,4323
C6orf203	-4,35547
C6orf99	9,04086
C7	6,12287
C7orf25	-4,57205

C7orf73	4,50857
C8orf12	-4,81503
C8orf31	3,46236
C8orf33	5,26972
C8orf49	4,60567
C9orf152	5,07696
C9orf3	2,30482
C9orf40	-3,13069
C9orf41	-6,35373
C9orf62	-2,00926
C9orf64	3,8745
C9orf91	-3,08936
CA5BP1	3,57354
CABP4	3,56382
CABYR	-3,83045
CACFD1	4,04702
CACNA1A	-3,39579
CACNA1C	4,08291
CACNA2D2	-2,96529
CACNG2	6,88346
CACNG4	5,14823
CACNG7	-3,78949
CADM1	-2,17479
CALB1	-5,66647
CALCOCO1	2,56971
CALCOCO2	-2,57138
CALML4	-4,18782
CAMK1D	-2,24342
CAMK2N1	5,80332
CAMP	-2,78917
CAMSAP1	-6,60479
CAP1	3,50853
CAPNS1	3,65011
CAPRIN2	-3,15635
CAPZB	3,75869
CARD6	-2,71151
CARD8	2,7401

CARS	3,22493
CASC1	-5,15131
CASC4	3,82643
CASC5	-5,127
CASC9	3,43851
CASK	5,79177
CASP14	6,27403
CASP8	3,1522
CAV2	4,57655
CBFA2T3	-4,10344
CBLB	9,25359
CBLL1	-3,08776
CBX1	-3,71897
CC2D1B	5,35819
CC2D2A	4,15052
CCAR1	-3,56982
CCDC101	-2,75583
CCDC11	2,30879
CCDC115	-2,91783
CCDC117	-2,84592
CCDC121	-4,2886
CCDC126	-2,63518
CCDC132	-2,44186
CCDC144B	4,00787
CCDC146	-2,83739
CCDC147	-2,74239
CCDC15	-4,72814
CCDC160	-0,76858
CCDC171	-6,45863
CCDC177	-0,28533
CCDC184	1,04275
CCDC27	-3,98383
CCDC34	-2,67018
CCDC43	-4,8249
CCDC58	2,64252
CCDC59	-3,053
CCDC69	2,9582

CCDC77	-3,97861
CCDC84	5,88537
CCDC88C	-4,26046
CCDC92	3,32524
CCDC94	-2,42191
CCL22	2,92445
CCL5	2,69624
CCNA2	-0,47901
CCNB1	-6,08448
CCNB2	-5,31942
CCNB3	-4,43124
CCND1	6,4376
CCNE1	-2,60585
CCNG2	-3,33083
CCNJ	-4,21523
CCNT1	-3,62203
CCNYL1	-3,884
CCSAP	-3,40563
CCT6A	3,79196
CCZ1B	-2,85315
CD24	5,49681
CD300E	5,78102
CD300LB	-5,71609
CD36	-3,99286
CD3E	0,52616
CD3G	3,22144
CD63	7,71889
CDA	-0,36876
CDADC1	-3,55468
CDC25B	-4,45096
CDC26	5,56193
CDC27	-2,12144
CDC42SE1	3,08832
CDC45	-4,73118
CDC6	-4,2901
CDCA2	-4,23338
CDCA4	-4,5659

CDCA7L	-4,98399
CDCA8	-3,22564
CDH1	-5,52977
CDH11	6,18142
CDH17	-3,19816
CDH2	2,8362
CDH3	-7,62934
CDH9	-3,64108
CDK12	-3,27108
CDK15	4,80261
CDK16	2,17985
CDK19	6,62432
CDK2AP1	3,14495
CDK2AP2	-2,91037
CDK5	-6,24577
CDK5RAP2	3,9394
CDK7	-3,73714
CDK9	2,61633
CDKL1	3,23174
CDKL2	-2,75159
CDKN1A	4,49317
CDNF	1,02872
CDR1	-4,72277
CDR2	-2,32877
CDR2L	5,31458
CDS1	-4,19454
CDV3	3,14766
CEBPZOS	-5,60976
CECR1	5,12194
CELF1	-2,25872
CENPA	-3,40055
CENPE	-4,61992
CENPH	-3,38556
CENPU	-5,36005
CEP170B	-2,89001
CEP19	0,32356
CEP55	-3,92039

CEP85	-3,10284
CERS5	2,27096
CES3	2,89688
CETN3	-2,56993
CGN	-0,34938
CHAF1A	-6,52255
CHAF1B	-3,39813
CHAMP1	-5,89111
CHD3	6,23528
CHD7	-4,17581
CHD9	3,99455
CHEK1	-0,60563
CHERP	-5,54928
CHGB	7,52869
CHMP7	-4,35146
CHN2	-1,91792
CHRM3	3,58403
CHRNA5	-2,99775
CHRNB1	3,59625
CHST1	5,52639
CHST12	-3,39954
CHST3	7,69548
CHST6	2,91931
CHTOP	3,61806
CHUK	-2,58492
CIAO1	2,83187
CIART	-5,37833
CIC	5,16494
CIITA	3,04713
CIR1	-6,55056
CIRH1A	-2,82455
CIT	-3,74875
CITED4	3,91417
CKAP2	-2,38468
CKB	3,25474
CKS1B	-4,45677
CLASP1	2,74718

CLASRP	2,96616
CLCN5	-4,13411
CLDN1	5,00516
CLDN11	5,56098
CLDN18	-3,5639
CLEC7A	3,42847
CLIP3	3,49731
CLK1	-2,55061
CLMP	5,07696
CLOCK	-3,52691
CLPTM1L	-3,06562
CLPX	-6,25102
CLSPN	-3,73563
CLU	3,43085
CMAS	5,93656
CMBL	3,68747
CMIP	3,992
CMKLR1	-3,36365
CMTM4	-3,7729
CMTR1	-3,96455
CNBD1	-3,83188
CNBP	-2,70124
CNDP1	-2,37481
CNGA4	5,36332
CNN1	3,45602
CNNM2	-5,42801
CNOT11	-6,59719
CNOT3	-3,15382
CNOT4	-3,18035
CNOT6	-3,3146
CNRIP1	-4,80757
CNST	-2,33205
CNTD2	-3,63265
COBL	2,99042
COG3	3,95609
COG7	5,03332
COIL	-2,59271

COL1A1	5,54034
COL27A1	9,84592
COL6A1	8,93974
COL6A3	4,99453
COL6A6	-3,22057
COLEC12	-2,75976
COLGALT1	2,72043
COMMD1	3,70421
COMMD4	-2,96607
COMMD9	4,61337
COPA	6,32131
COPS7B	-4,22523
COTL1	7,89175
COX15	4,70235
COX17	3,07089
COX4I2	-4,88304
COX6A1	3,58287
COX6B2	2,22376
COX6C	0,03366
COX8A	3,07092
CPLX4	-4,31066
CPM	3,53697
CPNE3	4,91242
CPNE8	2,42656
CPNE9	4,70351
CPQ	2,48824
CPS1	-2,67908
CPSF1	3,73292
CPT1A	3,25044
CR2	-4,86766
CRAMP1L	-3,17424
CREB1	-3,03822
CREB5	-3,30483
CREG1	7,51486
CRIPT	2,83872
CRKL	-3,68989
CRMP1	-3,21624

CROCCP2	6,20598
CRX	3,37133
CRY1	-4,64931
CRYGS	7,29645
CSDC2	5,20711
CSDE1	-2,75164
CSE1L	-2,62833
CSF1R	-4,26066
CSNK2A1	-2,23288
CSRP2	-2,61744
CST7	-4,04213
CSTB	-3,98598
CSTF1	-3,52056
CSTL1	-5,31861
CTAGE5	-5,84488
CTCFL	-3,02583
CTD-2201I18,1	-3,04404
CTD-3080P12,3	2,60013
CTDSPL	-2,96103
CTNNAL1	4,40552
CTNND1	-3,12964
CTSC	2,34655
CTSD	5,71419
CTSK	9,25638
CTSLP2	2,36232
CTSZ	4,36749
CTTN	2,00041
CTTNBP2	-4,36891
CUEDC2	5,28693
CUL4A	2,84322
CUL5	-5,39151
CUL9	5,10254
CUX2	-3,1002
CWC22	-3,42681
CWF19L2	-2,99046
CXADR	2,88554
CXCR1	4,72068

CXorf22	-2,64604
CXorf31	2,68329
CXorf38	3,09811
CXorf40B	-0,36462
CYB561D1	4,16859
CYC1	9,11362
CYFIP1	4,00086
CYP17A1	8,81346
CYP1A2	2,86419
CYP1B1	-3,01273
CYP2B6	4,1418
CYP2B7P	3,73836
CYP2E1	2,79802
CYP2F1	2,65644
CYP2G1P	1,09297
CYP2W1	3,48989
CYP4A11	3,40253
CYP4F11	5,07999
CYP51A1	3,47325
CYP8B1	5,50779
CYTH1	-2,76411
DAAM1	-4,08396
DAAM2	-3,58566
DAD1	-2,27143
DAGLB	-2,85052
DAND5	2,54654
DAPK3	-2,61453
DAZL	-6,27905
DBI	4,16252
DBIL5P	5,77808
DCAF10	4,53267
DCAF12L2	4,05406
DCAF5	-2,70392
DCAF8	3,24609
DCAF8L2	8,5979
DCAKD	2,54264
DCBLD2	6,24735

DCDC2	-6,42122
DCHS2	-3,40978
DCLK1	1,06042
DCLK2	-2,9323
DCLRE1A	-4,42361
DCLRE1B	-5,65013
DCLRE1C	3,63128
DCP1A	-2,49647
DCP2	-2,72664
DCT	-5,57727
DCTN2	5,80971
DCTN5	-2,46337
DCX	2,99082
DDIT4	6,82285
DDN	-2,70143
DDX10	-3,93456
DDX11	-5,51274
DDX19B	5,34578
DDX23	-4,78208
DDX4	-6,25489
DDX43	-4,6497
DDX47	-2,37061
DDX50	-3,56667
DEAF1	-3,1425
DEDD	-3,51815
DEFB116	6,19006
DEGS1	3,1358
DEK	-6,34104
DENND3	3,18129
DENND4C	2,56704
DENR	3,24293
DEPDC1B	-5,2103
DEPDC7	-6,2992
DFFA	2,45297
DGCR2	-4,2333
DGKZ	5,48803
DHCR24	5,02909

DHRS4-AS1	3,11969
DHX33	-2,83917
DIO3OS	5,94002
DIP2A	3,90928
DISC1	5,12858
DKFZP434A062	4,82796
DKFZP434L187	4,3221
DLG1	-2,39671
DLG3	-3,11601
DLG5	4,88219
DLGAP5	-8,28665
DLST	-3,66352
DLX5	2,61664
DMBT1P1	2,60759
DMD	-4,61189
DMRTA1	-3,79495
DMRTB1	-2,70014
DMXL1	-3,06523
DNAH10	-3,10854
DNAH11	-4,01921
DNAJB2	5,30757
DNAJC15	-3,34494
DNAJC2	-3,1882
DNAJC22	3,13022
DNASE2	5,02171
DNMT1	-8,69793
DNMT3A	-2,24783
DNMT3B	-3,72601
DNPEP	5,47572
DOC2B	-5,16068
DOCK1	-3,40369
DOCK5	3,77419
DOPEY1	-5,31236
DOPEY2	-3,85385
DPAGT1	5,30397
DPF2	-3,93536
DPM2	3,08709

DPPA3	-9,84055
DPPA4	5,3189
DPPA5	-1,05312
DPYD	3,46078
DPYSL3	8,10083
DQX1	-4,16398
DRD5	4,02852
DROSHA	-2,38008
DSCC1	-4,08748
DSEL	3,8986
DSG3	2,36445
DSP	5,00151
DST	3,74879
DTL	-5,59213
DTNA	5,16778
DTX2	-3,83885
DTX3L	3,80836
DUOX2	6,59302
DUSP10	-3,56036
DUSP11	-2,59629
DUSP3	3,59307
DUSP7	-5,50878
DUXA	3,19693
DYNAP	4,5543
DYNC1H1	4,57999
DYNLL2	-2,54717
DYNLRB1	5,02075
DYRK1A	-3,98984
DYSF	-4,30173
E2F1	-4,7733
EAPP	5,01008
EBF1	-6,30604
EBF2	-3,57047
EBLN1	-4,91682
EBPL	-5,10559
ECD	-0,23659
ECHDC3	8,77942

ECT2	-3,07627
EDAR	-5,85926
EEF1B2	4,86465
EEF1D	5,59234
EEF1G	3,78363
EEF2K	-3,72577
EEPD1	-3,96571
EFCAB14	-2,86679
EFCAB2	3,0842
EFCAB6	-2,45365
EFHD2	4,56275
EFNA5	-3,19213
EFR3B	-2,76358
EGLN1	5,14684
EHD2	3,46939
EHMT1	-2,04711
EID1	4,04345
EIF2AK4	-2,99866
EIF2B2	2,35973
EIF2B5	5,50168
EIF2S2	2,47269
EIF3D	2,40814
EIF3I	4,35618
EIF4A1	5,56379
EIF4E1B	-4,61531
EIF4EBP1	4,20182
EIF4EBP2	4,65629
EIF4ENIF1	-5,44893
EIF4G3	-2,77162
EIF5B	-4,56347
ELAVL2	-3,32601
ELF1	-2,72198
ELK3	-4,45071
ELL	8,97073
ELMO1	-5,30854
ELMO2	-2,89727
ELMOD1	4,1261

ELOVL2	-4,79318
ELOVL4	-3,86163
ELSPBP1	-4,40537
EMC3-AS1	6,02191
EMC7	2,91464
EMC8	-3,5337
EMD	6,01543
EML2	6,70366
EML5	-2,91757
EMR4P	2,51703
EMX2OS	3,98706
ENAM	2,52553
ENDOD1	5,29451
ENG	-5,25518
ENO2	1,05759
ENOSF1	5,55001
ENOX1	4,23927
ENPP1	-3,08126
ENPP4	-1,97158
ENSA	2,04327
EPAS1	-6,50905
EPB41L1	6,78806
EPB41L3	-3,74893
EPB41L5	-5,51791
EPC1	-3,20744
EPCAM	-5,48607
EPG5	2,82269
EPHA10	3,31909
EPN2	-4,46397
EPS15L1	-4,61659
EPSTI1	-3,09729
ERBB4	-3,40304
ERCC6	-3,18231
ERCC6L	-4,21895
ERG	-4,05091
ERLIN1	-0,32066
ERO1LB	-4,24926

ERP44	-2,7492
ERVK13-1	4,74522
ESCO2	-7,70095
ESD	4,59316
ESF1	-3,60281
ESPL1	-3,19283
ESRP1	-6,53256
ETNK1	4,88774
ETS1	-2,1773
ETV5	-5,7763
EVI5	3,47508
EVL	-2,78407
EXD1	2,51699
EXO1	-6,69149
EXOC3L2	3,38739
EXOSC6	4,13938
EXTL3	-2,61032
EYA1	-4,77031
F2R	3,15756
F3	5,84759
F5	4,26302
FABP3	3,8723
FABP5	5,2562
FADS6	2,50872
FAIM2	4,14022
FAM102B	2,55491
FAM104B	3,82815
FAM106A	4,164
FAM107A	4,60679
FAM107B	2,75263
FAM111A	4,76301
FAM111B	-2,34265
FAM114A1	4,50823
FAM115A	2,93563
FAM117A	-4,38183
FAM118A	-3,93938
FAM120A	2,49629

FAM122A	-5,01132
FAM129A	4,13168
FAM131C	4,31814
FAM134A	2,94175
FAM13A	-4,84692
FAM149A	-3,52341
FAM149B1	4,45546
FAM154A	-7,81164
FAM161A	4,4529
FAM163B	-2,00808
FAM167A	-4,03067
FAM167B	1,03294
FAM171A1	6,51781
FAM172BP	-5,49396
FAM174B	-2,79309
FAM175B	-2,46152
FAM184B	-3,33146
FAM189A2	-6,73889
FAM193A	-2,75883
FAM198B	5,48963
FAM207A	7,99247
FAM20B	3,07786
FAM214A	-2,55869
FAM216B	6,83788
FAM219B	0,68743
FAM220A	-7,33177
FAM222B	-4,17312
FAM227A	-2,74597
FAM230B	3,43616
FAM230C	6,57234
FAM46A	4,40747
FAM46B	-5,62521
FAM46C	-7,04458
FAM53C	-3,11848
FAM65B	-2,62323
FAM66C	0,48983
FAM71F2	3,08009

FAM73A	2,11533
FAM74A3	5,01247
FAM83A-AS1	9,62122
FAM83E	-6,12656
FAM83H-AS1	3,13083
FAM86C2P	-3,59535
FAM86EP	5,49304
FAM91A1	6,37169
FAM95C	5,97442
FAM96A	3,99064
FANCD2	-2,45266
FANCI	-0,41985
FANCL	-5,36376
FANCM	-2,74768
FAR2	-5,97158
FARSB	2,79066
FASN	5,99453
FBL	6,10315
FBLIM1	3,0925
FBLN7	-4,78781
FBN1	6,30958
FBXL12	2,75142
FBXL14	-4,07725
FBXL19-AS1	3,72573
FBXO18	-2,78139
FBXO30	-3,62402
FBXO32	6,25222
FBXO34	-3,39862
FBXO36	-0,21597
FBXO44	2,69863
FBXO46	2,91724
FBXO5	-5,30341
FBXO6	2,12314
FBXW12	-7,48653
FCHSD2	5,33359
FDFT1	-3,62009
FDX1	6,14414

FDX1L	9,07687
FDXACB1	3,36321
FEM1A	0,21473
FER	3,99686
FERMT2	-4,84948
FEZF2	-4,59588
FFAR4	4,21715
FGB	4,4753
FGD5	-3,32422
FGD5P1	5,21148
FGF11	5,52328
FGF18	4,83617
FGF5	2,5415
FGFR1	-2,47677
FGFR1OP	-3,02912
FGFRL1	3,0507
FHDC1	2,88923
FHIT	-4,2637
FHL2	5,36533
FHOD1	7,14875
FIGLA	-2,2339
FIGNL1	-3,76659
FILIP1L	3,71809
FIP1L1	-2,88919
FIZ1	2,58177
FKBP10	5,55863
FKBP14	3,16746
FKBP1AP1	5,53453
FKBP9	4,83553
FLJ20021	-3,03163
FLJ30403	2,60829
FLJ31104	2,92394
FLJ33360	4,4622
FLJ36000	2,09965
FLJ42102	3,06602
FLJ42627	3,0029
FLJ43315	4,2793

FLJ43681	-4,22062
FLNA	4,79579
FLRT2	9,73079
FLT1	3,32536
FMN1	-3,23741
FMN2	-4,40189
FMO6P	1,14107
FNBP1L	-2,29743
FNDC3A	7,36045
FNTB	-4,56306
FOXH1	9,06145
FOXJ2	4,48429
FOXJ3	-3,27283
FOXK2	-3,72299
FOXL2NB	3,62803
FOXM1	-3,00223
FOXN1	-0,29256
FOXN3	0,05839
FOXO1	0,03025
FOXO6	4,17346
FOXQ1	-6,93616
FRAT2	-2,88372
FRMD3	-2,33152
FRMD4B	-3,30097
FRMD5	7,04931
FRMD8	6,02201
FRS3	-3,87392
FSCN1	5,91859
FSTL3	0,54769
FTH1	6,63891
FTL	-3,92
FTX	3,02284
FUCA1	-2,81263
FUCA2	5,22814
FUNDC2	6,96703
FURIN	-2,87442
FYN	-3,80366

FZD2	3,24531
FZD3	-3,283
FZD4	3,05587
G0S2	5,62453
G3BP1	-3,73673
G6PD	-4,41732
GAB1	-6,27135
GAB2	-3,79043
GABARAPL3	-3,43812
GABPA	-2,28902
GABRR3	-0,42322
GADD45A	0,51964
GAL3ST4	2,26377
GALM	3,16588
GALNT11	4,03409
GALNT3	-5,26948
GAPDH	4,64455
GAREM	3,56645
GART	3,49901
GAS6-AS2	2,39919
GAS7	-4,85787
GATA1	9,68603
GATA6	7,67641
GATC	4,44081
GATSL2	3,21013
GBF1	-2,85032
GBP1	4,41638
GBP2	5,38329
GCC2	-4,65551
GCH1	-5,39881
GCLM	2,79827
GCNT3	-6,28371
GDAP1	-4,53916
GDF15	7,22996
GDF9	-7,87923
GDI2	-0,24669
GEMIN5	-7,73997

GET4	5,82007
GFOD1	4,75049
GFOD2	3,71262
GFRA1	3,05701
GFRA3	-2,48138
GGA1	3,23119
GGA3	-3,20429
GGACT	-3,10891
GGH	-2,24719
GGPS1	3,06393
GGT8P	4,66558
GID8	-4,05174
GIGYF2	-2,52733
GINS1	-3,52784
GINS2	-8,54802
GIPC2	-2,77674
GIT1	-4,3683
GIT2	-3,81479
GJA1	5,46204
GJB4	6,16025
GK5	2,89939
GKAP1	-3,83665
GLCCI1	-3,42168
GLDN	4,26156
GLE1	-4,54876
GLIDR	6,02119
GLUD1	6,857
GMCL1	-2,63676
GMDS-AS1	4,81517
GMNN	-4,80344
GNA11	-6,31645
GNA14	-5,13472
GNAI1	-3,02171
GNAI2	0,26937
GNAS	4,93592
GNAT2	-3,66448
GNB1	-2,56544

GNB5	2,98901
GNE	2,46636
GNL2	-0,44972
GNL3	-3,30665
GNPDA2	-3,08114
GNPNAT1	3,5242
GOLGA5	-2,96208
GOLGA8I	3,90874
GOLGB1	5,68384
GOLM1	-2,82469
GOLT1B	-2,86289
GORASP2	-3,81314
GOSR1	2,4702
GOT2	-4,4966
GP1BA	3,75869
GPATCH2	2,94482
GPATCH4	-2,71292
GPBP1	-3,11995
GPC2	6,10911
GPC4	5,49691
GPC6	5,21871
GPKOW	2,38477
GPM6A	-2,95739
GPN2	-2,87908
GPR112	5,28872
GPR119	3,53918
GPR137B	-2,82651
GPR143	-3,03815
GPR151	-4,17366
GPR160	-0,32254
GPR173	5,82577
GPR32	4,18221
GPR37	-2,92162
GPR37L1	4,61088
GPR50	2,06621
GPR56	5,67513
GPR65	3,16765

GPRC5A	6,89653
GPRC5B	7,19945
GPSM2	-4,7839
GPX7	4,4763
GRAMD1B	8,60571
GRAMD3	3,11573
GRB14	5,26627
GRB2	4,44887
GRB7	-8,30361
GREB1	0,28024
GRHL1	-3,29596
GRHL2	-4,87627
GRIK3	3,74556
GRIPAP1	4,07491
GRM4	-2,53269
GRN	5,59822
GSDMA	3,2521
GSDMC	3,89131
GSE1	-2,58811
GSKIP	-2,91017
GSR	3,13532
GSTA1	8,07079
GSTA3	8,54391
GSTA4	8,09829
GSTP1	6,05771
GSTTP2	2,81566
GTF2A2	2,29677
GTF2B	-2,82252
GTF2H1	-2,56498
GTF2H2B	4,83815
GTF2H5	2,51687
GTF3C3	-3,76268
GTF3C5	4,22018
GTF3C6	3,12487
GTPBP1	2,85442
GTPBP4	7,14824
GTSE1	-0,51805

GTSF1	-5,26874
GUCA1B	3,4789
GUF1	3,4818
GUSBP10	-2,77399
GXYLT1	3,39486
H1FOO	-8,36494
H2AFV	4,8007
H2AFX	-4,01016
HABP2	-8,38878
HADHB	6,0068
HAP1	-6,05914
HAS3	-4,98042
HAUS4	-3,32551
HAUS5	-6,18136
HAUS6	4,22205
HAUS8	-8,91107
HBE1	3,5721
HBS1L	2,47277
HCAR3	-3,44036
HCN4	-2,23415
HDAC3	2,82258
HDAC6	6,34038
HDAC9	-3,08705
HDC	-3,03568
HDGFL1	-3,22009
HDGFRP2	-2,51935
HDGFRP3	-4,30025
HDHD2	4,11991
HEATR6	5,24511
HECA	6,23776
HEG1	3,50374
HELLS	-5,69953
HENMT1	-7,50874
HERC2P4	2,40273
HERC6	-6,17094
HES2	3,10143
HEYL	-2,07067

HGSNAT	4,82122
HHEX	-8,09433
HHLA2	-2,21684
HIATL2	3,17167
HIBADH	3,12739
HIC2	-5,08682
HID1	-4,94647
HIF1A	2,34026
HINT2	-4,22697
HIP1R	-5,64715
HIST1H1A	-7,49953
HIST1H2AH	-3,3643
HIST1H4H	-3,59813
HIST2H2AC	-3,10543
HIVEP1	-4,03949
HIVEP2	4,99741
HJURP	-4,59264
HLF	3,72554
HMG20A	-3,10419
HMG20B	-4,26741
HMGA1	-5,16214
HMGA2	5,4275
HMGB1	-3,05983
HMGB2	-2,58716
HMGB3	-4,22099
HMGB3P1	3,17829
HMGCS1	5,38507
HMGN4	3,70275
HMMR	-4,9934
HMOX1	8,25445
HMOX2	2,20895
HN1L	5,46681
HNRNPA1P10	-3,45474
HNRNPA3P1	3,01548
HNRNPDL	0,24324
HNRNPM	3,15793
HNRNPU-AS1	4,34459

HNRNPUL2-BSCL2	-4,7061
HOMER1	-2,75626
HOMER2	-7,35133
HOMEZ	4,67612
HOTTIP	5,96959
HOXA4	4,51474
HOXA7	-4,07664
HP1BP3	3,55052
HPGDS	-2,92936
HPSE	2,10625
HRG	-3,35964
HRSP12	4,24796
HSBP1	4,45769
HSBP1L1	7,78005
HSD17B12	3,67271
HSD17B6	5,91109
HSD17B7	2,57635
HSF2BP	-8,13657
HSPA12A	3,73563
HSPA4L	-2,92739
HSPB6	5,12751
HSPE1	5,66424
HTRA1	6,48267
HTRA3	6,65515
HTRA4	4,40312
HUS1	0,51281
HVCN1	-3,19142
HYLS1	-4,75169
IBA57	3,22547
ICA1L	2,70015
ID4	1,08048
IDH2	-4,96649
IDI1	3,48023
IDO1	3,70383
IDS	2,67751
IER3IP1	-5,06504
IFITM10	4,57075

IFNAR1	4,66983
IFNL4	-6,71862
IGDCC4	6,29309
IGF2	4,1617
IGF2BP3	-6,64501
IGFBP1	-2,93534
IGFBP2	3,69197
IGFBP3	-6,91821
IGFBP5	3,4546
IGFBP7	7,75958
IGFBPL1	-2,84092
IGLON5	0,50823
IGSF10	-5,44843
IGSF11	-2,52618
IKBKAP	5,10958
IKZF4	3,33064
IL10	3,89355
IL10RB	3,83679
IL17D	-5,31728
IL1RAP	-4,30732
IL4R	2,6732
IL6	3,28058
IL6ST	6,40004
ILDR2	-2,37984
ILF3-AS1	3,36127
ILK	6,89484
IMP3	3,11577
IMPACT	4,98301
INCENP	-4,01241
ING2	-2,99727
INHA	6,24557
INHBA-AS1	4,20473
INHBB	7,51674
INIP	2,59706
INMT	2,96376
INPP5E	4,27887
INSC	-4,53046

INSL4	-3,6299
INSR	6,61602
INTS1	3,08923
INTS6	-3,00426
INTS8	-2,64248
IP6K2	4,28477
IPO5P1	3,52813
IPO8	-5,45135
IPW	4,81325
IQCB1	-4,28049
IQCD	-4,11348
IQCJ	-5,91927
IQGAP2	-5,43399
IQSEC1	-3,69238
IQUB	-5,04264
IRAK1BP1	-2,9281
IRAK3	4,07157
IRAK4	3,71605
IREB2	5,50898
IRF6	-6,4242
IRF8	-4,2255
IRGM	-5,72468
IRS1	3,54237
IRS2	7,03535
ISCA1	2,85484
ISG20L2	6,69532
ISOC1	-3,59029
ISPD	-4,43613
IST1	4,77674
ITGA2	6,8591
ITGAV	2,60424
ITGB1	3,46318
ITIH5	2,76119
ITM2B	5,30942
ITPR2	3,29924
ITPRIPL2	3,5325
IVD	2,92392

IWS1	-4,2992
JADE1	3,5166
JADE3	-3,15549
JAG1	-5,36233
JAG2	-4,28729
JAGN1	-4,74635
JAKMIP2	6,34848
JAM2	3,13618
JARID2	-3,96773
JHDM1D-AS1	2,82526
JMJD6	-4,08006
JOSD1	3,06077
JUN	4,38057
JUND	3,34927
KALRN	-4,16767
KAT2A	3,164
KAT7	-2,48145
KATNAL1	-3,31659
KATNB1	-3,91457
KCNA7	3,22756
KCND1	5,62616
KCND2	3,13062
KCNJ11	4,14009
KCNJ3	-2,74615
KCNK18	-3,19761
KCNK2	3,82506
KCNK3	6,17365
KCNK5	-3,74928
KCNK9	-5,15974
KCNQ1OT1	2,35492
KCTD12	7,27768
KCTD13	-4,17425
KCTD20	0,24466
KDELR1	5,85911
KDM1B	-6,07115
KDM5B	5,91989
KHDC3L	-9,33167

KHDRBS3	-5,30358
KIAA0020	3,30894
KIAA0247	-3,65257
KIAA0430	5,39152
KIAA0556	-2,30275
KIAA0895L	7,33365
KIAA0922	-4,22699
KIAA1033	-3,09436
KIAA1147	-3,30807
KIAA1211	-2,54248
KIAA1257	-3,94601
KIAA1429	-2,30008
KIAA1456	3,64443
KIAA1462	5,84067
KIAA1524	-7,52583
KIAA1549L	-3,18007
KIAA1614	3,03792
KIAA1751	4,27022
KIAA1919	2,71329
KIAA1958	4,38635
KIAA2026	3,9685
KIF14	-3,52247
KIF15	-3,40641
KIF18A	-3,89187
KIF1B	2,04447
KIF20B	-4,10908
KIF23	-4,71267
KIF26B	-4,56294
KIF3A	-4,01749
KIF4A	-4,99466
KIFC3	-2,92096
KIR3DX1	2,27358
KIT	-6,40878
KL	5,67799
KLC2	-4,31653
KLF10	3,92852
KLF11	-3,95352

KLF12	3,35549
KLF13	5,79679
KLF16	-4,09969
KLF2	-4,71609
KLF7	5,01008
KLF9	7,06794
KLHDC3	-3,35795
KLHL18	-3,25665
KLHL26	-6,3236
KLHL42	-6,09772
KLHL7	-2,96144
KLHL9	6,04598
KLK14	4,77916
KMT2A	3,4538
KMT2E-AS1	6,52205
KNSTRN	-3,65127
KPNA2	-4,77352
KPNA7	-7,60591
KPRP	-3,8702
KRAS	4,77005
KRBOX4	3,334
KRCC1	9,84022
KREMEN1	3,11984
KRIT1	3,2937
KRT16	-3,82967
KRT42P	5,05561
KRT5	-3,81546
KRT9	5,05812
KRTAP1-1	-5,43286
KRTAP5-5	9,47442
KSR2	-3,40869
KXD1	-3,77072
KY	4,18296
L2HGDH	3,06076
LACC1	6,25992
LAD1	-6,43014
LAMA1	8,29119

LAMB1	-2,92895
LAMB2	8,56259
LAMTOR4	1,02187
LANCL2	3,50817
LARGE	5,04908
LARP7	-4,35621
LARS	3,46718
LDHA	4,06009
LDHAL6A	-3,97491
LDHB	6,13886
LDLRAP1	-5,07172
LEAP2	2,93955
LEF1	-6,22549
LEF1-AS1	-5,70074
LEMD1-AS1	2,99273
LENG8	4,16041
LEPR	3,45815
LEPREL1	4,36129
LEPREL4	2,80433
LETM1	3,09487
LGALS3BP	2,5006
LGR4	4,59797
LHFP	8,43172
LHFPL2	5,95658
LHFPL5	4,45371
LHX8	-4,5854
LILRB2	4,96629
LIMA1	-4,91643
LIMK1	-2,92853
LIMS1	4,30045
LIN37	4,49904
LINC00032	-2,22395
LINC00052	4,61705
LINC00116	4,56674
LINC00174	4,80331
LINC00263	2,41315
LINC00284	-3,15748

LINC00311	2,67191
LINC00330	4,96733
LINC00337	-2,77632
LINC00346	2,29037
LINC00381	3,17483
LINC00410	2,85732
LINC00433	-2,24271
LINC00476	3,05701
LINC00488	-2,62775
LINC00501	2,07875
LINC00507	3,0161
LINC00547	2,90905
LINC00548	5,45887
LINC00606	4,66508
LINC00619	7,38429
LINC00622	5,21469
LINC00637	3,25382
LINC00648	4,46403
LINC00652	4,41459
LINC00657	5,50165
LINC00663	2,86029
LINC00670	2,93766
LINC00671	4,81979
LINC00674	3,91401
LINC00700	5,25159
LINC00704	9,83847
LINC00884	8,01321
LINC00887	3,31464
LINC00895	-9,22658
LINC00908	3,77636
LINC00910	3,27382
LINC00921	0,35949
LINC00924	2,87929
LINC00925	5,89911
LINC00942	3,63452
LINC00957	5,52608
LINC00958	3,08148

LINC00963	2,83521
LINC00970	3,25872
LINC00996	3,7225
LINC00999	2,56269
LINC01000	5,19176
LINC01012	3,47218
LINC01021	3,07567
LINC01069	5,70754
LINC01087	3,2566
LINC01118	-3,30107
LINC01128	-3,59796
LINC01134	3,59004
LINC01141	2,32061
LINC01152	-2,92308
LINC01160	3,31561
LINC01184	4,46023
LINC01204	2,67427
LINC01205	2,69734
LINC01212	3,02129
LINC01278	6,25404
LINC01299	4,1508
LINC01344	-4,92053
LINC-ROR	-2,38248
LINGO1	-2,25726
LIPG	-2,54911
LIX1	-4,44712
LLGL1	3,97274
LMBRD1	-3,86178
LMO4	-4,21727
LMO7	-4,1959
LMOD1	5,28039
LMX1B	4,32285
LOC100128233	2,28841
LOC100128288	2,63014
LOC100129138	3,26829
LOC100129935	8,85
LOC100130451	3,38348

LOC100130673	9,11801
LOC100130899	4,05575
LOC100131655	3,98215
LOC100133985	-3,35244
LOC100287015	2,83357
LOC100287846	4,19276
LOC100288069	3,96474
LOC100289019	3,89548
LOC100294362	5,50868
LOC100505812	4,05748
LOC100505817	-2,46694
LOC100506385	2,75462
LOC100506548	3,65322
LOC100506675	-4,04959
LOC100506688	4,04815
LOC100506730	4,47929
LOC100506746	4,30691
LOC100506844	-2,56828
LOC100506985	1,00244
LOC100506990	2,0615
LOC100507387	4,69398
LOC100507501	2,51492
LOC100507537	2,53124
LOC100996291	5,00153
LOC100996385	2,41043
LOC101926897	4,07637
LOC101926928	4,48754
LOC101926935	3,26831
LOC101927123	4,08423
LOC101927274	2,53496
LOC101927391	3,10069
LOC101927415	3,93549
LOC101927666	3,86311
LOC101927722	-3,61336
LOC101927740	2,83397
LOC101927797	5,36462
LOC101927865	-0,03512

LOC101927873	-2,53759
LOC101927881	3,77114
LOC101927931	7,25224
LOC101928053	2,39214
LOC101928063	-7,57044
LOC101928104	4,74996
LOC101928303	4,43537
LOC101928435	-4,06853
LOC101928567	2,68842
LOC101928738	3,2204
LOC101928992	3,68243
LOC101929010	2,50162
LOC101929173	5,03304
LOC101929549	4,17507
LOC101929567	2,48116
LOC102467081	2,19499
LOC102724316	6,60631
LOC102724927	3,63953
LOC143666	3,00263
LOC145837	-4,54607
LOC157273	3,28228
LOC220729	4,93595
LOC257396	3,79073
LOC283299	-4,82085
LOC283332	8,80687
LOC283335	5,18901
LOC283731	4,16732
LOC284080	5,54983
LOC284412	5,83241
LOC284581	0,03017
LOC284865	4,94795
LOC284950	3,52369
LOC285762	5,13002
LOC286367	2,2617
LOC339166	-3,32147
LOC339240	4,70432
LOC339529	1,16027

LOC339539	3,17681
LOC340113	3,56359
LOC389641	2,94368
LOC389765	4,42929
LOC389831	6,51676
LOC389834	5,54539
LOC389906	3,98469
LOC400685	4,41627
LOC400891	3,43257
LOC400958	5,76247
LOC440300	3,47947
LOC440896	5,14618
LOC441454	4,59566
LOC441666	3,63718
LOC493754	4,22134
LOC54944	3,141
LOC613037	4,85995
LOC642236	3,39228
LOC642929	4,42192
LOC643355	5,28881
LOC643387	4,89564
LOC643406	2,84975
LOC644669	3,86785
LOC644919	3,19026
LOC644961	-3,48417
LOC645434	-2,18013
LOC648987	3,37529
LOC728323	-7,0168
LOC728613	6,18571
LOC728730	-3,07096
LOC728752	2,90684
LOC729737	4,23507
LOC729987	3,51665
LOC730102	4,82777
LOC731424	3,01727
LOC93622	1,05154
LOH12CR1	-3,30105

LOH12CR2	3,86757
LONRF2	3,77323
LPAL2	2,69671
LPIN1	3,68977
LPP	2,63764
LRAT	3,93343
LRCH1	-2,21981
LRCH3	-4,45866
LRG1	2,68238
LRIG1	-4,0103
LRP10	5,4494
LRP6	-3,24234
LRRC1	-4,34073
LRRC16A	-3,24358
LRRC17	-4,2366
LRRC37BP1	4,85788
LRRC58	2,73152
LRRC59	-2,96267
LRRC7	-3,79757
LRRC75A-AS1	6,69638
LRRK1	-2,97271
LRRN4	-6,76935
LRRN4CL	3,49464
LRRTM4	-5,08832
LRTOMT	3,67916
LSG1	4,21555
LSM11	-3,40238
LSM14B	-2,48092
LSM3	0,05088
LSM5	4,36173
LSS	3,78361
LTB4R	4,68311
LURAP1L	5,48616
LUZP6	3,16819
LYAR	-5,10547
LYPD1	-3,42745
LYRM4	3,92635

LYZ	2,35724
LZTR1	5,35589
MAD2L1	-8,04116
MAEA	-2,9597
MAEL	-8,44963
MAF1	4,39146
MAFG	-3,12077
MAGEF1	1,05281
MAGT1	4,43972
MALAT1	5,01568
MALSU1	-4,20203
MAML1	4,14414
MAML2	7,38154
MAN1A1	4,73941
MAN1A2	2,85679
MAN1B1-AS1	3,31222
MAN2A2	-2,57134
MANEAL	3,98158
MAP1B	3,43875
MAP1LC3B	2,83885
MAP2K7	-5,17866
MAP3K13	3,83227
MAP3K5	4,02041
MAP7	-3,00885
MAP7D2	-5,02349
MAPK1	4,48298
MAPK13	2,78673
MAPK4	-2,87351
MAPKAP1	-3,41309
MARCH4	-3,22795
MAST3	-3,47586
MAST4	-2,82901
MATR3	2,56042
MAU2	0,44788
MAVS	2,79076
MBD1	3,4595
MBD2	3,43558

MBIP	-3,65918
MBNL1	6,33774
MBNL2	4,04345
MBNL3	8,39243
MC1R	9,30878
MCEMP1	-2,59789
MCF2L2	3,81296
MCFD2	2,67548
MCL1	6,51088
MCM10	-4,99727
MCM3	-4,19444
MCM4	-4,89147
MCOLN1	-3,37176
MCPH1	-5,61979
MCTP1	-4,89055
MDGA1	6,02218
MDH2	-2,63798
MDM2	2,19403
ME2	6,35529
MED12	5,2264
MED12L	-4,25429
MED19	-4,48572
MED24	5,59571
MED26	3,44097
MED28	9,54942
MED29	4,82625
MEF2A	3,05346
MEF2C	7,06987
MEF2D	4,63616
MEFV	2,71756
MEGF9	4,97617
MEIS3P1	6,01703
MELK	-3,33354
MEPCE	-4,50204
MESDC2	2,90641
MEST	-3,53276
METAP2	-3,57287

METTL13	3,24341
METTL16	3,76826
METTL21EP	-4,08227
METTL2A	3,24448
METTL4	2,8091
METTL6	4,40006
METTL7A	5,27148
METTL9	-2,27332
MEX3D	-4,92189
MFAP5	3,14166
MFHAS1	-3,68104
MFN1	-5,59697
MFSD6	6,12065
MGA	-2,90483
MGAT5B	-5,77301
MGC16275	4,30303
MGC70870	4,12609
MICAL2	-2,04553
MICAL3	-5,20646
MICALL1	4,9436
MICALL2	2,98101
MID1	-1,94057
MIDN	2,85502
MIEN1	9,02621
MILR1	0,37537
MIR600HG	9,34557
MIRLET7BHG	3,04465
MIS12	-0,47329
MIS18A	-3,52258
MITF	-3,96912
MIXL1	-4,30422
MKL1	-2,91049
MKNK2	-3,93028
MKRN2OS	3,25733
MLH3	3,85141
MLLT1	-3,36709
MLLT11	-2,79871

MLLT4	-2,43205
MLLT4-AS1	3,80775
MLYCD	-5,83856
MMD	5,11254
MMGT1	-3,67596
MMP14	5,81534
MMP19	7,35325
MND1	-3,09597
MOAP1	5,48574
MOB3B	-3,2245
MON2	-3,184
MORF4L2	3,13144
MPL	4,4402
MPP2	-7,42792
MPP6	4,42325
MPV17L2	-4,71421
MRC1	-4,80227
MREG	2,97839
MRGPRX3	3,32265
MRO	5,89541
MRPL13	-4,99946
MRPL16	3,15034
MRPL27	5,05977
MRPL37	3,09249
MRPL38	8,45419
MRPL4	-4,07602
MRPL44	-2,93227
MRPL45P2	3,52002
MRPL46	3,36963
MRPL50	3,47704
MRPS16	4,50346
MRPS25	4,23358
MRPS28	4,26562
MRPS30	-2,93108
MRPS31	-3,24782
MRVI1	-4,08912
MS4A10	2,96776

MSANTD2	3,17067
MSANTD4	6,05778
MSH2	-4,98658
MSL2	-4,95961
MSL3	-2,48204
MSMO1	4,67867
MSN	7,44512
MSRB3	2,71742
MSS51	2,96904
MST1	5,23914
MST1L	3,79583
MSX2	-2,97628
MT1A	4,007
MT1E	5,90065
MT1F	2,95164
MT1X	5,94291
MT2A	6,67272
MTCL1	4,01579
MTERFD1	-3,05838
MTERFD3	7,31038
MTFMT	2,55547
MTFR1	-3,79565
MTFR2	-3,82737
MTG2	2,70014
MTHFD2P1	2,61639
MTMR1	-2,9183
MTMR3	-5,52119
MTMR4	2,55065
MTMR9	3,02235
MTMR9LP	4,02567
MTRNR2L4	2,97477
MTRNR2L6	3,03254
MTRR	-3,05875
MTSS1	-2,38229
MTURN	6,33124
MTUS1	-6,33574
MUC15	4,18831

MUC17	4,5915
MUC2	6,92327
MUC6	4,97971
MUL1	-2,55166
MVD	7,30647
MVP	-3,20365
MXD1	-4,87642
MYBBP1A	-6,35247
MYBL2	-6,17244
MYC	7,28674
MYCBP	-6,17565
MYF6	-5,30657
MYH10	3,74663
MYH16	-3,76628
MYH9	4,03727
MYL12A	5,64249
MYL6	7,77031
MYLIP	-3,96588
MYLK2	2,54631
MYO1C	5,74947
MYO1H	-2,50652
MYSM1	3,07543
N6AMT2	3,18883
NAA11	-6,62221
NAA25	2,65506
NAA30	-3,18711
NAA35	-3,52725
NABP1	-2,90853
NACA	2,94452
NACAD	4,70717
NACC2	-2,1022
NAGK	4,70549
NAIF1	2,79125
NANOG	3,50722
NANOS1	-4,68538
NANS	4,99642
NAP1L1	-2,3793

NAP1L6	4,97189
NARF	-2,98994
NARS2	-4,23498
NASP	-5,04362
NAT10	-3,35454
NAV1	4,30264
NAV2	-2,42037
NAV3	-3,29141
NBAS	-3,24523
NBEAL1	-0,34846
NCAPH	-5,10278
NCBP2-AS2	-2,24984
NCCRP1	2,8018
NCEH1	-4,41899
NCK2	-4,15376
NCMAP	3,15571
NCOA1	2,1852
NCOA3	-3,48465
NCOA7	3,18568
NCOR1	-1,99655
NCRUPAR	3,08217
NDFIP1	4,22697
NDN	1,02335
NDP	4,80493
NDRG2	7,34881
NDRG4	-3,15627
NDUFA3	7,8689
NDUFA4	4,55938
NDUFA5	3,81067
NDUFA6	3,29841
NDUFA9	3,91706
NDUFAF2	9,03728
NDUFB10	5,06036
NDUFB2	4,47607
NDUFB7	3,72897
NDUFC1	4,99015
NDUFS4	3,13861

NDUFS6	1,09567
NEAT1	5,39962
NEB	-2,36059
NEBL	2,90189
NECAB2	4,8196
NECAP2	-3,42176
NEDD1	-5,90509
NEFH	-3,28902
NEFM	-4,55407
NEIL3	-3,05918
NEK1	-6,59468
NEK2	-1,91953
NEK3	6,32368
NEK5	2,56662
NEK8	2,39704
NEK9	4,07172
NELFCD	3,68692
NEO1	-5,70491
NETO2	-4,1996
NEU2	-3,59213
NFAM1	4,77183
NFAT5	5,80079
NFATC1	-3,63279
NFATC3	-3,05987
NFIB	4,90864
NFIC	2,37735
NFKBIA	-5,14788
NFRKB	-3,48251
NFX1	4,06156
NFXL1	-4,97749
NGLY1	3,00832
NHLRC3	-2,78293
NHSL1	-2,89922
NICN1	4,72234
NIF3L1	-2,57186
NIT2	3,61226
NKAIN1	7,16644

NKX3-1	-6,31939
NLGN2	3,01287
NLRP13	-8,61104
NLRP14	-3,1539
NLRP2	-7,35474
NLRP4	-9,01194
NLRP5	-7,26157
NLRP7	-6,69307
NLRP8	-3,0886
NLRP9	-8,19206
NMD3	-3,65485
NMNAT1	3,0641
NMUR2	2,98476
NNMT	1,10307
NNT	4,45525
NNT-AS1	4,97847
NOA1	3,81948
NOL12	4,51847
NONO	4,61693
NOP10	2,96479
NOP9	6,22068
NPAP1	2,60146
NPC1	-3,80449
NPC2	-3,17027
NPIPB5	5,27633
NPM1	-2,0737
NPM2	-6,97892
NPM3	-6,20436
NPTX2	4,56125
NPTXR	3,5368
NPVF	-2,91594
NQO1	3,20407
NR1D2	3,94217
NR2E1	-5,68622
NR3C1	3,24361
NR5A2	4,2299
NR6A1	-3,79364

NRAP	-4,03412
NRBF2	4,40668
NRIP2	3,86131
NRP1	0,65512
NRSN1	-2,37952
NRSN2	6,89776
NRXN3	4,1569
NSD1	4,28359
NSMCE2	-2,35598
NSMCE4A	-3,58735
NT5M	-2,73998
NTAN1	-3,722
NTMT1	-9,90122
NTN4	4,40732
NUAK2	2,90222
NUBP1	6,71187
NUDT19	5,04773
NUDT9	4,65372
NUF2	-4,16843
NUGGC	2,73726
NUMA1	-2,8635
NUMB	-4,47338
NUMBL	6,32081
NUP153	-3,05854
NUP214	-3,44776
NUP35	-5,32647
NUP37	-4,24439
NUP50	-5,33934
NUP54	-2,53742
NUP98	-3,59987
NUPL1	-3,412
NUTM2D	6,10722
NWD2	-4,08139
NXPE3	3,00438
OAF	-1,94258
OAS3	-2,89049
OAT	-3,61288

OAZ2	2,97706
OCIAD1	-2,29209
OCLN	3,27768
ODC1	-3,60563
ODF2	-2,98205
ODF2L	2,74183
OGFOD1	-0,32067
OGT	3,91761
OLA1	4,32197
OOEP	-6,01223
OOSP2	-5,0842
OPA3	4,51784
OPRM1	2,63946
OR10V1	-4,42777
OR13G1	-4,58576
OR14I1	3,81173
OR1J4	-3,07709
OR1L8	-1,93058
OR1N1	-7,77671
OR2AT4	8,85782
OR2T27	2,22326
OR2T4	-3,76358
OR4N2	3,57551
OR4X1	3,87266
OR52K1	-3,16259
OR52K2	-2,78489
OR6C68	-3,48244
OR6V1	3,85977
OR6W1P	3,31131
OR7D2	3,59139
OR7E14P	3,59491
OR7E2P	2,51645
OR7E37P	3,51274
OR8G2	-2,54445
ORAI2	3,71914
ORC3	-3,51945
ORC5	-4,05308

OSBP	-3,67714
OSBPL10	-3,92179
OSBPL5	-4,06621
OSER1-AS1	3,71705
OSMR	4,93092
OSR2	-3,7333
OTOGL	-2,78328
OTOL1	-2,19516
OTUD3	-3,35902
OTUD6A	4,60647
OTUD7A	-6,05413
OTX2	-9,43005
OVCH2	-2,61476
OXNAD1	-5,39001
OXTR	7,28954
P4HA2	4,03046
PABPC1P2	2,47613
PABPN1L	-7,18902
PACSIN2	2,9561
PADI6	-5,35454
PAG1	-3,78546
PAIP1	-4,42478
PAIP2B	-2,64586
PALB2	-4,90038
PALM3	-3,56936
PAM	6,70189
PAPD7	-6,40547
PAPLN	3,88443
PARM1	3,3099
PARP1	-3,07372
PARP11	4,26376
PARP12	-4,38548
PARP14	3,90617
PARP16	-4,31478
PARP4	6,40034
PARP6	4,38575
PART1	3,89729

PATE1	4,6253
PATE3	8,9532
PATL2	-5,1978
PAXBP1-AS1	3,86181
PAXIP1	-4,54327
PCAT18	5,27164
PCDH11Y	2,58049
PCDHB19P	-3,59963
PCDHB9	3,08946
PCF11	-3,98654
PCGF1	-4,90252
PCMTD2	-4,46837
PCNA	-6,6188
PCNX	3,94026
PCP4	8,12067
PCP4L1	-5,15819
PCSK7	1,18046
PCYOX1	4,00592
PCYOX1L	-4,03138
PDCD10	-3,41623
PDCD2L	-6,17637
PDCD4-AS1	4,58801
PDCD5	-3,68843
PDCD7	-2,49006
PDCL3	-5,3849
PDDC1	2,79271
PDE12	3,53566
PDE3A	-2,69464
PDE4DIP	6,20656
PDE6C	-3,73736
PDE8B	-4,35781
PDK1	-3,46046
PDK2	-3,44511
PDLIM5	2,22825
PDP2	2,69806
PDPN	-1,93518
PDS5A	4,17163

PDSS2	3,35395
PDXDC1	-1,92817
PEA15	8,77758
PEAR1	3,62516
PECAM1	-2,23928
PECR	-2,26111
PEG3	7,27079
PELI1	-3,11108
PELI2	6,22761
PER2	-3,99159
PES1	-3,01221
PEX26	-3,54904
PFKFB2	-2,57729
PGBD5	2,25078
PGLYRP4	4,28035
PGR	5,0136
PGRMC1	5,81867
PHACTR3	-5,54512
PHF10	4,61824
PHF12	-3,34898
PHF2	-2,58367
PHF5A	4,26849
PHF6	-2,17869
PHF8	-5,00587
PHLDA1	3,96365
PHRF1	-1,88933
PI4K2A	5,6893
PIAS1	-3,95216
PIAS4	-3,32772
PIBF1	-7,04598
PIFO	4,90126
PIGM	4,94737
PIGS	5,53362
PIGU	-3,79352
PIGX	2,88454
PIK3C2B	-2,91765
PIK3C3	3,61304

PIK3CA	-2,64378
PIK3R3	-4,4361
PIK3R4	-7,13558
PIM2	2,36323
PINK1-AS	5,35407
PINX1	-5,15019
PIP4K2C	-2,88607
PIPOX	4,84687
PIPSL	2,53062
PITHD1	-2,86205
PITPNB	-3,58956
PIWIL3	-4,21529
PKD1	4,47286
PKD1P1	5,47057
PKI55	9,82512
PKP2	4,54645
PKP3	5,81972
PLA2G12A	5,76155
PLAG1	-3,03264
PLAGL1	2,57298
PLAGL2	-3,71969
PLCE1	-5,61398
PLCH1	-2,86918
PLCH2	-2,13633
PLCL1	-3,34169
PLCXD3	7,22186
PLEK2	-3,68226
PLEKHA2	3,56022
PLEKHA7	-4,8235
PLEKHB1	-2,62043
PLEKHF2	-4,08977
PLEKHG1	-3,80049
PLEKHG2	5,16945
PLEKHG5	-2,93595
PLEKHH1	5,28844
PLEKHM2	-4,23447
PLIN5	4,30035

PLK1	-3,60586
PLLP	-3,1643
PLP1	6,05266
PLP2	4,23368
PLS1	-2,55157
PLS3	5,62161
PLSCR1	5,14793
PLXDC2	5,79312
PLXNA1	7,3291
PLXNB2	5,8146
PMS2CL	5,02671
PMS2P9	5,54483
PNISR	4,22946
PNLDC1	-7,16512
PNMA1	3,65355
PNMA2	3,25521
PNO1	3,37581
PNPLA2	-2,52627
PNRC1	4,66078
POGK	6,01819
POLA2	-4,56374
POLB	-5,43592
POLD3	-4,14252
POLE3	-2,61204
POLE4	2,74879
POLR1D	-3,16016
POLR1E	-4,1129
POLR2B	3,45181
POLR2E	4,41736
POLR2K	3,01656
POLR2L	5,27902
POLR3E	-3,48634
POLR3G	-6,14738
POM121L8P	2,96297
POMK	-4,28942
PORCN	8,9895
POT1	3,01637

POTEM	3,84103
POU2F1	-3,18896
POU2F2	4,47299
POU2F3	-4,70525
PP7080	9,11407
PPARD	-2,68343
PPARG	6,7131
PPAT	-4,10235
PPFIA1	-2,34844
PPFIBP1	3,75398
PPFIBP2	-4,06208
PPIE	7,19491
PPIEL	3,47238
PPIL4	-3,41722
PPIP5K2	-3,49065
PPL	-2,48116
PPM1D	-2,39326
PPM1G	-3,44368
PPM1H	3,34105
PPM1K	2,90725
PPP1R13B	-2,60633
PPP1R14B	5,10682
PPP1R15A	3,11154
PPP1R15B	3,47165
PPP1R35	-3,40888
PPP1R3C	-5,02866
PPP1R9A	2,75629
PPP2R3C	-3,19832
PPP3R1	-3,02005
PPRC1	-4,07617
PRAM1	-4,23566
PRAME	1,06489
PRAMEF12	6,00635
PRC1	-7,34032
PRCC	-0,02524
PRDM10	-2,42227
PRDX2	6,23869

PRDX3	3,01005
PRDX4	1,18927
PRDX6	6,82576
PREB	-6,30057
PRELID2	-3,13953
PRELP	3,50576
PREPL	-4,29592
PRG3	-3,26732
PRIM2	-3,35858
PRIMA1	5,72607
PRKAR2A	-3,05856
PRKAR2A-AS1	2,8381
PRKCA	5,05222
PRKCDBP	-2,54147
PRKCG	-4,36439
PRKCH	0,53774
PRKCI	0,31118
PRKCQ	-5,32818
PRKD3	0,55572
PRKDC	4,70591
PRKRIR	-3,31825
PRKXP1	2,32632
PRLR	7,36681
PRND	5,94662
PROSER1	-6,76882
PROX1	-3,48669
PRPF18	-4,59268
PRPF19	-3,19952
PRPF40A	-3,11681
PRPF4B	5,34909
PRR14	-3,92921
PRR15	-4,97242
PRR7-AS1	3,99913
PRRC1	-3,5271
PRRG1	4,61598
PRRT2	3,59464
PRSS23	7,07764

PRTG	-4,52865
PSEN2	-3,46363
PSIP1	-4,86817
PSMB10	8,5655
PSMB4	4,85094
PSMC3	5,6924
PSMC6	3,949
PSMD4	4,74413
PSMD5	2,98946
PSMG1	-3,06534
PSTK	-2,69221
PTAR1	6,25209
PTBP2	-3,93578
PTCHD4	3,71105
PTGER4P2-CDK2AP2P2	3,52451
PTGES	5,62829
PTGES3	-3,77433
PTGIS	4,27871
PTGS2	4,59676
PTK6	2,47949
PTN	-8,82652
PTPLB	-4,97449
PTPN3	-2,36628
PTPRCAP	4,87508
PTPRG-AS1	-3,35208
PTRF	4,51442
PTS	9,6765
PTTG1	-7,63784
PUF60	5,62717
PUM1	1,98312
PURB	6,18375
PUS7	-3,06162
PVRL3	-1,99079
PVRL4	6,55536
PWP1	-3,27627
PWRN1	3,79283

PWRN2	5,83084
PXK	-2,40888
PXMP2	3,9782
PXMP4	3,20286
QDPR	-3,13067
QPCTL	2,64052
QPRT	5,07881
QRFPR	3,18871
QRICH1	-3,48627
QSER1	4,01243
QTRT1	3,10595
R3HCC1	-2,6324
R3HDM4	-2,71948
RAB11A	2,85183
RAB11FIP2	2,95485
RAB11FIP4	4,87292
RAB14	5,36445
RAB1A	-3,01846
RAB1B	4,03046
RAB30	-3,40297
RAB30-AS1	3,85819
RAB31	8,06308
RAB34	7,43937
RAB38	4,64426
RAB3IP	4,06682
RAB6A	3,13094
RABAC1	2,23347
RABGAP1L	-2,20836
RABL3	3,54814
RAC2	2,69104
RACGAP1	-4,50234
RAD23B	-2,17564
RAD50	-3,97272
RAD51C	3,23238
RAET1E	5,33236
RALA	-3,11364
RALGDS	6,94062

RALGPS1	-3,14091
RANBP10	2,14534
RAP1GDS1	4,02767
RAP2A	4,23607
RAP2C	4,10639
RAPGEF4	7,39978
RAPGEFL1	3,89563
RASA1	-2,66849
RASA3	-5,10951
RASAL2	2,84073
RASGRP3	-3,10757
RASL11A	-5,20096
RASSF3	-5,70659
RASSF9	5,09833
RAVER1	-2,38616
RAX2	-3,27101
RB1	2,9339
RBBP4	-3,24382
RBBP6	4,48951
RBBP7	-3,20171
RBBP9	2,81518
RBFOX3	-2,18993
RBL1	3,09457
RBM10	5,83288
RBM19	4,40339
RBM20	-2,20974
RBM3	3,34711
RBM39	0,02956
RBM41	4,36779
RBM42	2,77381
RBM46	-4,53635
RBM47	3,99818
RBMS2	3,98906
RBMX2	-4,28624
RBP1	7,78434
RBPMS2	-6,38958
RCAN2	-5,82327

RCBTB1	3,01295
RCBTB2	-4,35106
RCC1	-3,48342
RCC2	-4,39455
RCE1	8,30836
RCN1	8,02797
RDH11	5,17723
RDX	2,7971
RECQL5	-4,69942
REEP3	4,95996
RELB	3,85108
REPS2	-3,98411
RERE	-2,82854
REREP3	-2,15696
REV1	-2,86623
REXO1	-3,97007
RFTN1	5,81228
RFTN2	4,78926
RFWD2	3,24831
RFWD3	-4,14412
RFX7	3,35789
RGL1	4,91709
RGMA	-4,04103
RGPD4-AS1	3,7811
RGS2	-6,45919
RGS7	-4,90248
RHBDF1	4,02925
RHBDL2	2,6269
RHBG	3,23057
RHEB	4,5868
RHO	9,04924
RHOB	7,44097
RHOQ	3,24555
RIMBP2	-3,23348
RIMKLA	-3,45212
RIN2	5,47691
RIOK1	-0,04468

RIOK3	-3,15483
RIPK1	5,2263
RLF	-3,17014
RLN1	2,03453
RMI1	-3,19409
RMI2	-3,70748
RMND5B	-4,56418
RND1	-4,20907
RND2	2,75885
RNF10	-3,37297
RNF111	-3,65147
RNF114	-2,81486
RNF122	-5,98089
RNF139	-4,99133
RNF139-AS1	4,78361
RNF141	4,74395
RNF144A	3,17855
RNF144B	-2,24932
RNF146	3,49521
RNF17	-5,667
RNF170	2,32129
RNF180	-6,08205
RNF181	2,82105
RNF19A	2,693
RNF20	-3,64542
RNF213	-3,0561
RNF219	-3,73495
RNF222	4,34297
RNF31	4,83657
RNF34	-5,79072
RNF38	-4,70273
RNF7	4,36756
RNF8	-2,34855
RNFT1	2,85666
ROR1-AS1	2,95661
ROS1	-5,352
RPA3OS	-4,32745

RPF1	-3,51646
RPGRIP1L	-3,41227
RPH3A	-4,89493
RPIA	-3,16826
RPL10	2,72728
RPL11	2,31014
RPL17	7,03182
RPL19	6,67023
RPL22	3,75899
RPL22L1	5,1172
RPL23	7,7939
RPL23AP53	3,96619
RPL24	2,77704
RPL27A	5,27133
RPL28	3,20848
RPL31	5,624
RPL34	5,61235
RPL37A	2,89045
RPL38	4,4768
RPL4	3,42891
RPL7	3,15485
RPLP0P2	2,81221
RPLP1	5,10252
RPRD1B	5,27762
RPS10	5,33867
RPS15A	5,73171
RPS19	4,86447
RPS25	4,52537
RPS26	3,62216
RPS27	5,14264
RPS27A	5,68003
RPS27L	2,38963
RPS29	3,71651
RPS3	4,51681
RPS3A	3,73431
RPS5	5,35441
RPS6	4,97765

RPS6KB2	2,2166
RPS7	4,55615
RRBP1	2,94171
RRM2	-2,97206
RRN3P1	6,15116
RRP7A	3,46514
RRS1-AS1	5,35167
RSAD1	2,80676
RSL1D1	-4,15097
RSPH3	4,56617
RSPO2	-6,07259
RSPRY1	-3,23472
RTCA	4,45581
RTN4RL2	-3,08654
RTTN	-3,58673
RUNX1	5,47918
RUNX1T1	-3,96966
RUNX2	8,67011
RUVBL1	-2,29128
RWDD4	3,20724
S100A10	8,34623
S100A11	9,48544
S100A13	7,66799
S100A16	7,80206
S100PBP	-2,85271
S1PR1	5,41914
SACM1L	-5,05894
SAFB2	3,18983
SAMD1	-5,8454
SAMD4A	-1,89629
SAP130	-4,89602
SAP18	2,91263
SART1	6,41827
SASS6	-5,25768
SAT1	7,51383
SBF2	4,75753
SCARF1	3,95892

SCD	4,9764
SCD5	2,25998
SCFD2	5,29514
SCGB3A2	-7,54488
SCLY	-5,01104
SCN2A	-2,58557
SCRG1	9,91479
SCUBE1	-2,09403
SCUBE3	4,93184
SDC4	5,71814
SDE2	-4,20259
SDHAF2	9,68281
SDHB	-3,15486
SDR42E1	3,90736
SDSL	3,02621
SEC14L6	-1,96178
SEC23A	6,17632
SEC24A	-2,29037
SEC24D	6,14959
SEC31A	3,29132
SEC31B	2,99543
SEC61B	4,40484
SELK	2,54729
SEMA3A	5,57549
SEMA3E	3,57731
SEMA4B	-3,58348
SEMA4F	2,87838
SEMA6A	3,95895
SEMA6D	7,87851
SENP8	-5,40019
SEPT6	3,52346
SEPT7P2	2,76016
SERBP1	1,91526
SERHL2	-5,02758
SERINC1	3,14251
SERINC2	-0,28416
SERPINA3	7,04812

SERPINB1	4,48422
SERPINB8	3,94921
SERPINB9P1	5,73263
SERPINE1	7,81521
SERPINF1	-4,72151
SESN1	-3,06435
SET	-4,23258
SETD3	-4,34295
SETD5	0,35019
SETD7	4,59082
SETDB1	-2,85638
SF3B2	-3,39724
SF3B4	-3,16613
SF3B5	4,50778
SFI1	-4,65244
SFRP1	4,00758
SFRP5	9,8228
SFT2D3	9,82081
SFXN1	3,3286
SFXN3	3,55864
SGCB	3,15298
SGK1	4,06533
SGK223	5,95502
SGOL1	-2,90374
SGOL1-AS1	-5,50647
SGOL2	-3,46481
SGTB	3,78309
SH3BGRL2	-2,68769
SH3GL2	-3,3396
SH3GL3	-5,49365
SH3KBP1	-3,71328
SH3PXD2B	2,98166
SH3RF1	4,74605
SHANK3	-2,59201
SHC1	-2,26139
SHC2	2,3074
SHCBP1	-4,94117

SHOC2	-3,4451
SHROOM2	-3,67896
SHROOM4	4,09929
SIGLEC16	4,38969
SIK1	2,58868
SIMC1	3,53185
SIN3A	-4,27436
SIPA1L1	-2,51674
SIRT3	2,94752
SIRT5	3,74661
SKA3	-2,93214
SKI	-3,59318
SKP2	-4,01229
SLAIN2	-3,15582
SLAMF7	3,02536
SLBP	-7,02137
SLC13A4	-7,23439
SLC14A2	2,37728
SLC15A1	4,60864
SLC16A7	2,84826
SLC17A7	3,38832
SLC18A2	-3,95687
SLC1A5	-3,56424
SLC20A1	5,06031
SLC23A2	4,86031
SLC24A4	2,95704
SLC25A20	4,08125
SLC25A28	9,97274
SLC25A3	3,90899
SLC25A34	3,17385
SLC25A37	-2,69852
SLC25A4	3,59249
SLC25A44	-4,39717
SLC25A45	3,85401
SLC25A46	-4,80562
SLC29A1	-3,85784
SLC2A10	4,95061

SLC30A5	3,67931
SLC30A6	3,25416
SLC33A1	-4,89729
SLC35A4	3,83091
SLC35E1	3,24911
SLC35E2	3,7324
SLC35E3	3,14343
SLC35F6	4,44831
SLC35G1	-4,31727
SLC37A2	2,77961
SLC38A1	2,65245
SLC38A2	7,42509
SLC38A3	-2,67083
SLC39A10	-3,60926
SLC39A6	-2,67769
SLC3A2	4,66508
SLC40A1	6,10334
SLC43A2	3,86743
SLC43A3	-3,93091
SLC45A3	-0,53354
SLC47A1	6,86577
SLC4A7	-4,03155
SLC50A1	3,93213
SLC5A11	-3,5546
SLC5A5	2,2169
SLC5A6	-4,24681
SLC6A10P	5,68004
SLC6A16	-3,37913
SLC6A5	-4,36077
SLC7A1	7,88939
SLC7A14	4,67514
SLC7A7	-4,48906
SLC7A8	-2,11139
SLC8A1	-2,97495
SLC8B1	2,68913
SLC9A3R1	-3,5525
SLC9A7	2,64771

SLCO2A1	4,2155
SLCO3A1	-3,28416
SLIT3	-3,99401
SLMAP	5,18745
SLX4IP	-4,29757
SMAD1	-3,1735
SMARCA1	3,84211
SMARCA2	-4,01337
SMARCA4	2,20915
SMARCB1	-3,151
SMC1A	-2,85996
SMC2	-2,43442
SMC3	-2,97536
SMC4	-2,64439
SMCO4	2,87612
SMCR8	-3,47397
SMG1P7	3,75141
SMG7	-1,91378
SMIM17	3,00428
SMIM20	0,26098
SMIM3	4,58232
SMO	5,80822
SMPD3	-5,80115
SMR3A	3,55461
SMYD2	6,20901
SNAI2	6,31252
SNAP23	-2,5522
SNAPC1	3,63991
SNCG	-3,7384
SNF8	2,84282
SNRNP40	3,11413
SNRPC	-4,62691
SNTB2	4,63147
SNX12	-5,86708
SNX22	4,64117
SNX25	-3,74774
SNX4	-2,57049

SOHLH2	-7,18844
SORBS2	8,02288
SOWAHC	3,66462
SOX10	8,92826
SOX13	-4,94885
SOX15	-3,79299
SOX2-OT	3,70224
SOX30	-5,21324
SOX4	3,16951
SOX9	7,1753
SP100	4,72658
SP140L	5,87251
SP3	-3,09907
SPAG1	-4,3317
SPAG9	3,35388
SPARC	8,1497
SPATA13	5,6583
SPATA17	-2,91287
SPATA2	-6,23879
SPATS2	3,16334
SPATS2L	0,40488
SPC25	2,70654
SPCS3	5,79685
SPDL1	-7,48257
SPDYE7P	4,12263
SPECC1	-3,25299
SPEN	4,46113
SPG20	7,71807
SPICE1	-3,62589
SPIN4	6,41705
SPINT2	-3,23118
SPINT4	9,68679
SPIRE2	2,42506
SPN	2,54439
SPOCK1	2,99323
SPOP	3,72828
SPPL3	-3,6053

SPRTN	-3,92517
SPRY2	6,02625
SPSB1	3,72934
SPSB4	8,53474
SPTLC1	3,50754
SPTSSA	3,39881
SQRDL	-3,97268
SRD5A3	4,26977
SRP14-AS1	2,68827
SRPK1	-3,1363
SRPR	-2,6189
SRPRB	2,9543
SRRM4	3,43915
SRSF1	2,34897
SRSF2	7,38168
SRSF3	3,62379
SRXN1	-3,31993
SSFA2	-3,24374
SSPO	-3,62141
SSTR5-AS1	-0,49619
SSX6	4,41337
SSX7	4,99122
SSX8	-6,06139
ST3GAL2	4,92232
ST6GAL2	4,15676
ST8SIA2	3,32995
STAC2	2,70937
STAG3	-3,87154
STAM2	-2,80345
STAMBPL1	9,85181
STAR	8,24528
STARD3NL	-2,8662
STARD7	-3,97432
STAT2	5,39751
STAU1	-1,93041
STEAP2	4,39697
STIL	-2,39677

STIM2	-6,3084
STK11IP	5,95334
STK17B	3,61439
STK31	-5,01889
STK38L	-4,62199
STK39	-2,67068
STOM	3,93683
STON2	5,54676
STPG1	-4,27656
STRADA	-3,32853
STRIP1	-3,61082
STRN	4,38966
STX6	-4,43612
SUDS3	-4,55892
SUGT1	-0,47336
SUGT1P1	7,75347
SUN1	-3,45116
SUPT20H	-5,81981
SUPT20HL2	-6,24735
SUV39H2	-3,23992
SUZ12	5,36169
SVIL	5,92805
SVOPL	-4,18296
SWI5	4,01492
SYAP1	5,04621
SYCP2	-4,25087
SYCP2L	-3,48303
SYNE2	-2,97046
SYNJ1	-3,51522
SYNPO	2,6303
SYNPO2	4,08058
SYNPO2L	4,52474
SYT1	-3,23212
SYT15	4,01523
SYT17	-3,47882
SYT9	-4,92063
SYTL2	-4,52399

SYTL3	-4,99727
TAB3	5,30698
TACC3	-7,3508
TADA2A	-3,61938
TADA3	4,15454
TAF2	3,58903
TAF3	-3,81959
TAF4	-4,16803
TAF4B	-3,94848
TAF5	-4,98856
TAF5L	-5,28849
TAGLN2	-2,48211
TALDO1	-3,38365
TANC2	3,11358
TANGO2	2,60734
TAOK3	-3,57175
TAT	-2,65908
TBC1D1	-4,19698
TBC1D10B	-6,07835
TBC1D14	-3,37702
TBC1D16	6,11841
TBC1D22A	4,13211
TBC1D24	2,8691
TBC1D25	2,71336
TBC1D31	-2,47941
TBC1D5	3,33853
TBL2	2,84073
TBP	-5,38908
TBPL2	-3,06633
TBXA2R	3,54912
TC2N	-3,84376
TCEAL4	7,13022
TCEB1	2,81746
TCF20	-4,44464
TCF7	-4,54351
TCF7L1	-4,24588
TCFL5	-6,09436

TCHH	8,20685
TCHP	-2,29792
TCL1A	-8,20956
TCL6	6,25057
TCOF1	-3,99508
TCTA	4,85869
TCTE1	8,89096
TCTN1	-3,78111
TDG	3,81842
TDP1	-3,17186
TDRD1	-5,78879
TDRD5	-2,62693
TEAD1	5,36144
TECPR2	-2,958
TECTA	-2,73502
TEK	-2,82601
TENC1	9,27408
TERF1	2,86648
TERF2IP	7,35953
TES	3,73305
TEX13B	4,90522
TEX14	-3,7881
TEX15	-3,83976
TEX37	-4,17826
TFAP2D	-6,84496
TFDP1	-2,37814
TFDP2	3,07643
TFRC	3,62503
TGFB3	5,9993
TGFBRAP1	-4,27718
TGM4	-2,80523
TGOLN2	4,36472
THADA	6,02463
THAP1	-3,90391
THBS1	7,20216
THEGL	-3,20444
THEM4	0,31437

THRAP3	3,98926
THRB	-2,41976
THUMPD3-AS1	4,69357
THYN1	-3,88356
TIAM1	-7,38169
TIAM2	-4,9411
TICRR	-5,45698
TIFA	-2,66416
TIGD1	3,44617
TIGIT	4,69579
TIMM10	7,77125
TIMM44	-4,59126
TIMM50	4,30312
TIMP1	3,3777
TINCR	3,07087
TLE4	-3,96107
TLE6	-4,90007
TLN2	-2,6584
TLR5	6,34767
TM7SF3	-3,68439
TMC5	-2,47574
TMCC1	-4,23945
TMCC1-AS1	0,51528
TMED3	-2,34008
TMED5	5,08436
TMED8	4,01923
TMEM104	6,02677
TMEM108	-2,65306
TMEM109	-2,85997
TMEM120B	2,63157
TMEM125	2,88986
TMEM130	3,08511
TMEM132B	-4,54647
TMEM132C	9,40555
TMEM132D	-5,5682
TMEM136	3,34046
TMEM154	-2,01205

TMEM163	-3,36945
TMEM164	5,3004
TMEM17	4,44673
TMEM171	-6,38449
TMEM173	0,54998
TMEM180	4,32986
TMEM2	-5,23172
TMEM201	-4,68552
TMEM213	4,76985
TMEM248	-2,67804
TMEM263	1,09664
TMEM30A	0,03332
TMEM39A	-2,44728
TMEM39B	-2,29197
TMEM50B	-2,64251
TMEM57	-2,67853
TMEM60	-2,64321
TMEM63B	-2,61692
TMEM63C	-2,03538
TMEM65	2,75925
TMEM74	-4,47805
TMEM8A	4,40706
TMPO	-2,54432
TMSB10	4,73851
TMTC2	-0,22598
TNC	6,27092
TNFAIP2	6,18065
TNFAIP8L3	2,66459
TNFRSF10D	0,27872
TNFRSF13B	2,70996
TNFRSF21	2,50805
TNFSF13	-0,27694
TNFSF14	2,98038
TNFSF15	3,64328
TNKS	-2,70378
TNKS1BP1	6,93077
TNPO2	4,31321

TNPO3	-2,19983
TNRC6B	-4,52636
TNS3	0,62229
TOB2	3,86721
TOB2P1	2,60347
TOLLIP	-3,48924
TOM1L1	-2,82731
TOM1L2	3,43786
TOMM22	5,04656
TOMM7	5,00598
TOP2A	-4,42931
TOPORS	-3,16746
TOR1AIP1	2,45641
TOR1AIP2	2,53362
TOR1B	5,53574
TOR2A	-5,56925
TOR3A	-2,83411
TP53	4,73878
TP53AIP1	2,98316
TP53BP1	2,90383
TP63	-4,12537
TPD52	-3,47184
TPI1	4,69875
TPM1	-3,35255
TPM2	7,54056
TPM4	5,92983
TPMT	2,85658
TPP1	4,34691
TPRXL	-6,99744
TPT1-AS1	3,17509
TPTEP1	2,36532
TPX2	-4,49932
TRA2A	2,76826
TRAF3IP1	-3,39077
TRAIP	-2,72228
TRAK1	-3,27903
TRANK1	6,70136

TREML4	5,09217
TRIM13	3,51041
TRIM16	4,90292
TRIM2	8,94742
TRIM24	3,00563
TRIM29	-2,42995
TRIM38	0,60015
TRIM4	4,55029
TRIM51	0,21369
TRIM52-AS1	4,00523
TRIM56	7,19592
TRIM6	-3,64636
TRIM60	-4,53819
TRIM66	6,25146
TRIM77	-5,40099
TRIML1	-1,96247
TRIOBP	2,40035
TRMT10B	0,27736
TRMT12	4,35361
TRMT44	-2,98442
TRMT5	-3,40824
TROAP	-2,81443
TROVE2	0,41834
TRPM5	-0,02703
TRPV1	2,71462
TRPV6	-6,40528
TRRAP	3,55425
TSC22D1	-2,46572
TSC22D2	-3,30102
TSG1	3,50628
TSG101	-3,24477
TSHZ1	-6,12513
TSHZ2	4,14486
TSN	-4,11895
TSPAN12	2,38813
TSPAN6	5,61082
TSR2	4,11054

TSSC1	-2,91699
TSSK3	8,67476
TTC1	-3,19138
TTC23	2,69003
TTC9C	-2,50042
TTK	-8,37865
TTLL12	-2,51144
TTLL4	-2,44553
TUBA1A	6,01406
TUBA1C	-3,57188
TUBA4A	-2,8842
TUBA4B	-5,96563
TUBB2A	6,6128
TUBB2B	8,91888
TUBB8	-7,94878
TUBBP5	-4,36979
TUBG1	-4,95222
TUG1	3,29524
TULP3	-5,89183
TUSC7	4,06351
TVP23A	4,52599
TVP23C	3,27675
TWF2	9,89952
TXLNA	5,19315
TXLNG	-3,53586
TXNDC15	4,40241
TYW5	3,56255
UACA	-2,77124
UAP1L1	3,28606
UBA2	2,92335
UBALD1	-4,56699
UBAP1	-2,14476
UBE2C	-2,27184
UBE2D2	-3,28471
UBE2D3	-3,82737
UBE2D4	5,70154
UBE2E1	3,76632

UBE2G2	2,6872
UBE2I	-3,16618
UBE2J1	1,03905
UBE2M	3,07886
UBE2Q1	-2,73617
UBE2Q2	-0,05195
UBE2Q2P1	2,90589
UBE2T	-7,48441
UBE4A	3,42922
UBL7-AS1	3,2576
UBN1	-3,60145
UBOX5	4,23966
UBP1	7,80987
UBQLN1	-2,67211
UBR2	-3,82096
UBR7	-3,87066
UBXN1	4,38767
UBXN11	-3,5119
UBXN2A	3,42327
UCA1	1,19358
UCHL1	-5,04504
UCKL1-AS1	3,66567
UFM1	2,93197
UGDH-AS1	2,46309
UGT8	2,51696
UHRF1	-5,95118
UHRF1BP1L	-2,6677
ULBP1	6,88249
ULK1	3,20459
ULK2	-2,92532
UNC13C	-3,25808
UNC5C	3,44216
UNG	-6,58397
UQCR11	4,76079
UQCRB	2,85563
UQCRFS1	3,83281
UQCRH	4,41795

UQCRHL	6,38479
URB1	5,3604
USF1	2,50035
USP1	-4,09659
USP11	4,92055
USP2	-7,4792
USP28	3,47288
USP35	-2,9456
USP39	-2,69504
USP4	2,45944
USP44	-2,38461
USP46	4,10661
USP54	-3,16047
USP7	-2,86263
USPL1	2,68801
UTP14C	4,63258
UTP23	3,03899
UVSSA	4,57501
UXS1	-5,21863
VAMP2	3,12157
VAPA	2,70431
VAV3	-6,19212
VCAN	3,39712
VCPIP1	-3,34801
VDAC2	3,23811
VENTX	0,26546
VEZT	3,78483
VIM	4,38384
VLDLR	-4,08535
VMA21	0,3839
VMAC	-2,77065
VMP1	3,73879
VPS18	2,42383
VPS33A	2,83032
VPS4B	-2,66142
VPS72	-4,84319
VSIG10L	-0,2313

VWCE	5,90903
WAC-AS1	3,34057
WASH5P	4,39867
WBP4	-3,14433
WBP5	6,43862
WDFY1	-4,45656
WDFY2	3,61047
WDR45	2,62159
WDR53	-3,6461
WDR5B	3,25336
WDR7	-4,74698
WDR74	-2,35177
WDR75	-4,06279
WDR76	-3,15147
WEE2	-8,30565
WFDC2	-6,7775
WFS1	-1,92984
WHAMMP1	8,91595
WHSC1	-4,49387
WIPF2	-4,17476
WIPF3	5,55217
WISP1	7,76473
WNK3	4,20168
WNK4	2,72001
WNT3	3,19294
WNT7B	3,25983
WRN	-3,00217
WSB1	5,02506
WTAP	-4,24557
WWC3	-4,26528
WWOX	-3,52762
XAB2	2,1628
XAF1	3,26903
XKR8	4,73266
XPNPEP3	3,77275
XPOT	-2,74463
XRCC2	2,68795

XRN1	-3,92828
YAF2	3,30255
YBX2	-6,59578
YBX3	3,30918
YEATS4	-3,84011
YIPF3	1,06306
YKT6	5,22773
YPEL2	-6,25239
YPEL5	-2,73201
YWHAZ	3,12609
YY1AP1	-2,06211
ZAK	6,00144
ZAR1	-6,23935
ZAR1L	-2,14208
ZBTB14	-2,32379
ZBTB21	-3,41266
ZBTB24	-4,30318
ZBTB3	2,29852
ZBTB33	-3,64374
ZBTB34	1,12785
ZBTB39	-4,5498
ZBTB45	3,10149
ZBTB49	-2,71826
ZBTB7A	1,38715
ZBTB7C	6,50956
ZBTB8A	3,04095
ZBTB8B	-3,47253
ZC2HC1B	-4,72469
ZC3H10	-3,17557
ZC3H12C	6,80518
ZC3H12D	3,3881
ZC3H13	-2,66334
ZC3H18	-5,49138
ZC3H4	3,72079
ZC3HAV1	4,60957
ZCCHC17	-2,75778
ZCCHC2	-3,65322

ZCCHC7	-2,63724
ZDHHC1	4,10645
ZDHHC6	6,31619
ZDHHC7	-3,01285
ZDHHC8P1	2,80689
ZEB1-AS1	-2,4487
ZEB2	8,09455
ZFAND2A	-8,85374
ZFAND5	3,00682
ZFAS1	9,16205
ZFP69B	-3,6096
ZFP90	2,58481
ZFPM2	2,72981
ZFR2	-3,54258
ZHX2	-2,54285
ZHX3	-3,66255
ZIK1	-6,1708
ZIM2	-3,86295
ZKSCAN1	3,31286
ZKSCAN2	-4,38233
ZKSCAN3	2,90821
ZKSCAN4	-3,55885
ZKSCAN7	2,55873
ZMAT2	-2,88224
ZMIZ1	8,98632
ZMPSTE24	-2,69594
ZNF10	-5,19674
ZNF107	-2,67915
ZNF114	2,31143
ZNF117	4,32803
ZNF134	-3,60285
ZNF135	4,36577
ZNF141	3,48485
ZNF142	-3,29131
ZNF154	2,71071
ZNF155	-4,72902
ZNF16	-2,95442

ZNF182	-6,32126
ZNF189	4,21909
ZNF202	-3,08741
ZNF214	-3,79323
ZNF215	-0,36364
ZNF222	-2,41896
ZNF225	4,23426
ZNF227	-2,69782
ZNF24	4,03888
ZNF250	3,05999
ZNF253	3,89146
ZNF267	-3,04598
ZNF275	8,70548
ZNF280B	-4,29878
ZNF280C	-3,99308
ZNF280D	3,77695
ZNF324	-2,86007
ZNF329	-3,66659
ZNF330	-2,30018
ZNF331	3,72236
ZNF345	4,03766
ZNF354A	-3,30902
ZNF365	2,60665
ZNF37A	5,59245
ZNF394	3,23069
ZNF395	-2,83682
ZNF410	-2,94633
ZNF417	3,33504
ZNF419	-3,33304
ZNF425	-2,21227
ZNF428	8,5451
ZNF429	3,29956
ZNF43	2,72732
ZNF430	2,76241
ZNF467	4,57299
ZNF468	2,69262
ZNF492	2,46928

ZNF493	2,06801
ZNF514	5,06754
ZNF516	4,72787
ZNF521	3,35507
ZNF530	-4,10488
ZNF542P	3,07426
ZNF554	2,94287
ZNF556	2,56928
ZNF564	3,83927
ZNF570	-5,0786
ZNF571	-4,69462
ZNF574	-2,94185
ZNF577	3,46538
ZNF585A	3,17022
ZNF586	-0,29529
ZNF593	4,20921
ZNF595	-2,40804
ZNF597	-4,77742
ZNF606	2,77618
ZNF608	8,33588
ZNF609	3,23851
ZNF618	-5,18028
ZNF621	4,53309
ZNF626	2,65208
ZNF669	3,41717
ZNF671	3,57811
ZNF675	-3,9357
ZNF696	3,9788
ZNF697	2,86288
ZNF699	4,11221
ZNF708	4,68168
ZNF713	2,55105
ZNF714	3,35929
ZNF737	3,54423
ZNF746	-2,61651
ZNF761	-3,82158
ZNF776	6,68863

ZNF777	-5,64372
ZNF785	3,84826
ZNF788	-3,40473
ZNF791	5,50598
ZNF799	-3,34914
ZNF800	3,71051
ZNF818P	2,81158
ZNF829	2,64851
ZNF846	2,8199
ZNF852	-4,43991
ZNF865	3,14243
ZNF879	-2,58668
ZNF91	3,93827
ZNF93	-3,42356
ZNFX1	3,17181
ZNHIT3	3,6029
ZNHIT6	-2,6864
ZNRF2P1	3,78623
ZNRF3-AS1	4,17335
ZP1	-6,73662
ZP2	-7,8208
ZP3	-8,78693
ZP4	-5,85785
ZSCAN12P1	5,57094
ZSCAN25	-2,1908
ZSCAN5A	-4,28789
ZSWIM3	-5,60565
ZSWIM5	0,7949
ZXDB	1,02801
ZYG11B	4,57489
ZZEF1	3,17706

Supplementary Table 5. Dysregulated genes in oocytes vs surrounding cumulus cells in females ≥ 35 years.

Gene	Log2(Fold_Change
AGO2	-4,35885
A4GALT	-4,12895
AAMP	3,90945
ABCA12	-3,85651
ABCA3	-2,61536
ABCB10	-2,94105
ABCB4	-6,82984
ABCB7	-3,57739
ABCC2	-2,79819
ABCC3	2,26256
ABCC4	-2,52471
ABCC6P1	-5,05105
ABCD4	-3,81932
ABCE1	-3,86613
ABHD12B	-3,51282
ABHD17B	-3,41009
ABHD2	-2,51567
ABI1	-3,83797
ABI3BP	-3,67204
ABL1	-3,61187
ABL2	-2,78296
ABLIM2	-6,41265
ABLIM3	0,52478
ABO	6,47406
ABR	-2,42646
ACCSL	-6,30012
ACD	-2,66871
ACER2	-0,27889
ACLY	-0,22095
ACOT6	-3,07787
ACP1	-3,63531
ACPP	4,01023

ACPT	-5,93264
ACSL3	-2,73209
ACSL4	2,70902
ACTG2	-4,24389
ACTL6A	-4,67743
ACTL8	-0,69888
ACTN1	-2,79675
ACTR3	-2,54026
ACTR3B	-3,51598
ACVR1B	-5,51948
ACVR2B	-3,14722
ADA	-2,22056
ADAD1	-5,14209
ADAM10	-3,20808
ADAM17	-2,36401
ADAM19	-5,33303
ADAM2	-7,09994
ADAM20P1	4,82752
ADAM9	0,24599
ADAMTS1	0,33416
ADAMTS18	-2,66111
ADAMTS2	-3,38962
ADAMTS4	2,28663
ADAMTSL1	-2,79077
ADAMTSL5	3,59422
ADAR	-3,73829
ADCY3	-6,59498
ADCY5	-0,6171
ADCY7	-3,42997
ADCY9	-3,17936
ADD3	-4,25218
ADIPOR2	-2,21259
ADM	4,27051
ADO	-3,12175
ADPGK	-0,3492
ADRA2C	-8,88693
ADRBK2	-2,48367

ADSS	-2,22287
AFF1	-3,64546
AFF4	-3,44781
AFG3L2	-6,92761
AGFG1	-2,48578
AGFG2	-2,5064
AGGF1	-3,2137
AGK	-6,1924
AGMO	-3,78479
AGTPBP1	-2,30123
AHCTF1	-0,3774
AHCYL2	-2,5062
AHSA1	-3,88782
AIG1	4,50302
AIM1	-5,91581
AIMP2	-4,20232
AK3	1,85374
AK9	-2,9099
AKAP1	-4,67399
AKAP11	-3,26497
AKAP2	-2,08087
AKIRIN1	-2,51597
AKIRIN2	-5,91258
AKT2	-2,28505
AKT3	-2,89434
AKTIP	-5,75842
ALAD	2,6611
ALAS1	-4,68365
ALDH18A1	-4,43726
ALDH1A2	-4,43372
ALDH3A2	1,90696
ALDH5A1	-3,2426
ALDH7A1	-3,26789
ALDOC	3,50971
ALG1	-3,88417
ALG13	-3,87661
ALG1L2	-4,77874

ALG1L9P	-2,84749
ALG6	-4,89459
ALKBH1	-3,89843
ALKBH2	-4,9023
ALKBH5	-3,33389
ALMS1	-4,24877
ALS2	-2,43858
AMBRA1	-3,83176
AMD1	-4,04356
AMER1	-2,74742
AMIGO1	-5,83354
AMMECR1L	-0,30469
AMN1	-4,09051
AMOT	-5,7131
AMPD3	-3,58093
ANAPC1	-3,09406
ANK3	-4,0846
ANKDD1A	3,57225
ANKRA2	-3,58018
ANKRD13A	-2,82831
ANKRD17	-2,59882
ANKRD18A	4,37602
ANKRD27	-3,45754
ANKRD32	-6,14924
ANKRD36BP2	3,04414
ANKRD39	-0,37204
ANKRD45	-3,29978
ANKRD46	-2,83935
ANKRD6	-5,25249
ANKS1A	-6,06159
ANLN	-6,80765
ANO10	-4,21724
ANO5	-6,77488
ANO6	-2,86775
ANP32B	-3,59396
ANTXRL	-7,55756
ANXA7	-3,28667

AOX1	-3,96356
AP1M2	-3,08064
AP3M2	-2,76587
AP3S2	-4,42537
AP5Z1	0,3395
APC	-0,3396
APCDD1L-AS1	-5,24237
APEX1	-1,95079
APH1A	-2,71848
API5	-3,13569
APITD1	-4,85454
APMAP	2,52925
APOA1	3,56249
APOA1BP	-5,38136
APOBEC3C	3,53343
APOE	2,79128
APOL1	2,87376
APOO	-2,65988
APOPT1	-0,21251
AQR	-2,58862
ARCN1	-3,41513
AREG	2,76215
ARFGEF2	-4,66083
ARFIP1	-3,77179
ARG2	-2,55264
ARGLU1	0,63512
ARHGAP10	-3,21181
ARHGAP11A	-3,00545
ARHGAP12	-2,78351
ARHGAP18	-7,42274
ARHGAP20	-2,35717
ARHGAP23	6,29903
ARHGAP26	-3,48521
ARHGAP29	-3,93579
ARHGAP31	-3,07633
ARHGAP32	-2,60255
ARHGAP39	-3,3869

ARHGAP44	-4,47122
ARHGAP6	2,97179
ARHGAP8	-3,18858
ARHGDIA	-2,61274
ARHGEF12	-3,90421
ARHGEF15	-6,83417
ARHGEF26	-3,65238
ARHGEF3	-3,42974
ARHGEF40	3,95675
ARHGEF7	-2,24235
ARHGEF9	-2,93638
ARID1A	-3,10845
ARID2	-2,45514
ARID3B	-2,64822
ARID4A	-6,43734
ARID4B	-2,21195
ARIH1	-3,87396
ARL13B	-0,44486
ARL14EP	-2,88949
ARL4C	-2,24002
ARL4D	-2,73756
ARL6	-3,89332
ARL6IP5	3,18643
ARMC1	-5,8962
ARMC10	-2,70362
ARMC2	-3,95161
ARMC8	-1,93416
ARMCX4	3,75954
ARNT	-2,30255
ARPC4	-2,40541
ARRDC4	-4,18891
ARSA	-4,02785
ARSD	3,69704
ASAH2	-4,63943
ASAP2	-2,64736
ASF1A	-6,89388
ASF1B	-4,76189

ASNA1	-2,48113
ASPH	-2,0282
ASPM	-5,93756
ASTL	-5,22661
ASUN	-2,92055
ASXL2	-2,26601
ASXL3	-4,78269
ATAD1	-3,17563
ATAD2	-3,42323
ATAD2B	-3,69569
ATF2	-2,89448
ATF4	2,43079
ATF6	-0,46113
ATF7IP	-2,84566
ATF7IP2	-1,86007
ATG2B	-3,20157
ATG9A	2,50039
ATL2	-6,06168
ATL3	-2,78474
ATMIN	-2,70563
ATN1	2,43436
ATOX1	2,95401
ATP11AUN	-4,69033
ATP13A1	-4,28227
ATP1B1	-3,09557
ATP1B3	2,0947
ATP2A2	-2,7782
ATP2B1	-3,06989
ATP5F1	3,84041
ATP5J	-3,88068
ATP6AP1	3,03054
ATP6V0A1	-2,16072
ATP6V0A4	-6,53936
ATP6V1B2	-0,33672
ATP6V1C2	-4,27661
ATP7A	-5,02413
ATP8A1	-4,59295

ATP8B4	-4,40522
ATP9A	-4,1231
ATRN	-2,49644
ATXN1L	-2,10172
ATXN2	-4,23835
ATXN2L	-2,97408
ATXN3	-3,01191
ATXN7	-2,23069
ATXN7L3B	-2,43594
AURKA	-8,97304
AURKC	-4,17751
AUTS2	-2,76294
AVPI1	3,2822
AXL	2,14334
AZIN1	2,33541
B2M	2,31702
B3GALNT1	-2,67805
B3GALNT2	-3,62135
B3GALT6	3,81391
B3GNT2	-5,16258
B3GNT7	3,96887
BAALC	-4,04737
BACH1	-4,17834
BACH1-IT2	-3,74608
BAG3	-2,34026
BAG4	-0,29805
BAGE	6,93675
BAIAP2	-6,04654
BAIAP2L1	-4,85123
BAMBI	2,04052
BARD1	-5,84285
BARX2	-2,5302
BAZ1A	-5,12439
BAZ1B	-4,03662
BAZ2A	-2,73489
BBS4	-2,82957
BBS9	-2,11468

BCAR4	-6,11716
BCAS2	-2,72483
BCAT1	2,88768
BCL2L10	-8,04702
BCL2L13	-3,46138
BCL6	3,70601
BCL9	-3,98822
BCRP2	0,20872
BEAN1	-6,76911
BEX1	4,29915
BHLHB9	-2,79643
BHLHE22	-3,5416
BHLHE40	-5,62921
BICC1	-3,08262
BICD1	0,3217
BIN3	-5,93484
BIRC5	-2,63347
BLCAP	-4,07424
BLK	-5,46843
BLMH	-2,42031
BLOC1S2	-3,07782
BLOC1S3	-2,93616
BLZF1	-3,26243
BMP15	-1,09976
BMP6	-8,96244
BMPR1A	-4,04173
BMS1P20	-3,3404
BNC1	-3,85345
BNIP3	-2,83473
BNIPL	2,53526
BOD1	-6,80987
BOLA1	-4,23728
BPGM	-6,47246
BPNT1	-4,24108
BRAP	-3,68051
BRCA1	-1,89494
BRCA2	-0,55556

BRD1	-3,86337
BRD7	-2,24318
BRD8	-2,89588
BRDT	-3,76167
BRIX1	-3,11704
BRK1	-3,61214
BRPF1	-4,41698
BRWD1	-2,2403
BSG	-2,21915
BSX	-3,59369
BTBD10	-2,37676
BTBD19	3,41363
BTBD2	-2,11056
BTG2	3,50671
BTG4	-6,94331
BTRC	-4,64777
BUB1	-5,68742
BUB1B	-7,50913
BUD13	-2,83052
C10orf118	-2,45979
C10orf12	-0,37754
C10orf35	2,15444
C10orf82	-4,29108
C10orf88	-2,66152
C11orf30	-3,37813
C11orf40	-9,16591
C11orf82	-2,93555
C11orf84	-3,85899
C11orf96	3,17254
C12orf29	-4,87845
C12orf4	-4,86623
C12orf49	-4,75806
C14orf1	-2,73587
C14orf166	-2,21631
C14orf2	-3,25423
C14orf64	-1,72496
C15orf39	-4,33697

C16orf62	-3,02206
C16orf70	-2,94587
C16orf72	-6,15866
C16orf87	-2,87076
C17orf51	-4,09571
C17orf53	-5,30574
C17orf89	8,28071
C18orf61	2,27155
C18orf8	-3,75663
C19orf35	2,73105
C19orf53	2,63789
C19orf68	0,28255
C1GALT1	-3,11439
C1orf146	-6,07163
C1orf216	0,04912
C1orf220	-5,07546
C1orf226	-2,60842
C1orf54	3,50579
C1orf56	-3,55716
C1QTNF1	2,77578
C21orf59	-4,13881
C22orf46	3,44971
C2CD2	2,84139
C2CD3	-3,99876
C2CD5	-3,03057
C2orf44	-3,17598
C3orf49	-6,39248
C3orf56	-8,48621
C3orf70	-2,37617
C4orf19	-3,57909
C4orf27	-4,83872
C4orf33	-0,50326
C4orf47	-1,01897
C5orf27	-2,20361
C5orf34	-5,76395
C5orf49	-6,04651
C6orf106	-3,88396

C6orf123	2,76971
C6orf132	2,31293
C6orf164	4,68076
C6orf203	-0,43833
C6orf52	-6,73011
C7	2,60017
C7orf25	-6,41469
C7orf26	-5,06785
C7orf60	-3,28285
C8orf12	-6,03538
C8orf31	3,18108
C8orf49	5,02243
C9orf152	6,00481
C9orf16	-5,63542
C9orf171	-4,23839
C9orf41	-2,36695
C9orf91	-4,49169
CAB39	-3,08982
CABYR	-2,33632
CACNA1A	-4,3242
CACNA1C	2,40136
CACNA1C-IT2	-2,30553
CACNG8	2,27212
CACTIN	-3,85175
CADM1	-3,25082
CALB1	-3,7671
CALD1	3,69417
CALM2	-2,92325
CALML4	-3,58353
CALU	-3,81635
CAMSAP1	-4,52877
CAPN3	-2,62536
CAPN5	-0,25328
CAPN7	-2,83254
CAPRIN1	-1,57838
CAPRIN2	-3,30538
CAPZA1	-2,68414

CAPZA2	2,61151
CARD17	2,48987
CARS	2,80398
CASC1	-5,33458
CASC3	-2,77111
CASC4	2,08536
CASC5	-6,64706
CASP14	5,04821
CASP8AP2	-4,66784
CASQ1	4,92559
CATSPER2P1	4,31985
CATSPERD	-4,03741
CBFA2T3	-4,09469
CBLL1	-2,38397
CBLN4	-4,27862
CBR4	-5,24001
CBWD1	-6,96365
CBX1	-4,79044
CBX3P2	-3,52028
CBX5	-2,54479
CBX8	-3,14026
CCDC101	-1,97414
CCDC106	-1,84035
CCDC117	-3,23821
CCDC12	-5,89105
CCDC120	-2,28104
CCDC121	-5,19434
CCDC14	-2,74584
CCDC146	-4,73904
CCDC147	-7,63235
CCDC15	-7,88373
CCDC157	-4,79002
CCDC171	-3,99003
CCDC174	-4,85694
CCDC175	-4,57928
CCDC177	-1,8901
CCDC184	4,1766

CCDC25	-3,16252
CCDC27	-6,83027
CCDC34	-3,34908
CCDC39	-3,81943
CCDC50	-3,36684
CCDC53	-3,2415
CCDC57	6,00477
CCDC58	-2,52887
CCDC59	-3,71968
CCDC6	-2,6752
CCDC61	-2,63746
CCDC7	-3,60379
CCDC73	-2,69424
CCDC77	-3,55134
CCDC82	-3,36085
CCDC88C	-5,90515
CCDC9	4,49565
CCDC92	-2,42796
CCDC97	-2,53617
CCKBR	4,33337
CCL11	-1,92045
CCL16	3,1878
CCL20	6,28787
CCNA2	-4,70977
CCNB1	-7,23144
CCNB2	-5,77307
CCNB3	-6,5193
CCND2	-2,96917
CCNE1	-5,7029
CCNH	-4,48534
CCNI	-2,74484
CCNI2	-2,81453
CCNJ	-6,11472
CCNK	-3,45254
CCNO	-6,30993
CCNYL1	-5,51216
CCP110	-4,42163

CCRN4L	-9,40005
CCSAP	-3,79519
CCT2	-2,89289
CCT3	-3,47331
CCT7	3,27849
CCZ1	-4,0824
CCZ1B	-4,10831
CD207	-2,02035
CD276	3,97561
CD27-AS1	-3,75344
CD300E	2,77046
CD300LG	0,39621
CD36	-4,59301
CD46	2,59475
CD63	3,02491
CD82	-0,21831
CD83	-2,50673
CD96	2,72461
CDA	-4,68642
CDADC1	-8,20496
CDC20	-5,28651
CDC25A	-3,61618
CDC25B	-3,56549
CDC25C	-5,0902
CDC27	-2,00051
CDC37L1	-4,5808
CDC42BPA	-2,39921
CDC42BPB	-3,46851
CDC42EP4	-3,15867
CDC45	-4,33276
CDC5L	-3,56995
CDC6	-4,10069
CDC7	-5,31677
CDCA2	-5,48604
CDCA3	-4,26876
CDCA5	-2,76856
CDCA7L	-5,66453

CDCA8	-8,79409
CDH1	-6,01906
CDH11	3,57067
CDH17	-4,96355
CDH3	-5,79311
CDH4	-4,44563
CDIP1	-2,84514
CDK1	-5,77597
CDK12	-4,38277
CDK15	-3,94171
CDK2AP1	-0,22517
CDK2AP2	-2,93631
CDK5RAP3	5,74659
CDK7	-7,00749
CDK8	-2,48202
CDKN1A	2,9018
CDKN2AIP	-3,83959
CDON	-2,55991
CDR1	-5,82795
CDR2	-4,62449
CDS1	-3,40752
CDT1	-3,6286
CEBPB	3,25455
CEBPZOS	-6,53932
CECR1	2,30347
CECR2	-2,44743
CECR7	2,16895
CELA2A	-4,37273
CELF1	-2,40439
CELSR1	-3,09276
CENPA	-6,00175
CENPBD1P1	2,19589
CENPE	-4,94293
CENPI	-2,87444
CENPJ	-3,83377
CENPO	-4,19899
CENPP	-4,62076

CENPU	-4,02212
CEP120	-3,68999
CEP128	-4,81029
CEP152	-5,94472
CEP192	-3,91521
CEP250	-2,92308
CEP55	-4,30125
CEP63	-2,8955
CEP85	-3,41554
CEP97	-2,41395
CETN3	-7,28958
CFI	2,50729
CGN	-3,04591
CGREF1	-3,7406
CHAF1A	-5,7756
CHAF1B	-4,77427
CHAMP1	-4,05797
CHCHD7	-4,12352
CHD3	0,03383
CHD4	-2,32756
CHD7	-2,71239
CHD9	-5,23461
CHEK1	-8,38643
CHERP	-3,10204
CHGB	2,27593
CHMP2B	-4,36147
CHMP7	-2,37843
CHN2	-0,64472
CHRAC1	-2,92458
CHRNA5	-2,24126
CHST1	2,95828
CHUK	-3,12693
CIART	-4,49136
CIPC	-4,35551
CIR1	-6,69798
CIRH1A	-4,49616
CISD1	-2,58726

CIT	-5,67174
CITED2	2,65136
CKAP2	-5,60443
CKAP4	2,94349
CKAP5	-4,25039
CKS1B	-3,56886
CLASP1	1,91958
CLCN5	-3,04034
CLCN6	5,43481
CLDN1	6,61191
CLDND1	-0,29899
CLEC10A	-0,35013
CLEC16A	-2,96253
CLEC2A	-6,06243
CLEC2D	-2,65299
CLEC4GP1	-4,18981
CLINT1	-3,31961
CLIP1	-2,56355
CLIP4	-3,56443
CLN3	4,56118
CLN6	1,99868
CLOCK	-4,30281
CLPTM1L	-2,88852
CLPX	-4,41649
CLSPN	-4,70121
CLSTN1	-2,69349
CLTA	-2,18173
CMAS	2,4951
CMC2	-3,74221
CMKLR1	-2,03888
CMTM4	-4,01856
CMTM8	-3,26931
CMTR1	-2,53446
CMTR2	-4,24385
CNBD1	-6,26145
CNBP	-2,06658
CNIH3	-2,08539

CNKSR2	-4,20645
CNKSR3	2,59228
CNN3	-2,73951
CNNM2	-7,00393
CNOT1	-1,75438
CNOT11	-5,57248
CNOT3	-2,67873
CNOT4	-4,74811
CNOT6	-3,80168
CNOT6L	-4,11953
CNPPD1	3,75925
CNRIP1	-4,50705
CNST	-2,42792
CNTD2	-8,54293
CNTLN	-1,80721
CNTRL	-3,98499
COA1	-3,25607
COA3	2,21711
COA5	-2,31722
COBLL1	-2,61378
COG8	-0,45232
COL1A1	2,76611
COL24A1	-3,34254
COL4A1	3,71458
COL4A3BP	-4,59308
COL6A3	2,93054
COL6A6	-4,35877
COPG1	-4,17371
COPS7B	-4,57937
COPS8	-3,39681
COQ5	-3,83078
CORO1C	-2,44758
COTL1	3,17701
COX18	-4,25586
COX5B	-5,2496
COX6B1	-2,83335
COX8A	2,9922

CPEB1	-3,50855
CPED1	-3,09309
CPNE2	2,57204
CPS1	-3,50312
CPSF3	-3,47972
CPSF7	3,02989
CR2	-2,7529
CRABP1	-2,73608
CRAMP1L	-2,57859
CREB3L2	-3,07694
CREB5	-2,89782
CREBBP	-2,86022
CREBRF	-2,20121
CREG1	3,58557
CRIPAK	2,51692
CRISPLD1	-3,42531
CRKL	-3,75997
CRNKL1	-4,14953
CRNN	-3,55672
CROCC	-2,14962
CRY1	-3,90133
CRYBB2P1	3,43947
CRYZ	-2,71364
CS	-4,34323
CSDE1	-4,47458
CSE1L	-2,39627
CSF1R	-3,76937
CSNK1G3	-1,81821
CSNK2A1	-3,07732
CSRP1	-5,35638
CSRP2	-3,25669
CSTB	-2,68404
CSTF1	-6,69644
CSTF2	-3,03932
CSTL1	3,77595
CTAGE5	-2,47888
CTBP1-AS2	-4,26808

CTC1	-4,05727
CTCF	-2,38507
CTCFL	-4,06754
CTD-2151A2,1	-6,28207
CTDP1	-3,76344
CTDSPL	-3,97936
CTDSPL2	-4,69848
CTGF	3,81444
CTIF	2,69049
CTNNA1	-0,25843
CTNNA2	-4,73501
CTNNB1	-3,21071
CTNND1	-2,73082
CTR9	-2,51588
CTSK	-1,93835
CTSV	-5,23684
CTTNBP2NL	-0,44286
CUL1	-3,73631
CUL5	-0,56612
CUX1	-2,10007
CUX2	-3,76481
CWC25	-3,03281
CWF19L2	-2,86579
CXCL14	-3,19677
CXCL8	2,05173
CXorf23	-2,88531
CXorf40A	3,14295
CXorf40B	-3,11549
CXXC5	3,27701
CYB5D1	-2,88078
CYP1A2	3,41164
CYP2B7P	2,52082
CYP2E1	3,77896
CYP2F1	3,72694
CYP2S1	-2,90823
CYP4Z2P	-5,26501
CYP51A1	2,01826

CYSTM1	-0,24496
DAAM1	-4,84524
DAAM2	-3,34638
DAGLB	-3,27062
DAW1	-2,44867
DAZL	-6,94768
DBI	-2,03923
DCAF17	-2,74704
DCAF4L2	-5,77031
DCAF5	-3,64603
DCAF7	-4,02267
DCAF8L2	2,94923
DCC	-4,93401
DCDC2	-8,13213
DCLK2	-5,09579
DCLRE1A	-0,30761
DCLRE1B	-4,60518
DCP1A	-0,23779
DCP1B	-2,89979
DCP2	-4,30323
DCT	-0,58559
DCTN1	-2,93705
DCTPP1	-2,06287
DDB2	-2,86008
DDIT3	6,04008
DDX10	-3,50167
DDX11	-4,81578
DDX11L2	2,94402
DDX19A	-3,19983
DDX23	-2,87976
DDX3X	-2,73457
DDX4	-6,40177
DDX43	-4,64498
DECR1	2,33176
DECR2	3,20803
DEDD	-3,55578
DEK	-5,31621

DENND1C	-4,53517
DENND3	6,46687
DEPDC1B	-0,06939
DEPDC7	-0,92495
DERL2	-0,79976
DESI2	-3,84781
DET1	-0,31693
DFFB	-4,18556
DFNA5	-6,03459
DGKI	-2,91407
DGKQ	-5,79835
DHCR24	3,05933
DHRS11	4,7548
DHTKD1	-3,06947
DHX15	-2,16858
DHX29	-2,60028
DHX33	-2,65338
DHX8	-4,12456
DIAPH3	-2,84406
DIDO1	-0,23436
DIP2A	2,19653
DIP2B	-4,05774
DKC1	-3,14117
DLG1	-4,69637
DLG3	-3,49161
DLG5-AS1	-2,22954
DLGAP1	8,24217
DLGAP5	-1,26594
DLK1	6,02377
DLST	-5,6916
DMRTC2	-1,99595
DMTF1	-2,50556
DMTN	-3,85836
DMWD	2,18498
DMXL1	-3,90373
DNAAF2	-6,73265
DNAH11	-4,1005

DNAH12	-3,14798
DNAH17	-3,82162
DNAH3	-2,76255
DNAJA1	-3,58306
DNAJA2	-3,42042
DNAJB1	-4,79054
DNAJB11	-5,07064
DNAJB5	4,49456
DNAJC10	-2,55927
DNAJC13	-3,22054
DNAJC15	-3,96545
DNAJC16	-3,10078
DNAJC21	-2,7037
DNAJC3	-3,23429
DNAJC30	3,77153
DNAJC5G	-2,77856
DNAJC9	-2,96972
DNER	-3,02708
DNM2	3,31319
DNMBP	-3,28003
DNMT1	-8,58685
DNMT3A	-4,50337
DNMT3B	-3,30301
DNTTIP2	-2,65106
DOCK4	-4,41301
DOCK5	3,81522
DOCK9	-4,95806
DONSON	-2,52293
DOPEY1	-6,35781
DOPEY2	-3,72747
DOT1L	-3,01997
DPF2	-8,76491
DPH5	-3,2215
DPP4	2,45722
DPPA3	-9,51597
DPPA5	-1,38764
DPYSL2	-2,48162

DPYSL3	2,19605
DRAM1	3,46366
DROSHA	-2,48862
DSCAM	-3,33922
DSN1	-3,69264
DSPP	-2,62441
DTL	-6,35803
DTNBP1	-3,22099
DTX2	-3,24065
DUOX2	3,09671
DUSP10	-4,46568
DUSP11	-3,84627
DUSP14	-3,53335
DUSP23	2,72963
DUSP5	-3,11869
DUSP7	-7,68504
DUSP8	-0,23725
DYNC1I2	-2,56094
DYNC2H1	-2,43482
DYNLL1	-2,66422
DYNLL2	-3,46775
DYNLT1	-6,16218
DYNLT3	3,06383
DYRK1A	-4,76839
DZIP1	-3,54741
E2F1	-5,40039
E2F8	-3,41634
EBAG9	-5,10734
EBF1	-5,42561
EBF2	-4,61373
EBLN1	-6,59119
EBNA1BP2	2,96384
ECE1	-1,99286
ECHDC3	-2,09469
ECT2	-3,58792
EDC4	3,03069
EEF1B2	3,13142

EEF2K	-4,44283
EEPD1	-2,08814
EFCAB10	-6,44609
EFCAB14	-3,75219
EFCAB6	-4,66349
EFHD2	4,88157
EFNB2	-4,41723
EFR3B	-3,31783
EFTUD2	-2,64544
EGF	-4,25147
EGLN3	-3,88301
EHBP1	-4,53767
EHMT1	-2,53119
EI24	-2,77911
EIF2A	-2,65425
EIF3A	-2,09917
EIF3D	2,87064
EIF3K	-2,40265
EIF4A1	2,85989
EIF4A2	-2,29329
EIF4E	-3,45346
EIF4E1B	-5,81524
EIF4E3	-2,58839
EIF4EBP2	2,40678
EIF4ENIF1	-4,46302
EIF5B	-4,67721
ELAC2	-4,04198
ELAVL1	-3,67142
ELAVL2	-3,04374
ELF1	-3,90421
ELK3	-0,0459
ELK4	-2,96511
ELMO1	-6,56954
ELMO2	-4,33203
ELMSAN1	-2,51993
ELOVL4	-5,91606
ELP3	-2,03771

ELP4	-5,99721
EMD	2,04537
EMILIN2	-4,89532
EML1	-3,15563
EML4	-2,50779
EML5	-3,73418
ENDOD1	3,62145
ENG	-6,39509
ENO1	2,82169
ENO2	3,50605
ENPP2	-3,03803
ENSA	-1,56303
ENTPD6	5,79973
EOGT	-0,29559
EP300	-2,98382
EP400	-3,85032
EPAS1	-0,6401
EPB41L3	-4,50859
EPB41L4A	-2,54367
EPB41L4B	-3,42324
EPB41L5	-4,64608
EPC1	-4,29092
EPC2	-2,29981
EPCAM	-4,45346
EPN2	-3,08782
EPS15L1	-0,19436
EPSTI1	-5,20757
EPT1	-2,90414
ERAP2	-2,53321
ERBB2IP	-2,54256
ERBB3	2,83899
ERBB4	-5,54538
ERCC6	-3,61204
ERCC6L	-4,29919
ERGIC2	-4,92045
ERI1	-4,59565
ERICH5	-3,18545

ERLIN1	-4,55664
ERO1LB	-0,52649
ESCO2	-7,75297
ESF1	-3,34979
ESPL1	-4,78584
ESPNP	-6,45667
ESR2	-3,56692
ESRP1	-9,10549
ESYT1	3,79176
ESYT3	-3,45819
ETAA1	-3,23907
ETFA	-3,44979
ETFDH	-0,30725
ETV5	-7,49261
ETV6	-2,25203
EVC2	-3,9439
EXO1	-6,71437
EXOC1	-4,1617
EXOC4	-3,7824
EXOC6B	2,09161
EXOSC10	-3,66134
EXOSC3	-3,9973
EXOSC7	-4,50185
EXOSC9	-3,86905
EXTL3	-5,51598
EYA1	-3,65519
EYA2	-3,16816
EZR	-2,69215
F8A1	-2,58687
FABP5	-2,07623
FAF1	-3,04623
FAH	-4,76347
FAHD2A	-3,90667
FAM101B	2,77678
FAM102A	-6,23218
FAM110A	-4,22212
FAM110C	5,50409

FAM111B	-2,52699
FAM114A1	2,31292
FAM114A2	-2,16196
FAM115A	1,97179
FAM117A	-7,80921
FAM118B	-3,35433
FAM120AOS	-2,84947
FAM120B	-3,58779
FAM122A	-5,4986
FAM127A	6,15261
FAM129B	-2,60243
FAM131C	2,08319
FAM133B	-3,10458
FAM13A	-6,32035
FAM13B	-1,81822
FAM149A	-6,52835
FAM153C	3,00362
FAM154A	-5,47807
FAM157A	-3,04364
FAM160A1	-3,66543
FAM167A	-5,99629
FAM169A	-4,26755
FAM172BP	-0,66226
FAM174B	-4,26071
FAM175B	-2,68724
FAM184B	-4,52515
FAM188A	-2,44936
FAM189A2	-5,88526
FAM192A	-2,91754
FAM198B	2,38343
FAM199X	-2,57338
FAM19A4	-5,66987
FAM204A	-4,05992
FAM208A	-2,97881
FAM208B	-4,20799
FAM210A	-3,18109
FAM212B	-0,58957

FAM214A	-4,45949
FAM216B	2,95751
FAM21C	-2,41708
FAM220A	-8,34844
FAM222B	-6,19936
FAM228A	-7,76428
FAM45A	-4,48864
FAM46A	3,51154
FAM46B	-3,67652
FAM46C	-7,09165
FAM49B	-2,89202
FAM65B	-2,47531
FAM76A	-4,70746
FAM76B	-4,1829
FAM84B	-2,22223
FAM86A	-4,01748
FAM91A1	2,23869
FAM96A	-2,77288
FAM98B	-2,71921
FANCD2	-0,41758
FANCF	-2,12793
FANCI	-6,15422
FANCM	-2,81726
FAR2	-4,45252
FAR2P2	-2,55048
FARSA	-3,72338
FASN	2,07457
FBLN5	-4,2585
FBLN7	-0,6698
FBXL14	-5,86237
FBXL19	-3,43275
FBXL2	-3,41981
FBXO11	-2,22137
FBXO17	2,86039
FBXO30	-5,45323
FBXO32	2,18775
FBXO34	-4,97333

FBXO38	-2,10038
FBXO43	-6,04834
FBXO5	-8,51867
FBXW12	-4,69563
FDFT1	-4,24383
FER1L6	-4,98583
FERMT2	-1,81747
FEZ2	-3,69926
FEZF2	-3,57487
FGF14	-3,36675
FGF9	-3,74859
FGFR1OP	-3,13622
FGFR1OP2	-2,78109
FGGY	-5,63026
FH	-4,04516
FHIT	-3,20801
FHOD1	3,87389
FICD	-2,76924
FIGLA	-7,42733
FIGN	-1,98992
FIGNL1	-4,17108
FIP1L1	-3,63988
FJX1	3,70862
FKBP10	3,8068
FKBP15	-3,32197
FLJ10038	2,99365
FLJ12825	4,00503
FLJ16171	2,38987
FLJ30679	1,89534
FLJ31104	2,56199
FLJ42627	2,80795
FLJ43681	-4,23306
FLNA	2,14481
FLRT2	2,09132
FLYWCH2	-0,58672
FMN1	-4,92716
FMN2	-0,50932

FN3K	2,83821
FNBP1L	-3,85934
FNDC4	5,03598
FNTA	-6,30831
FNTB	-5,25095
FOPNL	-2,43234
FOSL1	5,85954
FOXI2	2,57715
FOXJ3	-3,67187
FOXK2	-2,23735
FOXM1	-5,06598
FOXN3	2,06405
FOXO3	-2,07139
FOXQ1	-4,39232
FOXR1	-6,44418
FRA10AC1	-2,61221
FRAT2	-2,57667
FRG1	-2,14912
FRMD8	3,91466
FRRS1L	-2,49113
FRS3	-2,66568
FRY-AS1	3,20509
FSCN1	3,94559
FSTL3	5,47491
FTH1	2,23746
FTSJ3	-3,19827
FUBP1	-2,47163
FURIN	-2,59122
FUT1	2,98094
FUT8	-2,71907
FXR1	-7,14658
FYN	-3,23245
FZD3	-2,5072
G0S2	3,73667
G3BP2	-2,78852
GAB1	-5,8807
GAB2	-3,83203

GABARAP	2,44023
GABBR2	-3,06421
GABPB1	-0,29813
GABRG3	-5,41171
GABRR3	-6,30857
GADD45A	2,28574
GADD45B	5,22482
GADD45G	3,66897
GAL	0,46391
GAL3ST4	2,96643
GALNS	-3,56036
GALNT3	-3,71749
GAREML	-3,36984
GAS2L3	-3,03507
GAS7	-2,20159
GATA6-AS1	2,55965
GATAD2B	-2,60011
GATM	5,49624
GBF1	-3,82952
GBP1	2,24939
GCA	-4,80572
GCC1	-4,70032
GCC2	-3,45665
GCH1	-6,81705
GCKR	4,98162
GCNT3	-6,87485
GDAP1	-0,38042
GDE1	4,28743
GDF9	-1,15055
GDI2	-0,39073
GEM	-3,34109
GEMIN5	-7,84676
GEMIN6	-2,48697
GFM1	-5,31004
GFM2	-2,95633
GFPT1	-4,67557
GGA1	3,06809

GGA3	-4,40236
GGACT	-5,21322
GGNBP2	-5,09427
GID4	-3,18911
GIGYF2	-2,14869
GINS1	-5,24889
GINS2	-6,34991
GINS3	-3,28342
GIPC2	-3,19683
GIT1	-3,21042
GIT2	-4,62044
GJC1	-3,11991
GK	3,64669
GKAP1	-3,31204
GLB1L2	-2,31494
GLCCI1	-6,86584
GLE1	-3,47602
GLI3	3,15195
GLIDR	5,19739
GLO1	3,35256
GLRX	-5,60339
GLTSCR1	-2,32969
GLUD1P3	9,78577
GLYATL1	-3,48711
GM2A	-2,14626
GMCL1	-4,79785
GMEB1	-2,55968
GMFB	-3,19379
GMPR2	-2,83169
GMPS	-4,16764
GNA11	-2,26046
GNA12	-5,88672
GNA13	-3,47587
GNA14	-6,28612
GNAI1	-3,62487
GNAO1	-5,09985
GNAS	2,60752

GNAT2	-5,95976
GNG2	-2,69339
GNPDA2	-2,43038
GOLGA3	-2,69739
GOLGA5	-3,53659
GOLGA6C	-4,25039
GOLGA7	-0,33105
GOLGB1	0,24954
GOLIM4	2,31334
GOLM1	-4,60003
GOLPH3	-3,21992
GOLPH3L	-2,6785
GOLT1B	-2,40061
GOPC	-3,41923
GORASP2	-3,82433
GOSR2	-4,36476
GP1BA	4,43902
GPATCH8	-6,20071
GPBP1	-5,6765
GPD1L	-2,15257
GPM6A	-5,89163
GPN2	-3,86144
GPR107	-2,35958
GPR133	-2,46471
GPR137B	-6,86726
GPR143	-6,41334
GPR151	-2,88904
GPR173	3,324
GPR37	-6,75244
GPR39	-7,55593
GPR55	-2,30125
GPR64	-4,65431
GPRC5A	2,92452
GPRC5B	2,9828
GRAMD1B	2,67566
GRB14	3,36494
GRB7	-5,67943

GREB1	2,63078
GREM2	2,76073
GRHL1	-6,26135
GRHL2	-4,30619
GRHL3	-4,31834
GRIN2B	-2,0628
GRM8	-3,63611
GRPEL1	-3,31836
GRWD1	-5,43821
GSDMB	3,96378
GSE1	-3,23038
GSKIP	-3,01185
GSS	5,10851
GTF2A1L	-0,7556
GTF2B	-4,34534
GTF2F2	-3,67911
GTF2H1	-3,74111
GTF2H2C_2	-4,55174
GTF3C2	-4,46679
GTF3C5	-3,82603
GTPBP4	2,51576
GTSE1	-4,76359
GXYLT1	3,35171
GYG1	-3,87862
GYG2	3,48585
GZF1	-2,47822
H2AFY	2,42959
H2AFZ	3,14012
HABP2	-6,22651
HAGH	-2,77047
HAP1	-5,72019
HARS	2,63244
HAS3	-0,5549
HAUS3	-3,72249
HAUS4	-3,04331
HAUS5	-3,92528
HAUS8	-6,73635

HCFC1	-4,01415
HDAC9	-6,39252
HDC	-2,41392
HDDC2	-2,99049
HDHD2	-3,79625
HDHD3	4,22958
HEATR1	-4,0077
HEATR5A	-3,17554
HEATR5B	-2,22405
HECTD2	-3,75199
HECW2	-4,34023
HEG1	0,399
HELQ	-4,11354
HELZ	-2,61308
HENMT1	-6,58365
HERC1	-2,42502
HERC2P4	2,78944
HERC2P9	2,27881
HERC6	-6,00848
HERPUD2	-2,68943
HES6	-4,30985
HHEX	-8,72442
HHLA2	-1,86448
HIAT1	-3,68715
HIATL1	-3,88584
HIBCH	-3,83887
HIC2	-6,4531
HID1	-2,85528
HIF1AN	-2,81579
HIGD1A	-2,4966
HINT1	-3,26153
HIP1R	-3,90528
HIPK1	-2,27458
HIRIP3	-2,36387
HIST1H1A	-1,12085
HIST1H1D	3,73435
HIST1H4H	-3,13677

HIST2H2AC	-2,37253
HIVEP1	-5,15351
HJURP	-5,92268
HKR1	-2,72228
HM13	-1,92477
HMCES	-3,5675
HMCN1	-2,12914
HMGB1	-3,29672
HMGB2	-0,286
HMGB3	-2,98011
HMGN2	-3,43228
HMMR	-5,62322
HNRNPA1P10	-4,69067
HNRNPA1P33	-5,02653
HNRNPD	-0,01758
HNRNPF	-3,11945
HNRNPU	-1,86317
HNRNPU-AS1	2,47624
HNRNPUL2-BSCL2	-3,28798
HOMER1	-0,03077
HOMER2	-5,02265
HOTTIP	1,95929
HOXA4	-3,71578
HOXC9	-4,24925
HPSE2	-2,61685
HRG	-4,40995
HRH2	-2,49053
HRSP12	3,71138
HSD17B7	-4,14025
HSD3B1	4,99742
HSDL1	-2,27283
HSDL2	2,71737
HSF2BP	-5,87989
HSF5	-3,26819
HSP90AA1	-2,34168
HSP90AB1	-2,45727
HSPA12A	2,30605

HSPA2	-7,17077
HSPA4	-2,66281
HSPA4L	-5,56414
HSPA6	-1,90045
HSPA8	-4,37772
HSPA9	-2,76164
HSPB11	-2,79252
HSPB6	4,23545
HSPG2	2,66375
HTATSF1	-3,0228
HTR1A	4,73409
HTR3C	-3,68589
HTRA1	2,63435
HUS1	-2,41569
HVCN1	-6,16565
HYLS1	-4,30729
HYOU1	-3,87253
IBTK	2,49396
ICMT	-4,39357
ICT1	2,26451
ID4	2,89068
IDE	-4,30538
IDH1	-2,49201
IDH2	-2,94012
IDI1	2,79387
IER3IP1	-0,44844
IFI27L2	3,95591
IFITM2	5,23044
IFNAR2	-3,10438
IFT52	-3,34559
IGF1	-2,45362
IGF2BP2	1,97141
IGF2BP3	-7,94963
IGFBP3	-4,59346
IGFBP4	3,34228
IGFBP5	2,06993
IGLL3P	3,76272

IGSF10	-4,99123
IGSF11	-4,30093
IK	-3,24537
IKBKAP	-3,66816
IL16	-2,58863
IL17RB	2,97823
IL22RA1	-3,90601
IL23R	-3,23498
ILDR2	-3,72001
ILF2	-2,87294
ILKAP	-2,52675
IMMT	-3,12972
IMP4	2,70603
INCENP	-3,32544
ING3	-2,54647
INHBB	4,82219
INPP4A	-2,35836
INPP5B	-3,27674
INPP5F	-4,75663
INSR	2,81187
INTS10	-2,67411
INTS6	-0,26849
INTS8	-4,84849
INTS9	-2,31638
INVS	-3,75421
IPO8	-0,06239
IQCB1	-3,65493
IQCH	-4,36535
IQCH-AS1	4,09363
IQCJ	-0,54611
IQGAP1	-2,13691
IQGAP2	-6,32115
IQGAP3	-5,16014
IQSEC1	-4,41574
IQUB	-0,97949
IRAK1BP1	-3,48234
IRF2	4,19837

IRF6	-4,23055
IRF8	-6,81755
IRGM	-3,71514
IRS2	2,26497
ISG20L2	1,99707
ITFG2	-3,09997
ITGA11	-3,22258
ITGA4	-4,53996
ITGA6	-1,92657
ITGA9	-2,63853
ITGAD	-2,01028
ITIH2	-4,21315
ITK	-2,90147
ITM2C	-3,49388
IWS1	-4,86055
JADE1	2,23155
JAG1	-6,38993
JARID2	-5,4668
JAZF1	-3,44584
JMY	-3,51793
JRKL	-4,45602
JUN	2,52117
KAL1	-3,64826
KALRN	-4,60427
KANSL3	-2,56659
KAT6A	-2,33228
KAT6B	-2,13815
KAT7	-0,03787
KATNA1	-3,95137
KATNAL1	-3,64078
KATNB1	-2,38278
KAZN	3,78054
KBTBD2	-2,12648
KBTBD7	-4,05142
KCNC4	-7,19892
KCND1	3,70657
KCNH1	-3,0451

KCNH7	-4,46524
KCNJ10	6,32607
KCNJ3	-4,55444
KCNK3	2,35246
KCNK9	-4,21323
KCTD12	3,93382
KCTD21	-2,85893
KCTD5	-5,54771
KDM1B	-6,79664
KDM2A	-1,86244
KDM3A	-3,68413
KDM4A	-0,05346
KDM6A	-3,34028
KDM6B	-3,36522
KHDC3L	-6,41339
KIAA0087	-3,37664
KIAA0195	-3,83839
KIAA0247	-5,48285
KIAA0319L	2,25013
KIAA0586	-2,20125
KIAA0922	-7,27586
KIAA0947	-2,87551
KIAA1033	-3,00938
KIAA1147	-4,69435
KIAA1211	-4,98311
KIAA1257	-7,08467
KIAA1279	-2,83449
KIAA1462	-3,64977
KIAA1524	-3,32135
KIAA1549L	-2,73909
KIAA1551	-0,23575
KIAA1671	-0,41859
KIAA1731	-3,94724
KIAA2018	-2,99903
KIDINS220	-2,95687
KIF11	-3,80204
KIF14	-4,7251

KIF15	-6,44423
KIF18A	-0,66941
KIF20A	-3,09884
KIF20B	-4,43132
KIF21A	4,33733
KIF23	-6,95175
KIF26B	-4,6164
KIF2A	-2,17598
KIF2C	-2,49422
KIF3A	-5,15409
KIF3C	-3,67403
KIF4A	-6,67958
KIT	-7,40874
KLF11	-4,95848
KLF15	2,75941
KLF5	-5,98122
KLF7	2,55674
KLF9	3,94164
KLHDC3	-2,97957
KLHL11	-2,22343
KLHL18	-4,49981
KLHL20	-3,62149
KLHL26	-4,58496
KLHL32	-4,80604
KLHL7	-4,68784
KLHL8	-3,10122
KLK4	3,34028
KLK9	8,23907
KLKP1	-2,69198
KMT2C	-4,91127
KMT2E-AS1	2,90173
KNSTRN	-4,03469
KNTC1	-2,99299
KPNA2	-5,72247
KPNA3	-2,61385
KPNA7	-1,11908
KPNB1	-1,85389

KRBOX4	3,04104
KRI1	-3,73592
KRR1	-2,30969
KRT14	-1,97566
KRT32	9,27049
KRT78	-2,98015
KRTCAP2	4,61107
KTN1	-1,80818
LAD1	-5,83501
LAGE3	-3,54822
LAMA4	3,2779
LAMB1	-3,07964
LAMC1	-2,19664
LAPTM4A	-2,32329
LAPTM4B	-2,83496
LARP1	-2,81444
LARP4	-2,76422
LARP7	-3,66998
LARS2	-4,04937
LBR	-2,74102
LCE5A	-6,82046
LCOR	-2,77844
LCP2	-3,94835
LDHAL6A	-5,6526
LDLRAD3	-3,04135
LDLRAP1	-5,79141
LEF1	-8,25863
LEF1-AS1	-5,99497
LENG8	3,16542
LEO1	-2,62069
LEPREL4	2,97563
LETM2	-3,30493
LGALS3BP	4,29356
LGALS8	-2,56673
LGMN	-3,36146
LHFP	2,78656
LHFPL2	2,57159

LHFPL5	-0,30706
LHX2	-5,71238
LHX8	-4,9289
LIG3	-4,91756
LIMA1	-6,20826
LIMCH1	-2,51428
LIMK1	-3,29912
LIMS1	1,93579
LIN28A	-3,43962
LIN9	-3,25257
LINC00032	-6,94671
LINC00052	3,24604
LINC00092	2,20462
LINC00266-1	3,92643
LINC00311	2,66439
LINC00314	9,1727
LINC00330	3,42945
LINC00368	2,55482
LINC00378	-3,80958
LINC00442	4,80163
LINC00467	-2,19627
LINC00475	-5,9187
LINC00479	-6,36071
LINC00483	9,73838
LINC00552	3,55496
LINC00638	-7,67699
LINC00641	0,33206
LINC00700	2,47848
LINC00854	-3,20378
LINC00887	-0,19768
LINC00893	2,89351
LINC00898	-3,46629
LINC01021	2,70574
LINC01069	2,05983
LINC01089	4,54454
LINC01118	-4,01846
LINC01122	-0,5192

LINC01128	-5,61278
LINC01152	-3,03959
LINC01158	-2,2902
LINC01192	4,66072
LINC01194	-3,69971
LINC01204	-2,07625
LINC01226	2,07766
LINC01252	-3,54362
LINC01278	2,17379
LIPG	-2,65999
LMAN1	-2,02339
LMBR1	-2,12828
LMBRD1	-4,12348
LMCD1	3,65817
LMNB1	-2,97415
LMNB2	-3,70454
LMO3	-4,86244
LMO7	-5,16949
LMOD3	-4,7323
LMTK3	-4,30999
LMX1B	2,68571
LOC100128554	-3,67584
LOC100129931	-2,68989
LOC100133985	3,48021
LOC100272216	3,59336
LOC100506675	-5,71252
LOC100506860	4,38948
LOC100507387	7,76702
LOC100507443	6,93279
LOC100652770	-4,57612
LOC100996255	3,71893
LOC100996385	3,74956
LOC101926892	-5,69576
LOC101926935	2,65498
LOC101927021	-3,05331
LOC101927134	-3,10936
LOC101927280	-3,28301

LOC101927421	-4,93895
LOC101927599	0,34802
LOC101927666	2,27151
LOC101927668	-5,40258
LOC101927722	-5,92443
LOC101927870	-5,50367
LOC101927873	-0,27452
LOC101927881	0,29165
LOC101927921	-2,22066
LOC101928053	-0,20305
LOC101928063	-6,34727
LOC101928940	-2,02341
LOC101929234	-2,68975
LOC101929284	6,99006
LOC101929413	-2,53303
LOC101929701	-2,25531
LOC101929717	4,68756
LOC101929767	4,55921
LOC102723769	3,63377
LOC143666	-3,70416
LOC150935	2,20797
LOC220729	0,31686
LOC283299	-4,38582
LOC283731	2,80774
LOC283788	2,12503
LOC284379	2,31887
LOC284412	2,15064
LOC284578	3,06988
LOC284648	-5,22055
LOC284865	2,38473
LOC285762	4,95093
LOC285889	-3,86426
LOC339166	-4,63569
LOC389705	-3,97451
LOC389831	3,60515
LOC389895	3,9626
LOC389906	4,93398

LOC399715	-2,87005
LOC400043	2,58513
LOC400548	2,30155
LOC441081	2,84111
LOC441204	-2,57985
LOC441454	5,03273
LOC442132	-2,09794
LOC442497	2,60232
LOC642943	3,78663
LOC643201	-2,39925
LOC643733	5,39415
LOC645166	4,58003
LOC645752	-3,59464
LOC650226	-4,16598
LOC650293	3,76835
LOC728323	-6,00621
LOC728554	-2,89644
LOC728613	3,11972
LOC728637	-6,15977
LOH12CR1	-3,24884
LOH12CR2	2,07519
LONP2	-4,3351
LOXL4	2,50771
LPAR2	2,06069
LPCAT3	2,23345
LPCAT4	-4,00513
LPHN2	2,72297
LPIN2	-3,39792
LPL	2,36404
LRCH1	-2,91076
LRCH3	-4,35956
LRIF1	-4,88064
LRP10	2,32575
LRRC16A	-3,83539
LRRC17	-7,68752
LRRC28	-4,51538
LRRC4	-3,04706

LRRC46	-3,63452
LRRC59	-2,40563
LRRC7	-6,18756
LRRC8A	-2,98574
LRRC8B	-2,55501
LRRC8C	-2,20244
LRRC8D	-3,94692
LRRC8E	-3,29882
LRRK1	-5,61735
LRRN4	-3,32778
LRRN4CL	2,49903
LRRTM4	-5,23665
LSM11	-3,30128
LSM14B	-4,21276
LSM6	-4,42415
LTN1	-2,55424
LTV1	-3,17066
LYAR	-2,87936
LYPD1	-3,65955
LYZL2	2,22552
M6PR	-6,64973
MACC1	-0,36104
MAD2L1	-5,53113
MADD	-4,47789
MAEL	-7,67464
MAGED1	2,97068
MAGEE2	-4,40988
MAGOH	-3,15102
MAK	-3,0173
MAL2	-3,22805
MALAT1	3,1668
MAML2	3,52398
MAN1A1	2,70716
MAP1B	2,39088
MAP1LC3B2	-3,43197
MAP1S	-6,99581
MAP2K1	-4,0791

MAP2K6	-5,22229
MAP3K13	2,00195
MAP3K3	-5,39622
MAP3K4	-2,32783
MAP4	-1,89555
MAP4K2	-0,42951
MAP4K3	-3,69033
MAP6D1	-3,70256
MAP7	-3,46104
MAP7D2	-4,83054
MAPK13	2,23686
MAPK4	-4,64852
MAPKAP1	-0,52087
MAPKAPK2	-2,36924
MARCH4	-3,58824
MARCH7	-2,63776
MARCH9	5,68691
MARCKS	3,11083
MARK2	-4,57594
MARK3	-2,21155
MAST4	-4,10442
MASTL	-6,09793
MAT2B	-4,99727
MAX	-3,3167
MB21D2	-4,72084
MBD2	3,21733
MBD4	-1,72083
MBIP	-4,83277
MBNL2	2,42949
MBTD1	-2,50524
MBTPS2	-3,17723
MCM10	-4,44522
MCM3	-4,46896
MCM3AP	-3,23121
MCM5	-2,45667
MCMBP	-3,62785
MCTP2	-3,70023

MDH2	-1,89248
MDM1	-0,25963
MDM4	-2,84393
MECOM	-0,26192
MED12	2,89602
MED12L	-5,43825
MED13	-5,06175
MED13L	-4,13882
MED14	-3,22676
MED15	-4,16913
MED16	-7,1883
MED19	-1,95121
MED20	-4,68037
MED21	-4,88722
MED23	-2,34432
MED24	4,64542
MED25	-2,54321
MED29	2,56748
MED6	-3,92417
MED8	-3,27421
MEF2A	3,05782
MEG3	4,21386
MEI4	-3,06945
MELK	-0,57066
MEPCE	-0,49283
MEST	-4,29606
METAP2	-3,4194
METTL9	-2,03269
MEX3C	-0,42897
MEX3D	-2,21164
MFAP1	-3,72147
MFAP3L	-3,53223
MFHAS1	-2,4455
MFN1	-2,55292
MFN2	-3,77489
MFSD2A	-3,55486
MFSD6	-2,49809

MGA	-2,90121
MGARP	2,24667
MGAT5	-2,78067
MGAT5B	-3,66351
MGC39584	5,16205
MGC72080	-4,93879
MGME1	-4,23106
MGRN1	-3,03245
MGST1	3,39717
MIA3	-2,9514
MIB1	-3,04973
MICAL2	-4,32419
MICAL3	-1,96925
MICALCL	-3,6429
MICU2	-4,27682
MID1	-6,09644
MIEN1	3,62491
MIER2	-3,62886
MINA	-4,63237
MIOS	-2,14243
MIR4454	-3,96658
MIR4461	-4,79324
MIR5186	-4,96672
MIR548I2	-2,77208
MIR612	4,25913
MIR6723	-1,94805
MIR8086	6,50824
MIS18A	-3,27051
MIS18BP1	-3,24537
MITF	-2,44345
MIXL1	-5,33165
MKNK1	-2,58551
MKNK2	-3,27565
MKRN2OS	2,19158
MLLT1	-2,49984
MLLT11	-2,65911
MLLT3	-3,88061

MLLT4	-3,40878
MMD	2,48738
MMD2	-3,02305
MMGT1	-5,37184
MMP24-AS1	3,55917
MMS22L	-2,24422
MND1	-3,10581
MNS1	-4,04435
MNT	-2,78062
MOAP1	2,2409
MOB3B	-4,36123
MOCS1	-4,31076
MON1B	-4,2879
MON2	-2,56789
MORC2	-2,36684
MORC3	0,24307
MORF4L1	-4,24384
MOS	-6,08155
MPC1	-0,35182
MPHOSPH10	-2,57944
MPHOSPH8	-3,36818
MPI	0,2921
MPL	3,49932
MPP2	-4,35712
MPP6	2,59546
MPP7	-3,24023
MPPED2	2,98295
MPV17L2	-5,68828
MRAS	-3,09921
MRC1	-6,77391
MRE11A	-6,02058
MRGPRX1	8,41615
MRPL16	2,92535
MRPL2	-3,93015
MRPL27	3,49402
MRPL32	3,01479
MRPL36	-2,42547

MRPL39	-3,40925
MRPL44	-2,73453
MRPS5	2,79599
MRRF	-4,42637
MRVI1	-2,00329
MSH2	-4,69286
MSL1	-3,59447
MSL2	-5,67743
MSL3	-2,69025
MSN	2,75734
MSX2	-5,70026
MT1G	-3,41584
MT2A	3,09794
MT3	2,66716
MTA2	-3,05496
MTERFD1	-0,81845
MTERFD2	2,88133
MTF2	-5,21022
MTFR1	-5,50091
MTFR2	-6,58307
MTIF3	-2,49281
MTL5	-6,68989
MTM1	-4,07626
MTMR14	-2,93631
MTMR3	-5,74327
MTO1	-0,03491
MTRNR2L1	-3,32221
MTRNR2L10	-2,53695
MTRNR2L2	-4,22706
MTRNR2L3	-2,99995
MTRNR2L9	-2,70967
MTRR	-3,27374
MTSS1	-3,05292
MTSS1L	-3,95627
MTUS1	-5,79441
MTUS2	-2,26862
MTX3	-2,34821

MUC13	5,82851
MUC19	-2,27678
MUC4	3,99412
MUM1L1	2,86602
MVD	5,38479
MXD1	-0,66212
MYBBP1A	-3,91301
MYBL1	-4,70472
MYBL2	-3,85294
MYC	3,20542
MYCBP	-4,05698
MYH10	-2,78177
MYH7	-4,50124
MYH9	2,77166
MYL6	3,22002
MYLIP	-0,46409
MYLK	-3,56721
MYNN	-3,29759
MYO10	-2,37733
MYO19	-4,05181
MYO1E	-2,53396
MYO5B	-2,66257
MYO7A	-3,54468
MYO9A	-2,68477
MYO9B	0,02253
MYOF	-2,94624
MYOG	-4,72108
MYSM1	-0,30734
MYT1	-2,28049
N4BP1	-3,73505
NAA11	-6,15315
NAA30	-5,42657
NAA35	-3,87894
NAA60	-2,61375
NABP1	-2,24091
NACA	2,27359
NACAD	2,84383

NADK2	-4,21011
NAE1	-3,44977
NANOS1	-4,29302
NAP1L1	-2,27872
NARF	-2,48138
NARG2	-5,5689
NARS	-3,52607
NASP	-6,19441
NAT10	-3,05695
NAT14	2,27774
NAV2	-2,34676
NAV3	-3,24242
NBEAL1	-2,96033
NBN	-0,05246
NBR1	-2,18381
NCALD	-2,32721
NCAPD3	-3,60779
NCAPG	-6,20328
NCAPG2	-4,93007
NCAPH	-4,88311
NCEH1	-8,31733
NCK2	-2,50648
NCKAP5L	-3,41234
NCOA2	-3,06634
NCOA3	-3,92288
NCOA4	-2,66063
NCOA5	-2,61107
NCOA6	-2,62851
NCOA7	-2,77518
NCRNA00185	-4,58968
NDC1	-4,28568
NDC80	-2,32911
NDE1	-2,87773
NDN	3,35379
NDRG1	-0,28064
NDRG2	3,28877
NDUFA10	-4,54022

NDUFA11	2,75426
NDUFA4	3,06776
NDUFA6	-2,65415
NDUFA6-AS1	-3,89528
NDUFA7	2,62211
NDUFAF2	2,39979
NDUFB10	3,68577
NDUFB9	2,29799
NDUFS2	2,99377
NDUFS6	-3,50573
NEAT1	3,41055
NEB	-2,92542
NECAB2	4,18149
NECAP2	-4,85787
NEDD1	-3,66067
NEDD4L	-3,30681
NEFH	-4,16077
NEFM	-3,46784
NEIL3	-5,44629
NEK1	-3,51058
NEK2	-2,68074
NEK4	-3,08855
NELFCD	3,23813
NEO1	-5,48992
NETO2	-2,76206
NEURL1B	-2,20545
NEUROD1	-2,04561
NF1	-2,30738
NFAT5	2,09397
NFATC1	-2,26751
NFATC2IP	-2,77633
NFATC3	-3,27271
NFKBIA	-4,56365
NFRKB	-3,69655
NFS1	-6,25258
NFX1	-0,28317
NFXL1	-4,77657

NFYC	-4,00933
NHLRC3	-3,31399
NHSL1	-4,88241
NIF3L1	-3,61907
NIFK	-3,9496
NIN	-2,02226
NIPBL	-2,92024
NIPSNAP3A	-3,02337
NIT2	3,22387
NKAIN1	3,4594
NKAPL	-1,79033
NKD2	-3,66559
NKX3-1	-4,59393
NLN	-2,45019
NLRP1	-3,65634
NLRP11	-3,0465
NLRP13	-1,00036
NLRP14	-3,65455
NLRP2	-8,28193
NLRP4	-1,03629
NLRP5	-6,27676
NLRP7	-4,95176
NLRP8	-4,33251
NLRP9	-9,81193
NMD3	-4,60511
NNMT	0,30342
NOC3L	-4,84403
NOC4L	-2,41309
NOD2	-4,96163
NOL4	-2,61572
NOL4L	-2,97585
NOL8	-3,04688
NOLC1	-1,89738
NOP56	-5,71309
NOP58	-0,32788
NOS1AP	-1,93184
NOTCH2	2,50295

NOX4	2,21105
NPAT	-4,23202
NPC1	-4,3633
NPFFR2	0,2432
NPM1	-2,77806
NPM2	-6,99076
NPM3	-4,41153
NPSR1-AS1	-3,54964
NPY6R	3,75685
NR2C1	-3,31847
NR2E1	-5,49635
NR3C2	-5,83828
NR6A1	-4,16915
NRAP	-3,64352
NRAS	-3,32644
NRBP1	-2,67508
NRBP2	3,91202
NSF	-0,02578
NSUN4	2,26535
NSUN6	-0,27741
NTAN1	-2,90744
NTN1	-3,54235
NTRK2	-2,44549
NTRK3	-2,33326
NUBP2	2,70614
NUDCD1	-3,54243
NUDCD2	-1,93183
NUDT10	2,56036
NUDT15	0,22524
NUDT4P1	-2,89285
NUF2	-6,0573
NUFIP1	-5,64135
NUFIP2	-2,74043
NUMB	-5,42145
NUP107	-5,15616
NUP133	-3,11868
NUP153	-4,68027

NUP214	-3,38172
NUP35	-5,59254
NUP37	-4,85498
NUP50	-3,76936
NUP85	-3,06833
NUP93	-2,48255
NUP98	-4,61291
NUPL1	-3,45448
NUSAP1	-5,02885
NXPH1	-5,46422
NXPH4	-3,62441
OAT	-3,68338
OBSL1	2,72382
OCEL1	-3,05683
ODC1	-4,2525
ODF2	-4,28458
OFD1	-3,98811
OGG1	-2,74298
OLFML1	-5,95469
OLIG2	8,49016
OMA1	-2,58925
OOEP	-1,02172
OOSP2	-8,06206
OPA1	-0,36978
OPA3	0,29195
OPRM1	-4,17604
OPTC	-2,8005
OR10Q1	2,56888
OR1E2	-4,01031
OR1N1	-4,60752
OR1Q1	-2,71423
OR2T27	0,37564
OR2T4	-2,92803
OR4K15	-2,69422
OR4N2	1,00493
OR4N3P	3,37224
OR51E1	-2,91945

OR51S1	-4,32543
OR56A5	-2,33905
OR6N2	4,75316
OR6P1	-2,00813
OR6W1P	2,32442
OR7E2P	3,71968
OR7E91P	5,20206
OR8A1	4,56515
OR8D4	-5,55097
ORC2	-4,75834
OSBP	-5,96158
OSBPL10	-6,01345
OSBPL11	-3,80473
OSBPL1A	-4,23256
OSBPL2	-2,62765
OSBPL8	-2,94691
OSBPL9	-2,74702
OSCP1	-6,43711
OSER1	-0,26355
OSER1-AS1	-3,92054
OSR2	-3,76481
OSTC	-3,96759
OSTM1	-2,69133
OTUB1	-3,26506
OTUD3	-4,85403
OTUD4	-0,42401
OTUD5	-0,24941
OTUD6B	-4,83231
OTX2	-7,80539
OVCH1	-2,74712
OXNAD1	-6,10132
OXTR	3,94254
P2RY2	2,57842
PABPC4	-2,0493
PABPN1L	-7,28764
PACRGL	-2,35094
PACS1	-3,03449

PACSIN2	-4,81853
PADI1	-4,03391
PADI6	-5,76403
PAF1	-2,39383
PAFAH1B1	-2,63635
PAFAH1B2	-1,82897
PAGE2	-4,46727
PAIP1	-0,61098
PAIP2B	-4,63425
PAK1IP1	-2,40855
PALB2	-2,96411
PALM3	-3,12034
PAM	3,33929
PANX1	-3,33963
PAPD4	-4,92614
PAPD5	-0,23797
PAPD7	-6,39267
PAPOLG	-3,66689
PAPSS1	-3,66743
PAQR6	5,83095
PARD3	-2,46892
PARD6B	-4,24668
PARL	-3,2851
PARN	-3,13593
PARP11	3,00556
PARP12	-3,66823
PARP14	2,83227
PARP16	-5,59888
PARP2	-2,91998
PARPBP	-3,76538
PARVA	-3,76499
PATE1	-3,77098
PATE2	2,48846
PATL2	-5,99171
PAWR	-4,61791
PAXIP1	-5,99665
PBDC1	3,17561

PCBP1	-0,22888
PCCB	-4,30237
PCGF6	-5,07834
PCM1	-3,03292
PCMTD2	-4,12007
PCNA	-6,76765
PCNA-AS1	-6,51722
PCNX	2,27514
PCNXL4	-2,40406
PCP4L1	-7,59891
PDCD10	-1,82669
PDCD2L	-5,16075
PDCD5	-4,23314
PDCD6	4,56072
PDCL3	-4,53344
PDE3A	-4,31704
PDE6C	-4,73988
PDE8B	-6,53041
PDE9A	-4,01799
PDGFA	-2,49702
PDIA6	-3,09691
PDIK1L	-3,60608
PDK1	-1,98217
PDK2	-7,19786
PDPK1	-3,05909
PDPN	-5,31808
PDRG1	-3,50378
PEA15	3,58268
PELI1	-5,12279
PER3	-2,90879
PEX1	-3,1726
PEX11A	-7,70595
PEX11B	-4,28307
PEX12	-4,34997
PFKFB3	-2,78754
PGAM1	3,19689
PGD	-3,20736

PHACTR1	-5,80067
PHACTR3	-5,48194
PHEX	-4,20258
PHF12	-3,09066
PHF13	-2,66151
PHF14	-2,48068
PHF3	-2,39629
PHF6	-0,46683
PHF8	-3,86674
PHLDA1	2,68584
PHLPP1	-3,91086
PHTF2	-3,98973
PI4KAP1	3,42421
PIAS1	-4,18668
PIAS2	-2,40348
PIEZO1	-3,12077
PIGC	-3,2365
PIGR	2,06128
PIGU	-4,22472
PIH1D1	3,94288
PIK3C2A	-4,37661
PIK3C2B	-2,82586
PIK3CA	-2,71328
PIK3R1	-3,27257
PIK3R2	-0,38072
PIK3R3	-6,43076
PIKFYVE	-4,20012
PIM1	-2,31951
PIM3	2,50405
PIN4P1	-4,62771
PINX1	-4,8313
PIP4K2C	-6,95856
PISRT1	-3,04607
PITHD1	-4,21515
PITRM1	-2,28697
PIWIL2	-3,08904
PIWIL3	-6,45498

PJA2	-2,36355
PKD1L3	-3,49794
PKNOX2	-3,63726
PLA2G4C	-4,73636
PLAG1	-4,22975
PLAGL2	-4,12387
PLCE1	-5,65863
PLCG2	-2,18759
PLCH2	-3,24582
PLCL1	-3,34686
PLCXD3	2,27026
PLEK2	-3,14039
PLEKHA1	-5,54438
PLEKHA5	-3,13895
PLEKHA7	-4,04069
PLEKHB1	-3,39784
PLEKHF2	-5,81922
PLEKHG1	-4,07644
PLEKHG5	-4,54638
PLEKHH1	3,44102
PLEKHM2	-2,83864
PLIN2	2,50236
PLK1	-4,20357
PLLP	-6,63193
PLOD1	5,67167
PLP2	3,19261
PLS1	-4,28478
PLXNA3	2,81198
PLXNA4	-3,00156
PLXNB2	4,10987
PLXND1	2,83111
PMS1	-3,27565
PMVK	-4,21259
PNLDC1	-6,26461
PNMA1	2,91978
PNN	-3,07287
PNRC1	4,63298

PNRC2	-3,16975
POGZ	-3,16615
POLB	-4,53788
POLD3	-4,03774
POLE	-4,86789
POLE3	-2,39243
POLG2	-3,29932
POLR1D	-3,3321
POLR1E	-2,79133
POLR2A	-3,52216
POLR2D	-2,01011
POLR2G	3,17509
POLR2H	4,11946
POLR2K	-4,08112
POLR3B	-4,00113
POLR3D	-2,95539
POLR3F	3,52319
POLR3G	-4,45053
POM121	-3,31568
POM121C	-2,71577
POM121L8P	2,89462
POMK	-4,32676
POP1	-3,21368
POR	-5,54822
PORCN	3,29847
POU2F1	-2,55787
POU4F1	-6,04555
PPA2	3,43033
PPARD	-0,40024
PPARGC1A	-5,07245
PPFIA1	-4,28529
PPFIA2	-2,83429
PPFIBP2	-3,45101
PPHLN1	-2,04966
PPIF	-3,00741
PPIG	-3,86414
PPIL1	-4,37003

PPIL4	-0,47403
PPL	-2,19585
PPM1B	-0,35337
PPM1D	-5,58569
PPM1E	-6,64519
PPM1G	-4,05469
PPP1CC	-2,31188
PPP1R12A	-2,75008
PPP1R13B	-2,97623
PPP1R14B	3,01907
PPP1R15A	3,83521
PPP1R3C	-6,86452
PPP1R3D	-3,88979
PPP1R3E	4,18882
PPP2CA	-3,06843
PPP2CB	3,0884
PPP2R2A	-2,65237
PPP2R3C	-2,92943
PPP2R5C	-2,94245
PPP2R5E	-2,92291
PPP3CA	-2,55709
PPP4R4	-3,28153
PPP6C	-4,03797
PPRC1	-6,02743
PPWD1	-2,14706
PQLC2	2,45805
PRAF2	-2,12555
PRAM1	-2,12446
PRAME	4,14059
PRC1	-6,21268
PRCC	-4,06392
PRDM10	-4,12312
PRDM2	-4,10325
PRDM4	-3,39344
PRDM6	-3,49964
PRDX4	4,92686
PRELID2	-3,47118

PREPL	-3,90915
PREX1	-0,04295
PRG1	8,72934
PRIM2	-6,66578
PRKAA1	-2,80835
PRKACA	-0,35256
PRKAG1	-2,91415
PRKAR2A	-3,36854
PRKAR2B	2,15829
PRKCDBP	3,70918
PRKCG	-2,41031
PRKCH	2,24056
PRKDC	2,43053
PRKRIR	-2,25944
PRMT5	-2,76474
PROL1	4,72279
PRORSD1P	3,44726
PROSER1	-7,71781
PRPF18	-4,66442
PRPF3	-2,52967
PRPF38A	-3,67516
PRPF4	-3,48952
PRPF40A	-4,2441
PRPH2	-3,18597
PRR14	-2,27362
PRR14L	-2,5415
PRR15	-6,04279
PRR30	4,78672
PRRG1	3,00579
PRSS23	3,70646
PRTG	-4,32006
PRUNE	-2,84211
PSAP	3,12449
PSEN2	-0,31771
PSIP1	-4,32308
PSMA2	-2,57451
PSMA4	-3,41418

PSMA5	-4,16993
PSMC1	-4,01393
PSMC3IP	-3,08323
PSMD1	-4,74818
PSMD10	-3,15613
PSMD14	-4,18586
PSMD2	-4,98615
PSMD3	-2,60958
PSMD5	-3,8883
PSMD6	-3,49949
PSMD8	2,35625
PSME3	-2,91832
PSME4	2,73628
PSMG1	-5,27578
PSMG2	-3,55442
PSMG3-AS1	-4,62477
PSPC1	-2,82611
PSTK	2,78961
PTBP2	-3,35709
PTCHD4	2,15987
PTGES	2,71534
PTGES2-AS1	2,91494
PTGES3	-0,37309
PTGFRN	-2,27364
PTK2B	-4,37586
PTN	-6,12776
PTP4A2	-2,51399
PTPLAD1	2,78668
PTPLB	-2,11596
PTPN13	-2,63937
PTPN3	-5,66921
PTPN9	-4,18475
PTPRA	-3,43544
PTPRD	-3,82159
PTPRG-AS1	-2,89522
PTRF	2,75077
PTRH2	-1,97236

PTS	4,65932
PTTG1	-8,93732
PTX3	4,91995
PUF60	-6,78436
PUS7	-6,55741
PUS7L	-3,02333
PWP1	-2,24174
PWRN1	2,03623
PXK	-3,74074
PXYLP1	-1,98653
PYGO2	-5,47262
QARS	-3,19832
QKI	-2,48274
QRICH1	-4,77867
QSOX1	5,21346
QSOX2	-2,32804
R3HDM4	-3,49969
RAB10	-2,52339
RAB11FIP3	3,61419
RAB11FIP5	-5,09363
RAB12	-3,76084
RAB13	3,75419
RAB23	-2,42078
RAB2A	-2,59929
RAB30	-4,49568
RAB36	-3,51809
RAB3GAP1	-4,82156
RAB3IP	2,44228
RAB5B	-5,55322
RAB8A	-2,37674
RABEP1	-4,05966
RABGAP1	-2,8931
RABGAP1L	-3,06579
RABGEF1	-3,57692
RACGAP1	-4,40762
RAD1	-2,70688
RAD17	-3,18275

RAD21	-2,44609
RAD50	-2,86318
RAD51AP1	-5,19097
RAD51B	-2,9466
RAD54L2	-4,28731
RAI14	-3,7759
RALA	-2,7004
RALBP1	-3,17621
RALGAPA1	2,22654
RANBP9	-3,41254
RAP1GAP2	-3,97625
RARB	-5,02127
RARS2	-2,42723
RASA1	-2,08677
RASA3	-4,64954
RASD1	0,38635
RASEF	-3,95757
RASL11A	-0,69985
RASSF3	-5,18286
RB1CC1	-2,15777
RBBP4	-3,53235
RBBP7	-3,72893
RBBP8	-3,61614
RBFOX2	-3,94658
RBM10	4,06849
RBM11	-4,25755
RBM12	-2,40319
RBM17	-4,38959
RBM19	3,26858
RBM22	-4,31705
RBM25	-2,87502
RBM26	-2,45274
RBM27	-3,92604
RBM33	-2,94533
RBM39	2,45178
RBM46	-5,56217
RBMX	-4,65894

RBMX2	-4,85734
RBP1	2,90719
RBPMS	-7,54787
RBPMS2	-5,28484
RC3H1	-4,95453
RC3H2	-2,42891
RCAN2	-2,82397
RCBTB2	-0,3737
RCC2	-5,29361
RCCD1	-0,5071
RCN1	2,40646
RCOR1	-3,95755
RDH10	-4,11202
RDH11	2,18186
REC114	-3,75798
RECQL5	-2,74815
REEP4	3,74665
RELL1	-3,61453
REM1	-2,82157
REPS2	-3,56746
RERE	-1,88612
REREP3	-3,20566
REV1	-2,41977
REXO1	-3,56496
REXO2	-2,23614
RFC2	2,88525
RFC3	-4,65679
RFWD3	-4,39971
RGPD4-AS1	5,73899
RGS2	-6,90079
RGS4	3,52099
RHBDD2	3,75983
RHBDF2	-3,39939
RHOB	2,66519
RHOT2	3,56957
RIF1	-3,08035
RIMBP2	-2,83612

RIMKLA	-3,08646
RIOK1	-5,28868
RLF	-3,6055
RMDN3	-4,65962
RMI1	-2,78323
RMI2	-4,11258
RMND5A	-4,15301
RMRP	-2,15364
RND1	-5,45395
RNF10	-3,39925
RNF111	-3,44833
RNF114	-0,37049
RNF122	-7,49474
RNF130	-6,09538
RNF138	-5,23401
RNF139-AS1	3,83981
RNF144A	3,06255
RNF146	2,79897
RNF166	-5,36551
RNF169	-3,57624
RNF17	-4,56665
RNF180	-3,88856
RNF181	-1,85994
RNF185	-6,8566
RNF19B	-4,68499
RNF2	-4,63213
RNF20	-5,48072
RNF214	-2,95363
RNF216	-2,58457
RNF216P1	-3,82689
RNF219	-4,69231
RNF32	-6,87266
RNF34	-6,32905
RNF38	-6,45806
RNF4	-4,42182
RNF40	-3,86109
RNF44	-2,92738

ROBO3	-3,59967
ROBO4	-3,49815
RORA	2,61724
ROS1	-4,23958
RPA1	-2,91731
RPA2	-4,28818
RPA3OS	-4,74004
RPAP3	-3,28171
RPF1	-4,04501
RPGRIP1L	-2,03865
RPH3A	-5,76108
RPIA	-2,55761
RPL13	0,32156
RPL13A	3,04775
RPL17	3,31417
RPL18A	3,11325
RPL19	2,37045
RPL22	2,94257
RPL26L1	-4,03493
RPL31P11	-1,77335
RPL36A	-5,61771
RPP14	-2,62676
RPRD1B	2,19652
RPRD2	-2,48151
RPS15A	2,62403
RPS23	-3,58314
RPS3	1,98196
RPS6	2,51469
RPS6KB2	2,60651
RPS6KC1	-4,46547
RPS9	3,05161
RQCD1	-5,26395
RRAGC	-3,32011
RRAS	2,68293
RREB1	-3,91731
RRM2	-4,00408
RRS1	-3,10314

RSC1A1	-3,95149
RSL24D1	-2,37682
RSPO2	-6,02732
RTCB	-3,82029
RTKN2	-2,82815
RTN4RL2	-2,19441
RTTN	-2,56008
RUFY2	2,89601
RUNDC1	2,15973
RUNDC3B	-2,72782
RUNX1T1	-5,85071
RUNX2	2,56385
RUSC1	3,44101
RYR3	-4,00974
S100A10	2,44112
S100A16	3,03455
S100A6	3,40341
S100A7A	6,53069
S100A8	-6,73789
S100PBP	-3,32398
SACM1L	-3,12136
SALL3	-6,05948
SAMD1	-4,5328
SAMD4A	-3,35929
SAMHD1	-2,55724
SAP130	-5,56995
SASH3	2,85286
SASS6	-6,36122
SAT1	3,51315
SATB2	-2,18855
SAV1	-3,07075
SBDS	0,40096
SBF1	-3,66632
SBK3	-1,89832
SCAI	-3,03839
SCAMP1	-2,23547
SCAMP4	2,86246

SCAP	-3,04238
SCAPER	-3,84024
SCARB1	2,68216
SCGB1B2P	3,15057
SCGB3A2	-0,32983
SCHLAP1	-2,51419
SCLY	-3,93355
SCN2A	-4,33987
SCN3B	4,47145
SCNN1B	-2,8129
SCUBE1	-2,29715
SDC4	2,80039
SDCBP	-3,37188
SDE2	-4,1935
SDF2L1	2,06194
SDHA	-3,47245
SDHB	-2,22282
SEC11A	3,82228
SEC22A	-3,98654
SEC23A	2,57025
SEC23B	-0,29192
SEC23IP	-3,74383
SEC24A	-4,43366
SEC24C	-0,28501
SEC24D	2,36149
SEC31B	4,02692
SECISBP2	-1,99441
SECISBP2L	-2,36163
SEL1L	-2,30944
SEMA4B	-3,28667
SEMA5A	-2,32335
SEMA6A	3,4155
SEMA6D	2,55971
SEPT10	-3,2797
SEPT11	-2,1135
SEPT2	-2,2015
SEPT8	-3,96838

SERHL2	-6,69777
SERINC5	-2,80271
SERPINA3	0,26553
SERPINB5	-2,61169
SERPINE1	4,69581
SERPINH1	3,06029
SERTAD3	-0,31127
SETDB2	-2,60176
SETX	-2,54923
SF1	-1,9226
SF3A1	-2,70888
SF3A3	-3,47332
SF3B2	-2,31129
SF3B4	-2,47442
SF3B5	0,22654
SFMBT1	-3,56348
SFMBT2	-4,3052
SFR1	-5,72731
SFSWAP	-2,32709
SFT2D2	-1,86702
SFTA1P	2,44043
SFTPA2	-2,35029
SFXN3	3,57632
SGMS2	-2,66717
SGOL2	-3,04328
SGPL1	-2,41841
SH2B1	2,56281
SH2B3	-3,28852
SH2D4A	-4,11066
SH2D6	-3,49287
SH3BGRL2	-3,82507
SH3GL2	-2,8099
SH3GL3	-3,62697
SH3KBP1	-4,49212
SH3PXD2A	-4,57731
SHD	-3,46776
SHFM1	3,03291

SHROOM2	-2,71579
SHROOM4	-2,50757
SIAH1	-4,17725
SIGLEC10	-2,83717
SIKE1	-2,57543
SIN3A	-4,73906
SIPA1L1	-2,88081
SIPA1L2	-3,27614
SIRPA	-2,72979
SIX2	-2,34804
SKA3	-4,92012
SKI	-3,26952
SKP2	-6,14733
SLAIN2	-4,89092
SLBP	-6,25693
SLC11A1	5,6555
SLC12A2	-2,44051
SLC12A3	-2,48344
SLC12A5	-3,9253
SLC13A4	-3,59838
SLC14A1	-3,00525
SLC15A4	-2,5765
SLC17A1	-2,20115
SLC19A2	-2,07841
SLC1A5	-3,39253
SLC22A2	-2,39478
SLC23A2	2,74764
SLC25A17	-3,88575
SLC25A19	-0,39006
SLC25A25	-3,71644
SLC25A28	3,03687
SLC25A3	2,46701
SLC25A30	-4,37379
SLC25A34	3,08048
SLC25A37	-3,76798
SLC25A44	-2,35638
SLC25A45	1,9484

SLC25A46	-2,99993
SLC29A1	-5,28469
SLC29A3	-4,23085
SLC2A11	3,1564
SLC2A4	2,28492
SLC33A1	-3,02722
SLC35A1	-5,19085
SLC35A2	-2,95974
SLC35A5	-2,07005
SLC35G1	-3,17666
SLC38A1	2,90647
SLC38A2	2,14976
SLC38A8	-4,59463
SLC38A9	-2,51073
SLC39A10	-5,90479
SLC39A12	-3,09766
SLC39A6	-4,3911
SLC3A2	4,13943
SLC43A3	-4,04271
SLC45A3	-3,32596
SLC4A11	-3,11978
SLC4A7	-5,79283
SLC50A1	2,20216
SLC6A16	-4,03476
SLC6A19	-2,98691
SLC6A5	-5,91842
SLC6A9	-2,39897
SLC7A1	4,16113
SLC7A4	0,9717
SLC7A5	-3,95213
SLC7A8	-3,92402
SLC8A1	-3,03471
SLC9A1	-2,38139
SLC9A3R1	-2,70308
SLCO3A1	-2,82959
SLCO4A1	-8,49348
SLU7	-2,76779

SLX4	-3,20524
SMAD1	-3,17386
SMAD2	-4,33167
SMAD5	-2,84807
SMAP1	-4,82322
SMARCA1	2,21726
SMARCA2	-5,35788
SMARCAD1	-2,1504
SMARCC2	-3,20688
SMARCD1	-3,91369
SMC1A	-4,20044
SMC1B	-3,62238
SMC2	-4,87149
SMC3	-4,38044
SMC4	-3,79269
SMEK1	-2,91085
SMG5	-2,67188
SMG6	-3,29237
SMIM3	3,0891
SMKR1	-3,13995
SMPD3	-5,18927
SMPD4	-3,96307
SMUG1	3,11303
SMURF1	-3,87153
SMURF2	-4,58052
SNAI2	2,8332
SNAPC1	-3,90182
SNF8	3,52551
SNIP1	-3,91677
SNRK	-3,86057
SNRNP70	3,41422
SNRPB2	-0,29266
SNRPE	-3,45705
SNRPF	-2,31514
SNTA1	-3,56337
SNX1	-2,44526
SNX14	-2,37191

SNX19	-0,275
SNX2	-2,89197
SNX24	-3,74944
SNX30	-2,45206
SNX4	-2,89048
SNX5	-3,60169
SOCS3	5,48021
SOCS7	-3,82755
SOHLH2	-7,10205
SORBS1	-4,0018
SORBS2	1,93569
SORD	2,96223
SORT1	-2,72623
SOWAHC	2,34588
SOX13	-5,73735
SOX30	-6,59005
SOX5	-3,63827
SOX9	3,34768
SOX9-AS1	2,55337
SP3	-2,83434
SPACA6P-AS	-3,3537
SPAG1	-0,2679
SPAG16	-4,04176
SPAG5	-2,61411
SPAG7	3,17058
SPANXD	6,44396
SPARC	2,44738
SPATA13	4,40292
SPATA16	-7,38603
SPATA2	-5,55069
SPATA6L	-4,01637
SPATA7	-4,94904
SPATS2L	2,65931
SPCS1	-3,7408
SPCS2	-2,80787
SPDL1	-7,74774
SPDYC	-5,48269

SPDYE4	-4,61788
SPDYE5	3,07585
SPDYE7P	2,35717
SPECC1	-2,46441
SPICE1	-3,16126
SPOCD1	4,27068
SPOCK2	2,72006
SPR	-3,65192
SPRR1B	3,12362
SPRY2	0,45846
SPTLC2	3,27479
SPTLC3	-2,15035
SREK1IP1	-6,79251
SRGAP3	-3,11856
SRM	2,13622
SRP14-AS1	3,19044
SRP68	-2,96393
SRPK1	-3,03787
SSBP3	-3,02584
SSH2	-3,74137
SSR2	-2,63576
SSR3	-2,45626
SSRP1	-2,5713
SSTR5-AS1	-4,14479
SSX7	3,37781
ST3GAL4	-4,11617
STAG2	-2,09759
STAG3	-3,54909
STAMBPL1	4,52512
STARD7	-4,69431
STAU1	-2,73656
STAU2	-2,62757
STC1	2,72236
STIL	-3,49102
STIM2	-3,93116
STIP1	-3,97886
STK11IP	3,36069

STK24	-3,68174
STK32A	-5,53902
STK32B	-3,11606
STK35	-3,33779
STK38	-2,40346
STK39	-5,09006
STK40	-0,37188
STOM	2,61193
STPG1	-3,57115
STRBP	-3,24392
STT3B	-2,52198
STX12	-2,37975
STX16	-2,03429
STX2	-3,82774
STX6	-4,18535
STXBP3	-2,30041
STXBP4	-2,50912
STXBP6	-3,92031
SUB1	-3,71133
SUCO	-2,76458
SUDS3	-5,96939
SUFU	-2,22579
SUMO1	-2,08007
SUMO3	2,95826
SUN1	-3,54362
SUPT20H	-2,25605
SUPT20HL2	-3,96628
SUPT7L	-3,13676
SUPV3L1	-2,0551
SUSD3	-6,05665
SUV39H2	-4,10898
SUV420H1	1,96615
SVIP	-4,99967
SVOPL	-6,19253
SWAP70	-4,0797
SWI5	2,52332
SWT1	-3,97085

SYCP2	-5,28691
SYCP2L	-5,17757
SYCP3	-9,79112
SYF2	-2,66901
SYMPK	4,85335
SYN2	-2,42878
SYNE1	-3,15817
SYNE2	-4,61319
SYNJ1	-5,61745
SYNM	-2,48631
SYNPO2	-2,71201
SYNRG	-4,33867
SYPL1	-6,37874
SYT15	3,79152
SYT16	-8,25837
SYT2	-2,72636
SYTL2	-6,10478
SYTL3	-7,39908
SYTL4	-0,24797
TAAR5	-3,83359
TACC1	-2,36316
TACC2	-2,21328
TACC3	-7,59033
TACO1	-2,24541
TADA1	-5,32707
TADA2A	-3,21883
TAF10	-5,12911
TAF13	-6,26262
TAF1C	3,5596
TAF2	-2,64652
TAF3	-4,18953
TAF4	-3,80236
TAF4B	-3,96956
TAF5	-3,95737
TAF5L	-0,45046
TAF9	-3,00448
TAF9B	-3,20675

TALDO1	-2,81306
TAOK1	-2,03014
TAOK3	-2,46902
TAPT1	-2,52196
TARDBP	-3,48423
TARS	-4,05486
TAS1R2	-3,40629
TASP1	-2,35071
TAX1BP1	-3,9279
TBC1D1	-4,52815
TBC1D10B	-3,13216
TBC1D13	4,05315
TBC1D15	-2,7473
TBC1D23	-2,71332
TBC1D2B	-3,92276
TBC1D31	-4,65977
TBC1D4	4,3798
TBC1D9	-3,66701
TBCA	-4,34319
TBCC	-2,41081
TBKBP1	-3,15205
TBL3	-2,62611
TBP	-2,40687
TBPL1	-4,91885
TBX21	3,40845
TC2N	-5,01165
TCEAL2	2,42653
TCEAL5	4,61814
TCEANC	-3,53864
TCEB3	-5,0232
TCERG1	-2,93323
TCERG1L	-4,96883
TCF12	2,88899
TCF15	-2,85899
TCF20	-5,63644
TCF3	-3,68527
TCF7	-2,53826

TCHP	-2,57471
TCL1A	-7,90522
TCL1B	-2,12592
TCN1	-2,97885
TCOF1	-2,52988
TCTN1	-4,39293
TDGF1	-5,07928
TDGF1P3	-2,21526
TDP1	-3,60925
TDP2	-2,71208
TDRD1	-7,26847
TDRD12	-6,36211
TDRD5	-8,85813
TDRD6	-3,12612
TDRD7	-3,42878
TDRKH	-3,41222
TDRP	-3,47554
TECPR2	-0,2249
TECR	-2,99116
TECTA	-3,69381
TESK1	-2,69172
TET2	-1,96513
TET3	-3,80629
TEX14	-4,37652
TEX15	-3,36141
TEX2	-0,32547
TEX9	-4,41609
TFAM	-2,70172
TFAP2B	-4,10004
TFAP2D	2,05316
TFAP2E	-2,61519
TFAP4	-3,30535
TFDP1	-3,47542
TGFB1	2,13682
TGFBR1	-3,30974
TGM3	-6,0016
TGM7	-6,67609

TGS1	-2,65496
THAP1	-3,51635
THAP4	-3,07047
THBS1	0,31202
THEMIS	-6,25469
THSD4	-4,80469
THYN1	-4,42035
TIAM1	-7,67265
TICAM1	-2,55373
TICRR	-6,43778
TIFA	-3,93633
TIGD2	-3,43879
TIGIT	-4,99511
TINF2	-2,74451
TIPARP	-3,35299
TJAP1	-3,90935
TJP1	-3,55105
TLE1	-2,93002
TLE4	-3,89985
TLN2	-3,30954
TM2D3	-4,54743
TM4SF1	3,04119
TM9SF1	-3,27418
TM9SF2	-2,45441
TMA7	6,00506
TMBIM6	-3,49479
TMCC1	-3,64124
TMCC2	-2,96929
TMED9	5,32473
TMEM104	-2,59337
TMEM109	-2,61548
TMEM110-MUSTN1	-3,05343
TMEM131	-3,0373
TMEM132B	-1,8109
TMEM132D	-4,12268
TMEM133	3,70144

TMEM138	-5,03303
TMEM141	3,82007
TMEM14B	-2,27431
TMEM150C	5,06902
TMEM154	-3,28289
TMEM163	-6,86029
TMEM167A	-5,59073
TMEM169	-2,84648
TMEM170A	-5,38318
TMEM171	-8,17044
TMEM173	-1,99586
TMEM179B	-2,46561
TMEM180	5,23907
TMEM184C	-2,59693
TMEM185B	-4,98801
TMEM194A	-2,98832
TMEM2	-6,08488
TMEM200A	-3,23278
TMEM201	-2,16545
TMEM208	2,38809
TMEM211	-2,56669
TMEM214	-3,26679
TMEM252	-2,18778
TMEM261	-5,00866
TMEM263	2,65085
TMEM27	-0,27907
TMEM39B	-5,41045
TMEM57	-2,30189
TMEM60	-3,40447
TMEM69	-3,95757
TMEM74	-5,69563
TMEM80	-6,8486
TMEM9	-3,14374
TMOD2	-3,0548
TMPO-AS1	-4,34322
TMPRSS15	-2,61036
TMSB4X	-2,83166

TMTC2	-2,40576
TMTC4	-5,12833
TNFAIP3	9,64089
TNFRSF10D	-0,21789
TNFRSF11A	-4,81919
TNFSF13	-7,01252
TNFSF18	-2,18045
TNKS1BP1	6,18953
TNKS2	-2,84215
TNNI3	2,55106
TNNT1	-2,40594
TNPO1	-3,23905
TNPO3	-2,13604
TNRC6A	-4,04097
TNRC6B	-4,9644
TNS1	3,94942
TNS4	-4,78162
TOB1	-2,95813
TOM1L1	-4,02921
TOM1L2	-5,24379
TOMM70A	-2,88424
TOP2A	-5,74325
TOPORS	-3,17474
TOR1A	-2,20414
TOR1AIP2	-2,50322
TOR2A	-3,40844
TP53INP2	-2,80849
TP63	-4,03526
TPD52	-4,02395
TPM1	-3,54605
TPM3	-2,25159
TPM4	2,30804
TPP1	2,89128
TPRXL	-8,39263
TPST1	3,20988
TPX2	-3,93111
TRAF3IP1	-3,06246

TRAF5	-2,25943
TRAFD1	-3,4126
TRAIP	-2,4903
TRAK1	-3,62139
TRAPPC10	-2,79938
TRAPPC13	-2,66426
TREML3P	-3,72036
TRG-AS1	-3,35627
TRHDE	-6,19275
TRIAP1	2,82239
TRIM16	3,64514
TRIM33	-4,85032
TRIM36	-5,32385
TRIM38	2,98254
TRIM41	-0,25933
TRIM59	-5,04058
TRIM6	-4,28972
TRIM61	-0,53121
TRIM62	-4,09212
TRIM77	-6,7569
TRIP12	-3,79615
TRMT10B	3,11398
TRMT11	-3,36594
TRMT13	-3,07412
TRMT5	-2,64856
TRNP1	-5,26458
TRNT1	-3,64993
TROAP	-2,92053
TRPA1	-5,08604
TRPC1	3,70916
TRPC4AP	-3,10614
TRPM5	-3,01603
TRPV6	-3,17537
TRUB1	-2,32745
TSC1	-3,03303
TSC22D1	-3,80955
TSC22D2	-3,8857

TSG101	-3,4152
TSGA10	-3,53108
TSHZ1	-0,02441
TSHZ2	2,81003
TSN	-2,66451
TSNAX	-2,68763
TSPAN5	-3,57701
TSPYL1	-2,29515
TSPYL2	4,58135
TSSC1	-4,23528
TSTD2	-3,16414
TTC1	-2,73158
TTC23	-2,31091
TTC28	-2,45301
TTC5	-5,21278
TTC9	-0,47039
TTC9C	-2,91043
TTF2	-2,76647
TTI1	-3,51161
TTK	-7,61464
TTLL4	-3,81705
TTYH3	2,49877
TUBA1C	-5,45687
TUBA4A	-4,97433
TUBA4B	-6,85117
TUBB4A	3,26232
TUBB8	-1,02585
TUBBP5	-5,34383
TUBD1	-1,87591
TUBE1	-3,61862
TUBG1	-3,58881
TULP3	-5,3622
TUNAR	-3,11192
TUSC2	-4,87296
TUT1	-2,82148
TWIST1	-4,79812
TWISTNB	-4,41781

TWSG1	2,09241
TXLNA	3,53838
TXLNG	-2,54319
TXNDC9	-2,63436
TXNL4B	-3,97608
TXNRD1	3,54049
TYW1	-2,73714
UBA52	-3,22935
UBA6-AS1	5,6667
UBAP1	-2,3133
UBAP2	-2,01805
UBB	-3,68379
UBE2A	-2,43853
UBE2C	-9,50741
UBE2D2	-2,48674
UBE2D3	-5,06411
UBE2E1	-4,19992
UBE2G1	-3,88723
UBE2G2	2,47796
UBE2H	-2,94947
UBE2I	-3,1736
UBE2N	-4,62324
UBE2Q1	-3,84469
UBE2Q2	-6,58104
UBE2Q2L	2,65098
UBE2S	-5,7883
UBE2T	-3,03251
UBE2V1	-2,84048
UBE3A	-3,32511
UBE3B	-3,71871
UBFD1	-3,23337
UBQLN1	-3,35765
UBR1	-2,84681
UBR2	-2,77726
UBR5	-2,50112
UBXN1	-3,55403
UBXN11	-4,3937

UCHL1	-5,27988
UCHL5	-4,02287
UFL1	-3,09294
UGT2B4	-4,29541
UHMK1	-3,60828
UHRF1	-7,81579
UHRF1BP1	-2,46247
UHRF2	-3,21507
UIMC1	-2,46691
ULBP1	4,54911
UNC13C	-5,97921
UNC50	3,7194
UNG	-5,72246
UNKL	-2,9279
UPB1	-5,20666
UPF3B	-3,0227
UPK2	8,21915
UQCC2	-2,09372
UQCRB	2,31022
UQCRC2	-2,51212
UQCRH	2,90149
USP10	-2,06272
USP14	-3,69752
USP16	-2,04504
USP18	3,71399
USP2	-6,96561
USP20	-3,19253
USP30	-6,93092
USP31	-2,64142
USP32	-2,70399
USP34	-3,19005
USP37	-2,57887
USP43	-4,15085
USP44	-4,99739
USP45	-4,56587
USP54	-3,19832
USP8	-2,05361

USP9X	-3,26671
UTP14A	-2,96497
UXS1	-5,38507
VASP	-2,89459
VAV3	-4,83824
VBP1	-4,12847
VCPIP1	-3,40758
VDAC3	-3,77209
VEGFA	3,15845
VGLL4	-3,73355
VIL1	-2,50242
VIPAS39	-3,31829
VIPR2	-2,58112
VLDLR	-4,0102
VMA21	-2,50648
VMP1	3,19352
VPRBP	-3,74565
VPS13A	-4,02408
VPS13B	-0,23804
VPS13C	-1,77643
VPS13D	-2,45911
VPS37A	-2,50833
VPS4B	-4,50087
VPS72	-0,2674
VRK1	-2,97196
VTN	5,02608
VWA2	-2,28508
WAC	-3,47802
WAPAL	-5,47937
WASH5P	4,55308
WBP11P1	-3,40643
WBP4	-2,42402
WDFY1	-4,58016
WDHD1	-5,44256
WDR1	-4,57936
WDR20	-2,70006
WDR38	-5,09399

WDR45	3,03758
WDR45B	-6,24536
WDR47	-2,07664
WDR48	-2,88485
WDR5	-3,29858
WDR53	-4,33397
WDR7	-2,85962
WDR70	-2,73792
WDR74	3,48631
WDR75	-2,97532
WDR76	-4,04337
WDR87	-6,58298
WDSUB1	-5,69289
WEE2	-9,75404
WFDC10A	6,60656
WFDC2	-8,61986
WFS1	-2,45315
WHSC1	-4,48338
WHSC1L1	-2,96263
WIPF2	-4,88003
WIPF3	3,10426
WISP1	2,67593
WISP2	3,2643
WLS	-4,9138
WNT3	1,0696
WNT8B	-2,89813
WRB	-4,72371
WRN	-3,91305
WTAP	-2,50695
WTH3DI	-3,09638
WWOX	-3,39766
WWTR1	3,35318
XPC	-2,68834
XPO4	-4,14464
XPO5	-5,03217
XPO6	-2,58851
XPR1	-2,48456

XRCC6	-2,19739
XRN1	-4,46867
XRN2	-2,58201
XXYLT1	-2,59904
XYLB	-3,33677
YAE1D1	-4,40586
YAF2	2,34956
YAP1	-3,39429
YBX2	-4,60936
YBX3	2,04618
YEATS4	-7,95456
YES1	-4,32793
YIPF3	4,61073
YKT6	2,82894
YPEL2	-3,56448
YPEL5	-6,14769
YTHDF1	-0,33842
YWHAB	-4,46838
YWHAH	-2,57917
YWHAQ	-6,64071
YY1AP1	-0,20401
ZAR1	-8,05766
ZAR1L	-6,02504
ZBED5-AS1	-2,41184
ZBTB10	-2,58504
ZBTB11	-3,36808
ZBTB2	-0,26365
ZBTB21	-3,3367
ZBTB26	5,47959
ZBTB33	-4,79159
ZBTB39	-3,95548
ZBTB44	-2,70811
ZBTB49	-5,39927
ZBTB6	-3,38576
ZBTB7A	4,57972
ZC2HC1B	-4,29486
ZC3H10	-2,62532

ZC3H11A	-2,06214
ZC3H13	-3,04721
ZC3H14	-3,63233
ZC3H18	-4,17041
ZC3H3	-2,89657
ZC3H4	-2,58848
ZC3H7A	-2,65061
ZC3HC1	-3,04057
ZCCHC14	-3,09975
ZCCHC17	-2,42057
ZCCHC2	-0,58833
ZCCHC7	-4,54312
ZCCHC9	-3,62109
ZDHHC6	2,83797
ZDHHC7	-4,58276
ZDHHC8P1	3,04956
ZDHHC9	2,35474
ZEB2	1,77838
ZFAND2A	-7,03238
ZFAND5	1,80173
ZFAND6	-2,84304
ZFAT	-2,81102
ZFHX3	-2,99458
ZFYVE9	-0,27918
ZGRF1	-3,30937
ZHX1	-3,88161
ZHX3	-4,5466
ZKSCAN2	-4,08274
ZKSCAN7	2,47235
ZMAT1	2,91067
ZMIZ1	2,88985
ZMYM2	-3,76388
ZMYND11	2,03597
ZMYND8	-3,73806
ZNF10	-2,81663
ZNF101	-2,6788
ZNF107	-2,25883

ZNF114	3,20199
ZNF131	-2,63639
ZNF134	-3,75929
ZNF136	-3,31673
ZNF14	-5,33613
ZNF142	-4,96264
ZNF143	-4,15507
ZNF146	-2,23082
ZNF155	-4,32331
ZNF18	-4,0176
ZNF182	-2,79645
ZNF189	2,1762
ZNF19	-0,46536
ZNF2	-4,92555
ZNF200	-5,28539
ZNF202	-4,39116
ZNF214	-3,35544
ZNF215	-6,10481
ZNF217	-2,81299
ZNF22	-5,34459
ZNF222	-3,20649
ZNF227	-3,23418
ZNF232	-2,69686
ZNF236	-3,60685
ZNF24	2,93463
ZNF253	4,00428
ZNF254	0,4155
ZNF267	-4,54403
ZNF268	-1,84266
ZNF275	3,06603
ZNF280B	-5,48286
ZNF280C	-5,63274
ZNF281	-2,83897
ZNF302	-3,72887
ZNF304	-4,29919
ZNF330	-4,10499
ZNF354A	-4,80809

ZNF354B	-3,14248
ZNF37BP	4,16347
ZNF382	-2,43945
ZNF385B	-2,19116
ZNF391	-3,42111
ZNF397	-2,45491
ZNF407	-3,43891
ZNF415	-2,88846
ZNF416	-5,72525
ZNF419	-3,45221
ZNF430	-2,43744
ZNF438	-2,33326
ZNF443	-3,72508
ZNF449	-5,95634
ZNF462	-2,92078
ZNF468	-2,51598
ZNF500	-3,59481
ZNF502	-4,75002
ZNF506	-2,94128
ZNF507	-3,53059
ZNF512	-3,01226
ZNF514	4,16298
ZNF516	3,55164
ZNF527	2,37841
ZNF530	-0,21054
ZNF541	-3,91513
ZNF549	-3,42094
ZNF555	-3,43336
ZNF556	2,66546
ZNF557	-2,84261
ZNF559	-3,1273
ZNF560	-3,12975
ZNF561	-3,47542
ZNF564	-2,95151
ZNF569	-3,84182
ZNF57	-4,63173
ZNF570	-4,6895

ZNF571	-3,77645
ZNF574	-4,3385
ZNF584	-5,07466
ZNF585A	-4,0333
ZNF586	-4,00119
ZNF589	-4,82287
ZNF592	-4,80658
ZNF595	-3,1305
ZNF597	-5,55731
ZNF606	-7,08629
ZNF608	4,7179
ZNF610	-3,57675
ZNF618	-6,19458
ZNF623	-4,42899
ZNF638	-1,97352
ZNF641	-3,15502
ZNF644	3,13234
ZNF649	-3,39352
ZNF652	-3,49858
ZNF654	-4,02962
ZNF655	-2,69321
ZNF662	-3,18914
ZNF664	-2,9142
ZNF670-ZNF695	-4,66362
ZNF689	-5,1057
ZNF7	3,02085
ZNF720	-1,95212
ZNF727P	-2,82417
ZNF728	-2,78908
ZNF732	-4,66451
ZNF736	-3,06242
ZNF746	-5,55219
ZNF747	-3,33113
ZNF761	-2,07493
ZNF777	-4,73578
ZNF788	-0,49667
ZNF792	-3,30597

ZNF799	-3,63247
ZNF815P	-3,40658
ZNF823	-6,25558
ZNF836	-2,2269
ZNF841	3,10515
ZNF850	-2,33596
ZNF865	2,28091
ZNF93	-5,66711
ZNRF3	-2,25516
ZP1	-5,72957
ZP2	-8,71714
ZP3	-9,2249
ZP4	-5,98578
ZRSR2	-2,82582
ZSWIM3	-6,18114
ZSWIM5	3,28564
ZSWIM6	5,72664

Supplementary Table 6. Genes coding for receptors expressed exclusively in oocytes < 35 years.

**Gene**
OR10J3
OR10V1
OR4C13

Supplementary Table 7. Genes coding for receptors expressed exclusively in oocytes ≥ 35 years.

**Gene**
ADRA2C
ANTXRL
GPR55
IL22RA1
OPTC
OR4K15
OR56A5
OR6P1
OR8D4
TAAR5
TAS2R16

Supplementary Table 8. Dyregulated genes coding for receptors more expressed in oocytes <35 years.

**Gene**
ADRA2B
BMPR2
CACNG7
CMKLR1
CSF1R
FZD3
GRM4
GRM6
JMJD6
LMTK2
MCAM
OR1N1
OR2AG2
OR2M3
OR6C68
OR8G2
PLXNA3
REEP4
ROS1
SHANK3

Supplementary Table 9. Dysregulated genes coding for receptors more expressed in oocytes ≥ 35 years.

**Gene**
ATRN
CACNG2
CBLB
CLEC4D
DERL2
EPHA4
EPHB1
F2R
F3
FFAR4
FLT1
GLDN
GLUD1
HTR3C
KDELR1
LAMA1
LAMB2
LAMB2
LGR4
MRGPRX3
OLFML1
OR1E2
SCARB2

SORT1
SSR3
TAS1R2
TBXA2R
VIPR2

Supplementary Table 10. Features of the subjects enrolled in this study.

Age group	Subject	Age	BMI	AFC	Hormonal treatment
≥35 years					
	1	39	21.5	8	rFSH
	2	40	27.7	12	rFsh+rLH
	3	42	19.4	20	rFSH+HMG
	4	36	29.4	14	rFsh+rLH
	5	36	20.3	18	rFSH
	6	41	29.4	16	rFsh+rLH
	7	35	18.0	7	rFSH+HMG
	8	40	20.8	8	rFsh+rLH
	9	36	22.0	11	rFSH
	10	40	25.5	11	rFSH+HMG
	11	36	22.5	7	rFsh+rLH
<35 years					
	12	33	19.3	10	rFsh+rLH
	13	19	25.0	18	rFSH
	14	30	19.5	10	rFsh+rLH
	15	32	21.3	16	rFsh+rLH
	16	33	18.9	6	rFSH+HMG
	17	32	21.6	8	rFSH
	18	29	23.9	10	rFSH
	19	29	25.0	20	rFSH
	20	27	20.3	12	rFSH

Supplementary Table 11. Number of both reads and duplicates in oocytes and cumulus cells

Sample	Oocyte		Cumulus Cells	
	Number of reads	Number of duplicates (%)	Number of reads	Number of duplicates (%)
1	37838571	59.86	41093490	12.68
2	44541743	27.92	58331855	29.19
3	49471813	48.51	51431215	21.33
4	38720058	54.97	39773641	47.28
5	39123665	56.69	42231198	45.05
6	42311589	59.99	43414368	23.23
7	71689716	76.9	17797393	22.66
8	36201360	80.33	28044171	29.46
9	37367709	75.5	36347000	40.38
10	30228609	75.69	58865408	46.46
11	17477551	69.84	36948487	72.53
12	39006077	59.85	49313514	23.5
13	39807989	46.97	46875004	62.37
14	41194535	59.97	44646118	24.52
15	40384274	68.03	46091988	23.92
16	95705355	85.78	73837864	81.03
17	36329446	64.52	34530281	39.26
18	36380583	72.01	29017397	54.29
19	28347827	77.29	89325441	64.39
20	19353589	85.52	32039900	42.85

Supplementary Table 12. Primers used for validating RNA-seq data by RT-qPCR

Gene	Forward	Reverse
BMPR2	CACCACTCAGTCCACCTCAT	CTGCTGCCTCCATCATGTTC
CACNG2	GGAGAACCTGCTGCCTAGAA	CAGACACGAAGAAGATGCCG
FZD3	ACCAACAGACAGCAGCTTTG	GGCCAAGGAACACCAAACAT
GRM4	CGTCAACTTCTCAGGCATCG	TCTAAGGTGCAGGTGGTCAG
GRM6	TGGAACTATGTGTCCACGCT	CAAAGATGATGATGCCCCGG
LGR4	ATTGCCTGCTTGCCTGAATC	TGAGTACATGCCACAGTCGT
MCAM	CGGCAAGTGAACAAGACCAA	GTCTGGTGTGAGGGTGGTTA
